# Corticotropin-Releasing Hormone (CRH) Gene Family Duplications in Lampreys Correlate With Two Early Vertebrate Genome Doublings

**DOI:** 10.3389/fnins.2020.00672

**Published:** 2020-07-30

**Authors:** João C. R. Cardoso, Christina A. Bergqvist, Dan Larhammar

**Affiliations:** ^1^Comparative Endocrinology and Integrative Biology, Centre of Marine Sciences, Universidade do Algarve, Faro, Portugal; ^2^Department of Neuroscience, Science for Life Laboratory, Uppsala University, Uppsala, Sweden

**Keywords:** gene duplication, tetraploidization, lamprey, paralogon, CRH

## Abstract

The ancestor of gnathostomes (jawed vertebrates) is generally considered to have undergone two rounds of whole genome duplication (WGD). The timing of these WGD events relative to the divergence of the closest relatives of the gnathostomes, the cyclostomes, has remained contentious. Lampreys and hagfishes are extant cyclostomes whose gene families can shed light on the relationship between the WGDs and the cyclostome-gnathostome divergence. Previously, we have characterized in detail the evolution of the gnathostome corticotropin-releasing hormone (CRH) family and found that its five members arose from two ancestral genes that existed before the WGDs. The two WGDs resulted, after secondary losses, in one triplet consisting of CRH1, CRH2, and UCN1, and one pair consisting of UCN2 and UCN3. All five genes exist in representatives for cartilaginous fishes, ray-finned fishes, and lobe-finned fishes. Differential losses have occurred in some lineages. We present here analyses of CRH-family members in lamprey and hagfish by comparing sequences and gene synteny with gnathostomes. We found five CRH-family genes in each of two lamprey species (*Petromyzon marinus* and *Lethenteron camtschaticum*) and two genes in a hagfish (*Eptatretus burgeri*). Synteny analyses show that all five lamprey CRH-family genes have similar chromosomal neighbors as the gnathostome genes. The most parsimonious explanation is that the lamprey CRH-family genes are orthologs of the five gnathostome genes and thus arose in the same chromosome duplications. This suggests that lampreys and gnathostomes share the same two WGD events and that these took place before the lamprey-gnathostome divergence.

## Introduction

The corticotropin-releasing hormone (CRH) family consists in vertebrates of five structurally related neuropeptides that are involved in the regulation of physiological response to stress, emotional behavior, and anxiety (Vale et al., 1981; Dunn and Berridge, 1990; Koob and Heinrichs, 1999; Lovejoy and Balment, 1999; Gysling et al., 2004; Fox and Lowry, 2013). Two are named CRH (CRH1 and 2) and three are named urocortin (UCN1, 2, and 3). They have evolved through distinct pressures during the vertebrate radiation, as reflected in their differences in evolutionary rates of amino acid change (Hwang et al., 2013; Grone and Maruska, 2015b; Cardoso et al., 2016; Endsin et al., 2017). CRH (now named CRH1) was the first family member to be discovered. It was isolated from sheep hypothalamus and consists of 41 amino acids in mammals (Vale et al., 1981). Homologs of mammalian CRH1 were subsequently found in numerous other tetrapods. Duplicate CRH1 genes now named crh1a and crh1b have been described in teleosts (Hwang et al., 2013; Lovejoy and de Lannoy, 2013; Grone and Maruska, 2015a, b; Cardoso et al., 2016) and were found to have arisen as a result of the teleost-specific genome duplication (Jaillon et al., 2004). The UCN1 peptide was the second family member to be discovered in mammals and was found to be the ortholog of the previously reported bony fish urotensin and of the amphibian sauvagine. Two additional urocortins were discovered *in silico* in mammals and named UCN2 (Reyes et al., 2001) and UCN3 (Lewis et al., 2001), both of which are 38 amino acids long in mammals. They were soon found in other classes of vertebrates, including ray-finned fishes. CRH2 is the most recently discovered member and was initially identified in cartilaginous fish and was suggested to be specific to these species (Nock et al., 2011), but subsequent reports demonstrated its presence in other vertebrate classes with the exception of placental mammals and teleosts (Grone and Maruska, 2015a; Cardoso et al., 2016).

The CRH family is one of the oldest metazoan peptide families, with homologs described in several invertebrate genomes. The closest relatives of vertebrates, the invertebrate deuterostomes such as the tunicates (*Ciona intestinalis* and *Ciona savignyi*), the cephalochordates (amphioxus *Branchiostoma floridae*), and ambulacrarians (the echinoderm *Strongylocentrotus purpuratus* and the hemichordate *Saccoglossus kowalevskii*), all have a single CRH-like gene (Kawada et al., 2010; Mirabeau and Joly, 2013). Protostomes, such as arthropods, have a related peptide named diuretic hormone 44 (DH44) (Audsley et al., 1995; Cabrero et al., 2002; Lovejoy and de Lannoy, 2013).

Diverging scenarios have been proposed to explain the origin and evolution of the CRH family in relation to the emergence of the vertebrates (Hwang et al., 2013; Cardoso et al., 2016; Endsin et al., 2017). Lovejoy and coworkers used sequence analyses to arrive at a scheme with five independent gene duplications followed by one loss (Endsin et al., 2017). However, their study did not consider adjacent genes to check for duplication of large chromosomal blocks. Already before their report, we had concluded that the five members of the gene family were established early in vertebrate evolution prior to the radiation of the gnathostomes, as based on phylogenetic sequence analyses and comparisons of gene synteny and duplicated chromosomes (Cardoso et al., 2016). The comparisons of neighboring genes showed that the two CRH subfamilies are located in different paralogons, i.e., in different sets of related chromosomal regions, with the CRH1/CRH2/UCN1 subfamily members located in a paralogon also harboring opioid peptide genes and the paralogon with the UCN2/UCN3 subfamily located in the paralogon that contains the visual opsin genes (Cardoso et al., 2016). Subsequently, the two pre-gnathostome whole genome duplications (WGD, see below) (Nakatani et al., 2007; Putnam et al., 2008) resulted in chromosome duplications that turned the first gene into three copies on separate chromosomes and the second gene into two copies on separate chromosomes. All five ancestral genes have been retained in slowly evolving lineages represented by the coelacanth (*Latimeria chalumnae*, a lobe-finned fish that diverged basal to the tetrapods), the spotted gar (*Lepisosteus oculatus*, a basal ray-finned fish that radiated prior to the teleost expansion), and the elephant shark (*Callorhinchus milii*, belonging to the holocephalans among cartilaginous fishes). Gene losses have occurred in some lineages (Cardoso et al., 2016).

The evolutionary origin of the gnathostomes is considered to have been preceded by two WGD events (Nakatani et al., 2007; Putnam et al., 2008), often referred to as 1R and 2R for the first and second round of genome doubling. However, the exact timing of these events in relation to the preceding divergence of vertebrates into the gnathostome and cyclostome lineages has been difficult to resolve. Investigation of their genomes can offer important insights into the origin and evolution of genes and gene families as well as the genomic events that have shaped vertebrate genomes. The cyclostomes, or living agnathans, consist of two major extant lineages, namely the lampreys and the hagfishes. To date, four sequenced agnathan genomes are available. Two genome assemblies are from the sea lamprey (*Petromyzon marinus)*, one of which is a somatic genome from adult liver and the other a recently assembled germline genome, which is essential because somatic lamprey cells delete much of the genome in adult tissues (Smith et al., 2013, 2018). One assembly is from the Arctic lamprey (*Lethenteron camtschaticum*, formerly known as *Lethenteron japonicum*) and was obtained from mature testis (Mehta et al., 2013). Finally, a fragmentary genome has been assembled for the inshore hagfish (*Eptatretus burgeri*)^1^. Nonetheless, analyses of agnathan gene families and genome segments have been inconclusive regarding the temporal relationship between the two WGD events and the cyclostome-gnathostome divergence, which is why different scenarios have been proposed. Analysis of the somatic sea lamprey genome suggested that the most recent WGD (2R) is likely to have occurred before the divergence of the ancestral lamprey and gnathostome lineages (Smith et al., 2013). Other investigators suggested that lampreys may have experienced distinct polyploidization events from the gnathostomes and also may have had an additional independent WGD (Mehta et al., 2013). More recently, analyses of the sea lamprey germline genome supported two possible scenarios: (1) a single shared WGD or (2) two WGD followed by extensive gene losses from the resulting daughter chromosomes, especially in the lamprey (Smith and Keinath, 2015; Smith et al., 2018). One other study concluded that cyclostomes and gnathostomes have gone through the same two WGD events before they diverged from each other (Sacerdot et al., 2018). Others have proposed that only the first WGD was shared and was followed by independent duplication and loss events in the two lineages, a WGD in gnathostomes and unclear types of duplication in lampreys (Simakov et al., 2020).

Homologs of the gnathostome CRH family members have been reported for lampreys (Roberts et al., 2014; Cardoso et al., 2016; Endsin et al., 2017). The identification of lamprey peptides representing both of the two CRH/UCN subfamilies confirmed that these arose before the divergence of the cyclostome and gnathostome lineages (Cardoso et al., 2016). However, each of the lamprey CRH/UCN-sequences did not cluster clearly with each of the five gnathostome CRH-family sequences, thus it was not possible to assign orthology based upon sequence analysis. Also, as no information on synteny was available at the time, it was not possible to use this criterion to ascertain orthology between the lamprey and gnathostome members (Cardoso et al., 2016). Thus, it could not be inferred that cyclostomes and gnathostomes share the same two WGD events.

In this study, we investigated the early vertebrate evolution of the CRH family members and the implications for understanding the timing of the WGD events in relation to the agnathan-gnathostome divergence. We used a double comparative approach combining sequence analyses of available lamprey CRH-family genes and peptides with investigation of gene synteny for 37 neighboring gene families and their sequence-based phylogenies (and two hagfish CRH-family genes). Our data show that lampreys and gnathostomes have the same number of CRH family members in both of the peptide subfamilies. Furthermore, the lamprey genes are located in gene neighborhoods that resemble those that we have previously reported for gnathostomes, although some rearrangements have taken place. The most parsimonious explanation for these similarities is that lampreys and gnathostomes share five CRH orthologs that arose by chromosome duplications of two ancestral peptide genes. This would suggest that lampreys share the same genome doubling events as gnathostomes, albeit clouded by chromosomal recombination and changes in gene order along the chromosomes.

## Materials and Methods

### Identification of the Lampreys and Hagfish CRH-Family Genes

The mature predicted CRH-family members from our previous study (Cardoso et al., 2016), two from the sea lamprey (*Petromyzon marinus*) and four from the Arctic lamprey (*Lethenteron camtschaticum)*, were used to identify the missing genes and the scaffolds for all of the peptide genes in the sea lamprey and Arctic lamprey genome assemblies (available from NCBI database). The predicted mature peptides from lamprey were used to search for homologs in the inshore hagfish (*Eptatretus burgeri*) genome available from ENSEMBL. The identity of the CRH members that were retrieved was confirmed by submitting to the InterProScan tool^2^ or by sequence homology.

### Sequence Comparisons and Phylogeny

The complete deduced precursor sequences for both lamprey and hagfish CRH-family members were retrieved. Mature peptides were predicted by comparing with the gnathostome peptides and by localization in the sequence of putative proteolytic dibasic cleavage sites. Amino acid sequence identities were calculated using the Clustal Omega (Sievers et al., 2011), available from EMBL-EBI^3^.

Phylogenetic trees of the lamprey and hagfish CRH-family members with the other vertebrate homologs were constructed using both the complete peptide precursor sequences and the mature peptides. Sequences were aligned using the MUSCLE algorithm in the AliView platform 1.18 (Larsson, 2014) and trees were built according to the maximum likelihood (ML) and Bayesian inference (BI) methods. The alignment of the complete peptide precursors was manually edited to remove sequence gaps and poorly aligned regions. ML trees were calculated using the PhyML 3.0 algorithm ATGC bioinformatics platform with the SMS automatic model selection (Lefort et al., 2017) according to the AIC (Akaike Information Criterion). ML trees were constructed according to the LG substitution model (Le and Gascuel, 2008) and reliability of internal branching was accessed using 100 bootstrap replicates. The BI trees were constructed in the CIPRES Science Gateway (Miller et al., 2010) with MrBayes (Ronquist et al., 2012) run on XSEDE using the LG substitution model (Aamodel = LG) and 1,000,000 generation sampling and probability values to support tree branching. The tunicate (*Ciona intestinalis* and *Ciona savignyi*) CRH-like orthologs were used (Mirabeau and Joly, 2013). ML and BI trees were displayed with FigTree 1.4.2 and edited in Inkscape^4^.

### Gene Synteny Comparisons

The neighbors of the CRH family genes in lamprey and hagfish were identified and used to find orthologous genome regions in the spotted gar, chicken, and human. The gene environment of the sea lamprey scaffolds containing the CRH-family members (Supplementary Table S1) was annotated using a combination of the AUGUSTUS web interface (Stanke et al., 2004), by enquiring the species genome assembly at SIMRBASE database^5^ and the somatic genome assembly available from ENSEMBL^6^. We have annotated in detail 3 Mb of the sea lamprey scaffolds (1.5 Mb in each direction from the lamprey CRH-family gene loci, Supplementary Table S1). AUGUSTUS predicted complete and partial genes on both strands using the Arctic lamprey (*Lethenteron camtschaticum*) and human (*Homo* sapiens) as reference species. The gene environment of the Arctic lamprey homologous genome regions were predicted using a local installation of AUGUSTUS 2.5.5 (Stanke et al., 2004, 2008) with the settings set for sea lamprey to predict genes *de novo*. Gene identity was confirmed using Swissprot through BLAST2GO (Conesa et al., 2005) comparing to human, chicken, and spotted gar non-redundant protein (nr) databases. Searches for neighbors was complemented by procuring the species genome assemblies available from NCBI^7^. The neighboring genes of the hagfish CRH-like genome fragments were annotated using the BioMart tool available from ENSEMBL and compared with the spotted gar, chicken, and human, and common genes that were found were subsequently searched in the sea lamprey and Arctic lamprey genomes. The neighboring genes that we had previously identified (Cardoso et al., 2016) within the gnathostome CRH paralogons were also searched in lamprey and hagfish genomes.

To better comprehend the evolution of the lamprey and hagfish CRH members, phylogenetic analysis of their neighboring genes families was performed to investigate whether they had undergone similar evolutionary events. Orthologs of lamprey neighboring genes were retrieved from human, chicken, coelacanth, spotted gar, zebrafish, and elephant shark genomes available from ENSEMBL or NCBI. The invertebrate orthologs were retrieved from either two tunicates (*Ciona intestinalis* and/or *Ciona savignyi*), a cephalochordate (*Branchiostoma floridae*), or from the nematode (*Caenorhabditis elegans*) and fruit-fly (*Drosophila melanogaster*), and these were used to root the trees. Sequence alignments were performed using the AliView interface with MUSCLE, trees were carried out using the ML implemented in PhyML with automatic selection model, and sequence branching support was given by the Approximate Likelihood-Ratio Test (aLTR). The resulting trees were displayed in FigTree.

To deduce the putative ancestral pre-vertebrate CRH genomic region, we have used all the conserved cyclostome and gnathostome CRH-family neighboring genes to search for homologous genomic regions in invertebrate chordates where a CRH-like peptide gene has been described: two tunicates (*C. intestinalis* and *C. savignyi*) and two cephalochordates (*B. floridae* and *B. lanceolatum*).

## Results

### The Agnathan CRH-Family Members

Blast searches with the known CRH-family members identified five CRH-family sequences in both the sea lamprey and the Arctic lamprey genomes. These correspond to the five members described in our previous report (Cardoso et al., 2016), although we could not identify the complete set in both species at that time. No additional CRH-like sequences were identified. Thus, lampreys have the same number of CRH-family genes as the gnathostome ancestor and some extant gnathostomes. Analysis of the sea lamprey germline genome assembly revealed that the five CRH-family genes map to five different genome regions: scaffold_00040 (GL480439 in ENSEMBL), scaffold_82 (GL476347 in ENSEMBL), scaffold_00003, scaffold_00017, and scaffold_00057. The three latter genome scaffolds are absent from the sea lamprey somatic genome assembly (available from ENSEMBL). Similarly, the five Arctic lamprey CRH-family genes map to five distinct genome regions (KE993827, KE994103, KE993984, KE993813, KE993959). Searches in the hagfish genome assembly identified three putative CRH members that map to separate scaffolds (FYBX02010500.1, FYBX02010617.1, and FYBX02009844.1). However, analysis of the deduced CRH peptides encoded in scaffolds FYBX02010500.1 and FYBX02010617.1 revealed that they are 100% identical, thus we only considered FYBX02010500.1 for analysis. The lamprey CRH genes were designated CRH/UCN- a, b, and c and UCN-a and b (Table 1) according to phylogenetic clustering based on our previous and current analyses.

**TABLE 1:** Nomenclature adopted for the lamprey CRH-family genes.

	***P. marinus***	***L. camtschaticum***
CRH/UCN_a	scaf_00017	KE993984
CRH/UCN_b	scaf_00040	KE994103
CRH/UCN_c	scaf_00003	KE993827
UCN_a	scaf_00057	KE993813
UCN_b	scaf_00082	KE993959

The deduced amino acid sequences of the sea lamprey and Arctic lamprey genes were also aligned and compared with the three recently described sea lamprey cDNA sequences (Endsin et al., 2017) to confirm the genomic predictions and to correct for imprecisions in the automatic annotation. Orthologs between the two lamprey species had high sequence identity in their complete peptide precursors (>89% amino acid identity) or the corresponding deduced mature peptides (>98%), confirming the close evolutionary relationship of the two species (Table 2).

**TABLE 2:** Percent amino acid sequence identity of the lamprey CRH-family members.

	**Lca CRH/UCN-a**	**Pma CRH/UCN-b**	**Lca CRH/UCN-b**	**Pma CRH/UCN-c**	**Lca CRH/UCN-c**	**Pma UCN-a**	**Lca UCN_a**	**Pma UCN_b**	**Lca UCN_b**
Pma_CRH/UCN-a	**93 (100)**	33 (61)	31 (59)	39 (58)	36 (58)	19 (27)	18 (27)	20 (24)	21 (24)
Lca_CRH/UCN-a	100	33 (61)	32 (59)	38 (58)	37 (58)	19 (27)	20 (27)	20 (24)	21 (24)
Pma_CRH/UCN-b		100	**92 (98)**	30 (55)	27 (55)	22 (25)	21 (25)	17 (30)	19 (30)
Lca_CRH/UCN-b			100	29 (58)	28 (58)	19 (25)	19 (25)	14 (30)	16 (30)
Pma_CRH/UCN-c				100	**84 (100)**	26 (28)	27 (28)	19 (38)	20 (38)
Lca_CRH/UCN-c					100	25 (28)	27 (28)	19 (38)	19 (38)
Pma_UCN-a						100	**98 (100)**	33 (50)	32 (50)
Lca_UCN-a							100	33 (50)	33 (50)
Pma_UCN-b								100	**91 (98)**

**Complete deduced precursors and deduced mature peptides (within brackets). The percent sequence identity between the lamprey orthologs is in bold.**

The mature peptide sequences deduced in the lampreys were compared with the gnathostome CRH family peptides. However, orthologies based on sequence identity alone were unclear, presumably due to lineage-specific evolutionary pressures such as lamprey GC-rich DNA sequences leading to amino acids with GC-rich codons. Lamprey CRH/UCN-a deduced mature peptide sequence shares 85% aa identity with the human and spotted gar CRH1 peptides (Table 3). The lamprey CRH/UCN-b and CRH/UCN-c peptides also displayed highest sequence identity to the gnathostome CRH1 peptide (66 and 63% for human CRH1, respectively). Lamprey UCN-a shares the highest sequence identity with human UCN3 (61% aa), and with fish it has slightly higher identity to spotted gar UCN2 (68% aa) than UCN3 (65% aa). Lamprey UCN-b has highest identity to gnathostome UCN2 (55% aa for human and spotted gar).

**TABLE 3:** Percent amino acid identity of the deduced sea lamprey CRH-family peptides with human, spotted gar, and hagfish.

**Pma**	**Hsa CRH1**	**Loc CRH1**	**Loc CRH2**	**Hsa UCN1**	**Loc UCN1**	**Hsa UCN2**	**Loc UCN2**	**Hsa UCN3**	**Loc UCN3**	**Ebu CRH/UCN**	**Ebu UCN2/3**
CRH/UCN-a	**85**	**85**	61	45	49	37	29	32	28	**59**	27
CRH/UCN-b	**66**	**68**	49	43	51	32	25	29	25	51	23
CRH/UCN-c	**63**	**63**	55	45	43	32	25	29	25	55	23
UCN-a	29	27	33	23	20	45	**68**	**61**	65	24	**77**
UCN-b	29	29	23	38	27	**55**	**55**	50	48	29	44

**The highest sequence identities are highlighted in bold.**

The deduced hagfish CRH/UCN-peptide encoded in scaffold FYBX02010500.1 has 59% identity to lamprey CRH/UCN-a and the hagfish UCN2/3 peptide in scaffold FYBX02009844.1 has highest identity (77% aa) to lamprey UCN-a. The absence of additional hagfish CRH-like genes is probably due to incomplete genome assembly.

### Phylogenetic Analysis

**FIGURE 1:**
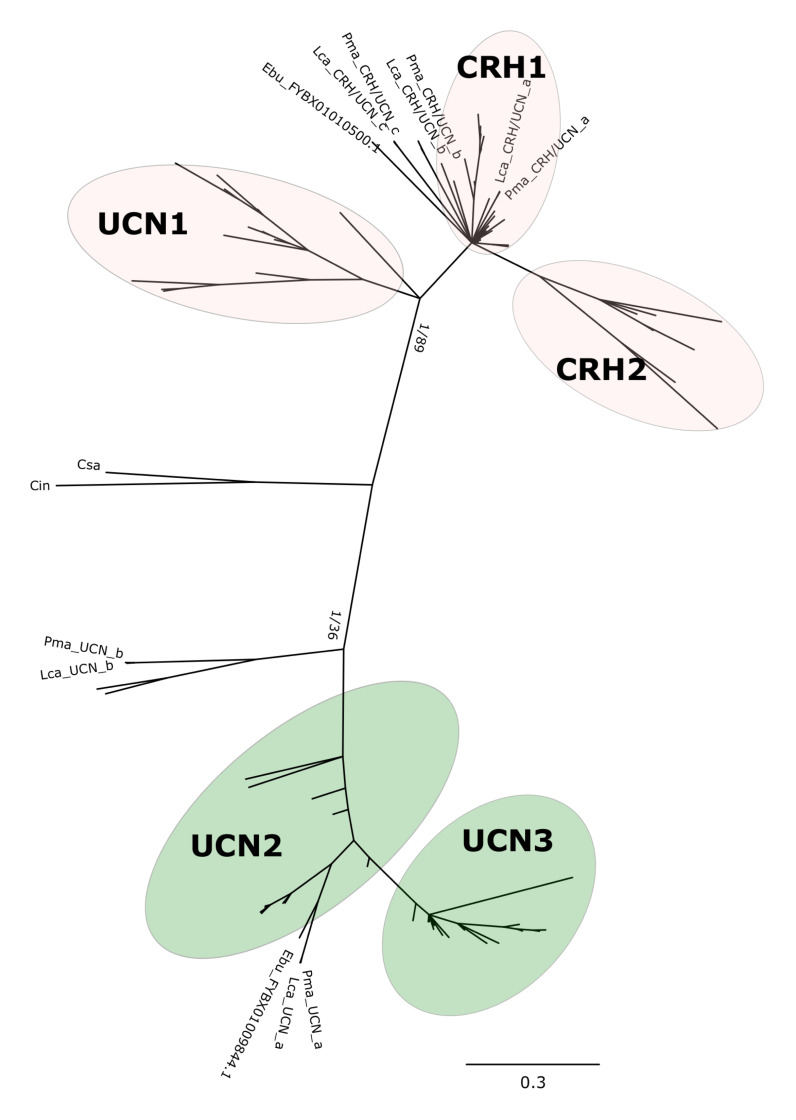
Edited radial phylogenetic tree of the chordate deduced mature peptides CRH-family members. Tree was constructed with the Bayesian inference (BI) and the complete tree is available in Supplementary Figure S1. The maximum likelihood (ML) tree is available in Supplementary Figure S3 and branch support values (BI posterior probability and ML bootstrap values) are shown only for the two major peptide subfamily clades. Accession numbers of the sequences used are available in Supplementary Table S2.

**FIGURE 2:**
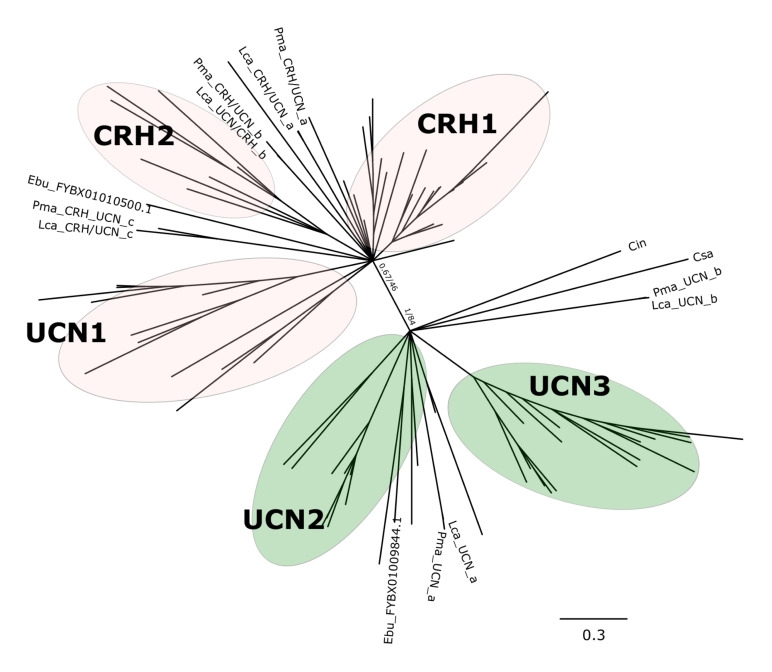
Edited radial phylogenetic tree of the chordate deduced full-length CRH-family precursors. Tree was constructed with the Bayesian inference (BI) and the complete tree is available in Supplementary Figure S2. The maximum likelihood (ML) tree is available in Supplementary Figure S4 and branch support values (BI posterior probability and ML bootstrap values) are shown only for the two major peptide subfamily clades. Accession numbers of the sequences used are available in Supplementary Table S2.

Phylogenetic analysis with the BI method of the agnathan and gnathostome deduced mature peptides (Figure 1 and Supplementary Figure S1) or the complete peptide precursors (Figure 2 and Supplementary Figure S2) produced tree topologies that are in agreement with our previous study (Cardoso et al., 2016). Phylogenetic trees with the ML method generated essentially identical topologies (Supplementary Figures S3, S4). All trees positioned the agnathan genes within either of the two CRH/UCN subfamilies (see Figures 1, 2). Three of the lamprey CRH-members grouped in the gnathostome CRH1/CRH2/UCN1 subfamily and the two others within the gnathostome UCN2/UCN3 subfamily (Figure 1 and Supplementary Figures S1, S3; Figure 2 and Supplementary Figures S2, S4). The two hagfish sequences were separated into each of the two subfamilies.

The five peptides from the two lampreys form five closely clustered pairs reflecting orthology between the species. One hagfish sequence clusters closest to the lamprey UCN_a sequences. The agnathan sequences do not cluster with clear bootstrap support with each of the five gnathostome peptide clades. The agnathan mature peptides and precursor sequences generally tended to radiate earlier and at the base of the discrete gnathostome peptide clades.

### Features of CRH-Family Peptides

**FIGURE 3:**
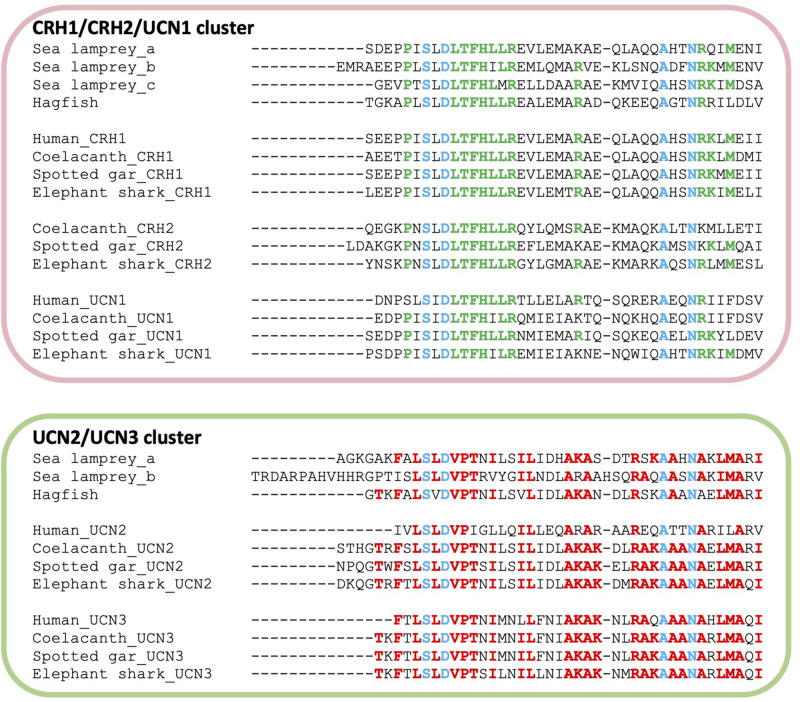
Mature peptide sequence alignment of the lamprey, hagfish, and gnathostome CRH-family members. Peptides were predicted by comparing with the gnathostome peptides and by localization in the sequence of putative proteolytic dibasic cleavage sites. Amino acids conserved in all peptides are shown in blue. Positions shown in green or red are amino acids conserved within the CRH1/CRH2/UCN1 and UCN2/UCN3 subfamilies, respectively.

The deduced mature peptide sequences of the cyclostome CRH-members were aligned and compared with the gnathostome orthologs according to clustering obtained from the phylogenetic tree (Figure 3). Amino acid residues and motifs that are common between the lamprey, hagfish, elephant shark, spotted gar, coelacanth, and human CRH-family members were found (Figure 3 in blue), including those that are characteristics of the CRH1/CRH2/UCN1 (Figure 3 in green) and UCN2/UCN3 (Figure 3 in red) clades.

The S-L-D motif that is conserved at the N-terminus of the gnathostome CRH-members (except in the UCN clade) is also conserved in all agnathan peptides, including the asparagine (R) residue that is localized near the C-terminus and the alanine (A) residue located three positions before. Motifs characteristic of the CRH1/CRH2/UCN1 subfamily (Figure 3, in green) and UCN2/UCN3 subfamily (Figure 3, in red) are also maintained in the lampreys and hagfish. This includes the gnathostome LTFH(L/I)LR localized in the mid-region of CRH1, CRH2, and UCN1, and other amino acid residues (Figure 3, in green). Within the UCN2/UCN3 peptide alignment, the cyclostome peptides share the VPT motif as well as additional residues with the gnathostome UCN2 (Figure 3, in red).

### Gene Structure

**FIGURE 4:**
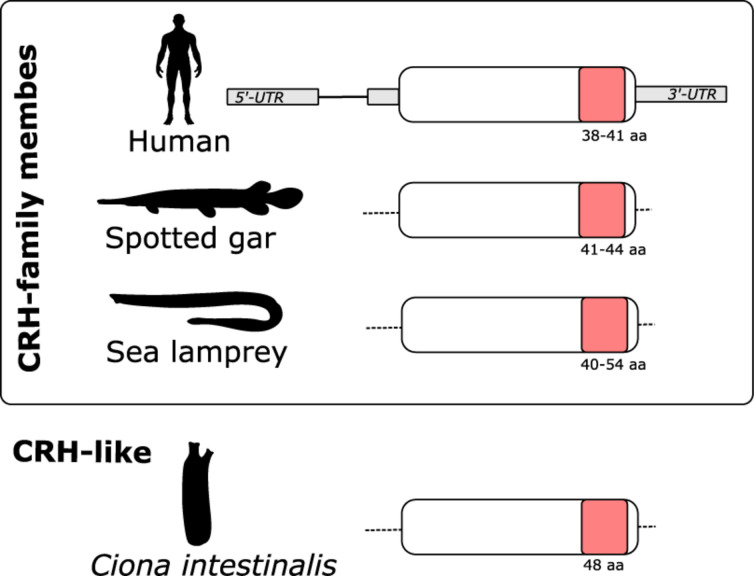
Schematic representation of the gene structure of CRH-family members. In both gnathostomes and lamprey the CRH-family members are encoded by a single exon. In human the CRH-family gene structure is composed by 2 exons: exon 1 contains the 5′ UTR and exon 2 encoded the CRH-family member peptide precursors (represented in white) that contains the mature peptide (represented in pink) and the 3′UTR (represented in gray). In the spotted gar and lamprey, the complete gene structures of the CRH-family members have not yet been elucidated. The predicted sizes (aa) of the mature CRH-family peptides are indicated. Figure is not drawn to scale.

The gnathostome CRH-family genes are composed of two exons, the second of which encodes the entire peptide precursor (Shibahara et al., 1983; Thompson et al., 1987). Characterization of the lamprey (Figure 4) and hagfish genes revealed that they too encode the entire prepro-peptide on a single exon. This is the situation also for the Ciona CRH-like gene (Figure 4).

### Neighboring Gene Family Analysis

**FIGURE 5:**
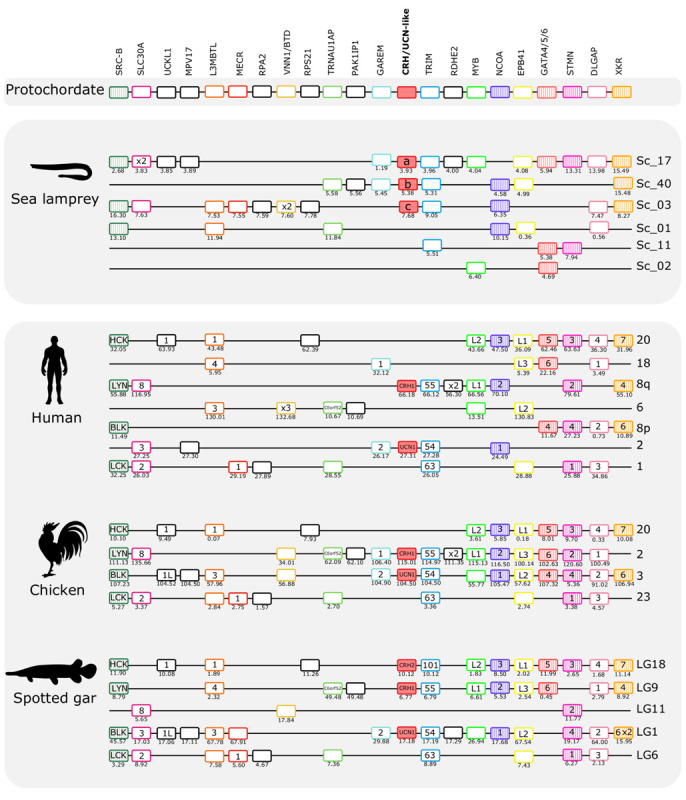
Gene synteny analysis of the opioid (CRH1/CRH2/UCN1) paralogon in lamprey and gnathostomes. Chromosomal locations of the CRH-family members and of 21 neighboring genes families in lamprey, human, chicken, and spotted gar are shown. Chromosomes or scaffold numbers as well as gene positions (Mb, below each gene) are given. Genes are represented by boxes and CRH/UCN members are represented by full-red boxes and neighboring gene families are represented by different colors. The gene family symbol is shown, and the designation of the different members is provided inside the corresponding gene. Gene duplicates of the same family that map to the same genome region are represented (x2 and x3). The genes in the lamprey scaffolds are displayed according to their order in the genome region analyzed but in other species the genes were reshuffled to highlight the similarities between species. The neighboring gene families that we have previously described in the gnathostome CRH1/CRH2/UCN1 paralogon are also represented and are shown as striped colored boxes (Cardoso et al., 2016). The accession numbers and phylogenetic trees of the gene families represented are available in Supplementary Table S3 and Supplementary Figure S5.

**FIGURE 6:**
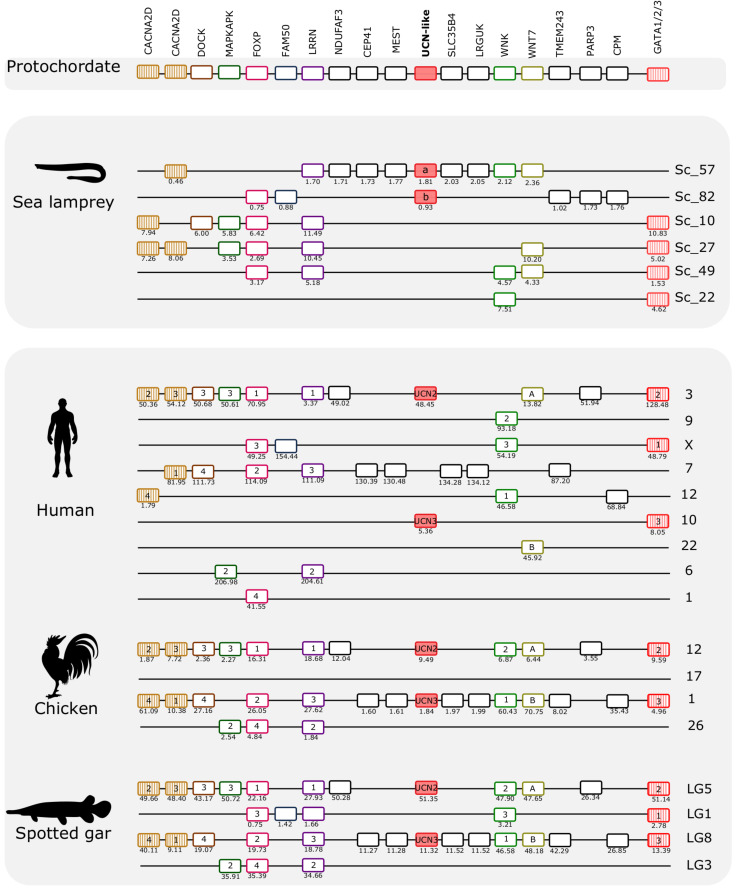
Gene synteny analysis of the opsin (UCN2/UCN3) paralogon in lamprey and gnathostomes. Chromosomal locations of the CRH-family members and of 17 neighboring gene families in the sea lamprey, human, chicken, and spotted gar are shown. The chromosome or scaffold numbers as well as gene positions (Mb, below each gene) are given. Genes are represented by boxes and UCN members are represented by full-red boxes and neighboring gene families are represented by different colors. The gene family symbol is shown, and the designation of the different members is provided inside the corresponding gene. The genes in the lamprey scaffolds are represented according to their order in the genome fragment analyzed but in other species were reshuffled to highlight the similarities between species. The genes that we have previously identified in UCN2/UCN3 paralogon are represented by stripped colored boxes (Cardoso et al., 2016). The accession numbers and phylogenetic trees of the gene families represented are available in Supplementary Table S4 and Supplementary Figure S6.

The gnathostome CRH family genes map to two distinct paralogons: The CRH1/CRH2/UCN1 genes are located on separate chromosomes in the paralogon that also contains the genes for the opioid receptors (Dreborg et al., 2008) and the opioid peptides (Sundström et al., 2010), and the UCN2/UCN3 genes are found in a paralogon that contains the visual opsin genes (Lagman et al., 2013). Mapping of the lamprey CRH-family chromosomal regions shows that they possess similar gene repertoires to those in human, chicken, and spotted gar (Figures 5, 6). This strongly suggests that agnathan and gnathostome CRH family genes most likely shared the same ancestral gene neighborhood, and that many of these genes have remained neighbors by contingency. Furthermore, the comparisons show that many of the neighboring genes belong to families consisting of quartets, triplets, or pairs, with members nearby the different CRH-family members. This is consistent with duplication of large chromosomal blocks or regions containing a large number of genes. Also, many neighboring genes that remain singletons (i.e., no duplicates have survived the chromosome duplications) support a common ancestry for these chromosomal regions in living agnathans and gnathostomes.

The conservation of synteny does not seem to extend to tunicates; despite the existence of a CRH-like peptide in Ciona, no clear homologous genomic region composed by similar genes to those of the vertebrate CRH family members was found. Likewise, no such conserved gene synteny region could be identified in the cephalochordate genome.

### The CRH1/CRH2/UCN1 (Opioid) Paralogon

**FIGURE 7:**
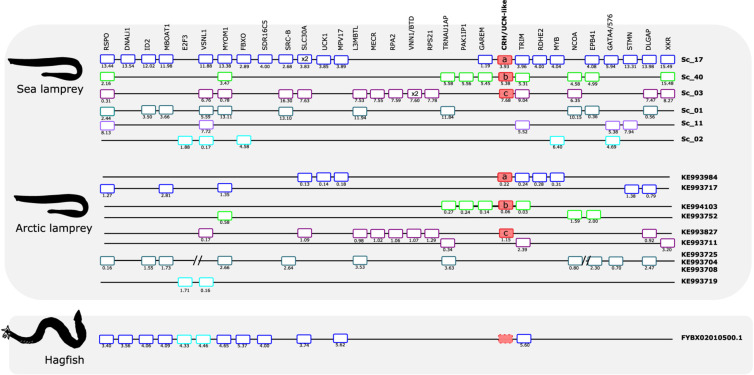
Gene synteny of the opioid (CRH/UCN) paralogon in the cyclostomes. The sea lamprey, Arctic lamprey, and hagfish homolog genome regions were compared. To highlight for potential gene shuffling/crossing-over events between the two lampreys and the two lampreys and hagfish, the sea lamprey genes that map in the same scaffold were represented by the same color. The exceptions are the CRH/UCN members which are represented by red-full boxes. Members of the same family in the sea lamprey genome or homologs in the other two cyclostome species are aligned. Gene chromosomal positions (Mb, below each gene) are shown. The sea lamprey genes are represented according to their position in the scaffolds and homologs in the Arctic lamprey and hagfish were positioned in relation to the sea lamprey genes. When possible different fragments of Arctic lamprey genome that possess a similar gene environment to a unique sea lamprey scaffold region were merged and this is marked by “//.” The hagfish CRH-like gene is dashed because this gene is not predicted in the species genome assembly. Tandem duplicate genes are represented by x2. Homologs for other lamprey CRH/UCN-like neighboring genes were found in the hagfish genome assembly but are not shown as they are located on multiple small scaffolds.

The three sea lamprey CRH-family members were found in scaffolds 3, 17, and 40, respectively. A total of 21 neighboring gene families that are syntenic with the gnathostome CRH1/CRH2/UCN1 paralogon were identified and characterized (Figure 5). Six of these gene families also have members in a fourth sea lamprey chromosomal region contained in scaffold 1, and a few additional genes are contributed by scaffolds 2 and 11. Six of the neighboring gene families in spotted gar are quartets, namely DLGAP, EPB41, L3MBTL, SRC-B, STMN, and TRIM. Four of the neighboring families in spotted gar are triplets, i.e., SLC30A, MYB, NCOA, and XKR (Figure 5). In the sea lamprey, one family is a quartet (TRIM) and six families consist of triplets. This is slightly fewer than the spotted gar, so either lineage-specific gene losses occurred in the sea lamprey (or cyclostomes) lineage, or the sea lamprey genome assembly is not quite complete. Taken together, the sea lamprey CRH1/CRH2/UCN1 subfamily genes and neighboring gene families comprise a paralogon with fourfold symmetry consistent with four related chromosomal regions, i.e., two doubling events, as in gnathostomes. Two gene families in the sea lamprey, RSPO and VSNL1, even consist of five members (Figure 7), too many to be perfectly consistent with a chromosome quadruplication scenario, but the extra fifth gene could have arisen in an independent duplication event in the same time window as the genome doublings. A number of singletons that flank the CRH-family genes in lamprey have homologs that also occur as singletons in the gnathostome genomes.

The comparison with the Arctic lamprey (Figure 7) confirms much of the sea lamprey gene repertoire and organization, though it lacks some of the genes identified in sea lamprey. Only a few genes that are present in the Arctic lamprey are missing in the sea lamprey, suggesting that the latter is more complete. Orthologs in the two lamprey species are highly identical in sequence.

The hagfish genome has only a single member of the CRH1/CRH2/UCN1 subfamily (Figure 7). This scaffold shares three neighboring gene family members with the gnathostome species shown in Figure 5, namely SLC30A, TRIM, and MPV17, and shares 12 neighbors with the scaffolds in the two lamprey species. However, it is difficult to say exactly to which of the lamprey regions it is orthologous as the hagfish scaffold’s gene repertoire appears to be a hybrid between sea lamprey scaffolds 2 and 17, suggesting that gene shuffling occurred after separation of the two agnathan lineages. Homologs for other lamprey CRH-like neighboring genes were also found in hagfish genome, but they are located on multiple small scaffolds and do not provide synteny evidence.

Phylogenetic analyses were carried out for all of the 21 neighboring gene families and revealed a similar topology as for the CRH-family genes, i.e., the duplications seem to have taken place in the time range of early vertebrate evolution (Supplementary Figure S5). Occasionally, some family members display somewhat deviating species divergences compared to the established species phylogeny, especially sequences from the slowly evolving lineages represented by coelacanth, spotted gar, and elephant shark. For instance, the coelacanth sequence may cluster with the actinopterygian representatives rather than the sarcopterygian species, as in the LYN tree in Supplementary Figure S5. In the tree for the SLC30A family, the member SLC30A8 has coelacanth and elephant shark as closest relatives. Such slight variations in clustering are not unusual in evolutionary analyses of vertebrates due to variable evolutionary rates for the lineages. For some gene families, some members or species display a more dramatic difference in evolutionary rate, such as the human SLC30A3 gene. Nevertheless, each gene family member usually displays high statistical support for the clade comprised by the orthologs from the species included in this analysis.

The lamprey sequences vary more in their positions in the phylogenetic trees. Sometimes they branch off basally to the gnathostome members. Also, the different gene family members from lampreys may group together as if they had been duplicated in this lineage, although the synteny and paralogon analyses support simultaneous duplication along with the gene neighbors in large chromosomal blocks or entire chromosomes.

### The UCN2/UCN3 (Opsin) Paralogon

The two sea lamprey UCN2/UCN3-like genes map to scaffolds 57 and 82, respectively. Genes that belong to this paralogon were also found in four other scaffolds (Figure 6). A total of 17 neighboring gene families were identified and characterized that are in synteny with this paralogon in gnathostomes. Detailed analyses of these genomic regions identified two gene families that are quartets in spotted gar, and two families that are triplets. Several pairs were identified. Interestingly, both of the gene families that are quartets in spotted gar are so also in the sea lamprey genome, namely the FOXP family and the LRRN family. One of the spotted gar triplets, WNK, is likewise a triplet in sea lamprey. The other spotted gar triplet, GATA1/2/3, has a more complex situation in the sea lamprey with as many as six members, one of which might represent an ancestral fourth member in a 2R quartet (these four are shown in Figure 6), whereas two have arisen by duplication in the lamprey lineage (see tree in Supplementary File 5).

Considering that there are also several gene pairs present in these chromosomal regions in the various vertebrate genomes, a picture emerges of fourfold symmetry, albeit with weak representation of the fourth member in this paralogon. Overall, these observations point to a paralogon with four members, as with the CRH paralogon, thus most likely reflecting similar genomic events. Like in the other paralogon, many neighboring gene families remain as singletons both in lamprey and in gnathostomes.

**FIGURE 8:**
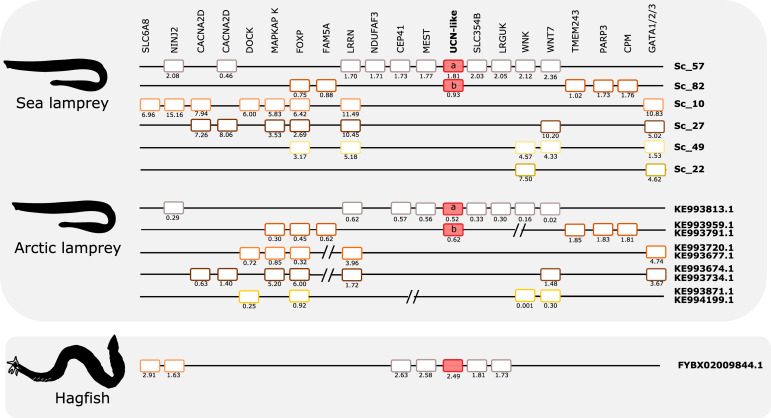
Gene synteny of the opsin (UCN) paralogon in cyclostomes. The homolog genome region in sea lamprey, Arctic lamprey, and hagfish were compared. To highlight for potential gene shuffling/crossing-over events between the two lampreys and the two lampreys and hagfish, the sea lamprey genes in the same scaffold were represented by the same color. The exceptions are the UCN members which are represented by red-full boxes. Members of the same family in the sea lamprey genome or homologs in the other two cyclostome species are aligned. Gene chromosomal positions (Mb, below each gene) are shown. The sea lamprey genes are represented according to their position in the scaffolds and homologs in the Arctic lamprey and hagfish were positioned in relation to the sea lamprey genes. When possible different fragments of Arctic lamprey genome that possess a similar gene environment to a unique sea lamprey scaffold region were merged and this is marked by “//.” Homologs for other lamprey UCN-like neighboring genes were found in the hagfish genome assembly but are not represented as they are located on multiple small scaffolds.

Comparison of the two lamprey species reveals high similarity of not only gene repertoire, but also gene order (Figure 8). The hagfish scaffold with the UCN2/UCN3-like gene shares with the lampreys close synteny of four genes (CEP41, MEST, SLC35/4B, and LRGUK). Two other genes that are present in the hagfish scaffold (SLC6A8 and NINJ2) have lamprey orthologs that are syntenic with a different member of this paralogon, suggesting that gene translocation occurred after divergence of hagfishes and lampreys.

Phylogenetic analyses of all of the neighboring gene families overall display a similar topology as for the CRH/UCN genes. All of the multimember families display gene duplications in the time range of the early vertebrates WGD events (Supplementary Figure S6). Some of the neighboring gene families in this paralogon display similar features to those in the opioid paralogon described above, such as uneven evolutionary rates for some gene family members and variable branch positions for the cyclostome sequences.

## Discussion

Our previous analyses of the CRH family allowed us to conclude that five genes arose before the gnathostome radiation as a result of the two WGD events, which triplicated one ancestral gene and duplicated the other (Cardoso et al., 2016). We also reported that a total of five CRH-family genes exist in lampreys as a group (Cardoso et al., 2016). However, in the sequence-based phylogenetic trees, the lamprey peptides did not cluster clearly with each of the five gnathostome peptides, and it was not possible to assign orthology. Thus, we could not determine whether lampreys share the same two WGD events as the gnathostomes. The recent genome assemblies of the sea lamprey *Petromyzon marinus* and the Arctic lamprey *Lethenteron camtschaticum*, and the hagfish *Eptatretus burgerii*, have allowed us to analyze scaffolds containing several neighboring genes to shed light on the agnathan-gnathostome divergence in relation to the two WGD events.

### Five CRH-Family Genes in Lampreys

Five CRH-family genes were found in both of the lamprey species, confirming our previous conclusion (Cardoso et al., 2016) and showing that both species share the same complete set. Two genes were found in the hagfish. In gnathostomes the mature peptides are encoded by a single exon, and this is the case also for the lampreys and the hagfish. Sequence-based phylogenetic analyses show that three lamprey peptides and one hagfish peptide cluster with the gnathostome CRH1/CRH2/UCN1 subfamily, and two lamprey peptides and one hagfish peptide cluster with the UCN2/UCN3 subfamily (Figures 1, 2). This clearly demonstrates that the duplication that gave rise to the two ancestral CRH genes, the founders of each of the two subfamilies, had taken place well before the agnathan-gnathostome divergence. However, it was still not possible to determine orthology within each subfamily from the sequence analyses alone. Comparison of each of the five peptide genes between sea lamprey and Arctic lamprey showed high identity, consistent with recent divergence of the two species 10–30 Mya (Kuraku and Kuratani, 2006).

One of the two identified hagfish peptides clustered most closely to lamprey UCN_a (Figures 1, 2), with which it displays gene environment conservation (Figure 8) and this is consistent with the hagfish lineage having undergone similar evolutionary events as the lamprey lineage (Fujimoto et al., 2013). Regarding orthology to the gnathostome sequences, this was as difficult to assign as for the lamprey sequences. This situation prevailed regardless whether the analysis was performed for the mature peptides (Figure 1) or the complete peptide precursors (Figure 2).

One reason that it is difficult to establish orthology between lamprey and gnathostome gene or protein family members may be that lamprey genes are under selection pressure to have high GC content, especially in coding regions (Kuraku and Kuratani, 2006; Kuraku, 2008; Smith et al., 2013; Manousaki et al., 2016). This probably explains the tendency of lamprey protein family members to cluster with one another in phylogenetic analyses rather than with their gnathostome orthologs (Qiu et al., 2011; Wotton and Shimeld, 2011; Fujimoto et al., 2013; Nah et al., 2014), as observed in the present report for both the CRH-family sequences and some of the neighboring gene families. Preference for certain synonymous codons occurs in bacteria, plants and invertebrates (Grantham et al., 1980; Qin et al., 2004), but in vertebrates it has been suggested to be gene specific and to occur at different rates across species (Palidwor et al., 2010; Romiguier et al., 2010; Aoi and Rourke, 2011). In contrast, lampreys seem to have a more general selection for GC-rich codons (Smith et al., 2013; Manousaki et al., 2016).

### Lamprey CRH-Family Genome Regions

As an additional approach to distinguish orthologs and paralogs, we and others have analyzed the repertoire of neighboring gene families in numerous studies. We therefore analyzed the CRH neighbors and their families in the scaffolds containing the lamprey and hagfish CRH-family genes. As shown in Figures 5, 6, all of these agnathan scaffolds contain members of gene families that are located close to the gnathostome CRH-family genes. In total, the scaffolds covering the CRH-family gene neighborhood contain a total of 40 gene families, including the two CRH subfamilies and the two GATA subfamilies.

An important result of our analyses of the lamprey scaffolds is that some of the neighboring gene families are quartets: one in the CRH/opioid paralogon (TRIM) and two in the UCN/opsin paralogon (FOXP and LRRN). In addition, several neighboring families are triplets, six in the CRH/opioid paralogon and two in the UCN/opsin paralogon. Two of the lamprey families, RSPO and VSNL1 (Figure 7), actually consist of five members, and their phylogenetic trees suggest that all duplications took place in the time period of the cyclostome-gnathostome divergence (not shown). One of the UCN/opsin triplets also includes a few additional members, namely the GATA1/2/3 family, but the supernumerary members seem to be lamprey-specific duplicates (see tree in Supplementary File 5). A few such lineage-specific duplications have occurred also in the human and chicken lineages (Figures 5, 6).

Counting also the CRH1/CRH2/UCN1 family, this means that in total 13 out of 37 gene families in these two sets of scaffolds contain gene families consisting of three or four (or five) members in lampreys. In addition, pairs of related genes are present in different combinations of these chromosomal regions, further corroborating relatedness. The most parsimonious explanation for this would be a quartet of related chromosomal regions. By parsimonious reasoning, the simplest explanation would be that lampreys have undergone the same two WGD events as gnathostomes, thus sharing the same 1R and 2R genome doublings. The number of gene families with quartets and triplets of genes is not quite as high as in spotted gar, suggesting that lampreys have lost some family members, as seems to be the case for human and chicken.

Assuming that lampreys have undergone the same duplication events as gnathostomes for the CRH-containing chromosome regions, it might theoretically be possible to infer which of the chromosome members in the lamprey paralogon that corresponds to which member in the gnathostomes, i.e., to assign orthology between these basal vertebrate lineages. Also, the pattern of neighboring singletons or gene losses should allow identification of orthologous chromosomal regions between lamprey and gnathostomes. However, careful scrutiny of the scaffolds shows that some lamprey regions appear to be combinations of two (or more) gnathostome regions. For instance, in the UCN/opsin paralogon, there are two UCN genes in the lamprey that might correspond to spotted gar linkage groups LG5 and LG8, both of which have rather complete representation of the neighboring gene families. One of these could correspond to lamprey scaffold 57 and the other to scaffold 82. However, when looking at the gene families that are quartets, namely FOXP and LRRN, neither of these two scaffolds can be complemented by either of the scaffolds 10, 27, or 49, because all three of these contain representatives for both of these quartet families. There are a number of such examples where combination of chromosomes in either lamprey or one of the vertebrates leads to “collision,” as has also been observed for the somatostatin gene regions in *L. camtschaticum* (a.k.a. *L. japonicum*) (Tostivint et al., 2016). One possible explanation for this is that either the lamprey or the gnathostome ancestor’s chromosome underwent crossing-over, presumably rather soon after WGD when the duplicated chromosomes were still quite similar to one another also in intergenic regions. Alternately, patterns such as this might simply reflect independent paralog loss, fission, and local duplication events in lamprey vs. gnathostome genomes.

Recently, three CRH-family members were amplified from the sea lamprey brain cDNA (Endsin et al., 2017). The three peptides were suggested to be the homologs of gnathostome CRH1, UCN1, and UCN3. However, our more extensive analyses based on both phylogeny and gene synteny suggest that this may not be the case. These authors proposed that the three lamprey sequences arose by two duplication events before the cyclostome-gnathostome divergence and that two subsequent duplication events expanded the repertoire to five genes in the gnathostome lineage (Endsin et al., 2017), although our previous study had already demonstrated five CRH-family members in lampreys (Cardoso et al., 2016). Our results presented here add further support that all of the duplications of the ancestral CRH-bearing region took place before the lamprey-gnathostome divergence.

Our results clearly establish that the two CRH-family lineages separated before the lamprey-gnathostome divergence. The peptide sequences in both lamprey and hagfish show that the CRH1/CRH2/UCN1 ancestor and the UCN2/UCN3 ancestor arose before the origin of the vertebrates. These two ancestral genes, the founders of the two subfamilies, became located in separate chromosomes that subsequently formed the two paralogons in the 2R events. Interestingly, one other gene family has members in the same two paralogons: the GATA family of transcription factors. Our previous analysis (Cardoso et al., 2016) showed that GATA1/2/3 family members are located in the UCN/opsin paralogon and that GATA4/5/6 are in the CRH/opioid paralogon, as had been reported previously (Hwang et al., 2013). Both of the GATA lineages have been identified in the nematode *C. elegans*, in several arthropods including the fruit-fly *D. melanogaster*, and also in annelids, showing that the first gene duplication occurred prior to the protostome-deuterostome divergence (Gillis et al., 2007, 2008) and probably much earlier. In protostomes, homologs of vertebrate CRH have only been described in insects (DH44 peptide). We therefore wondered whether CRH and GATA were duplicated in the same event at the same time point. However, only a single gene homolog of vertebrate CRH exists in invertebrate genomes (Mirabeau and Joly, 2013), suggesting that GATA and CRH were duplicated independently and maybe ended up in the same two gene regions by chance.

### Lampreys and Gnathostomes Probably Share the Same WGD Events

Our analyses of the gnathostome CRH chromosomal regions and homologous regions in sea lamprey and Arctic lamprey suggest that lampreys share not only the first WGD event (1R) with gnathostomes but also the second WGD event. Some previous analyses of the sea lamprey genome described gene family relationships and conserved syntenic regions consistent with a shared 2R event (Caputo Barucchi et al., 2013; Decatur et al., 2013; Smith et al., 2018). However, analyses of the Hox gene clusters of the Arctic lamprey genome led to the suggestion that lamprey genomes might have undergone a third genome doubling and that 2R and perhaps even 1R could have been independent events from those in gnathostomes (Mehta et al., 2013; Zhang et al., 2017). A more recent analysis of vertebrate chromosome evolution concluded that cyclostomes share the 2R event with gnathostomes, see figure 7 in Sacerdot et al. (2018). However, other authors settled for a shared 1R event and suggested that lampreys subsequently have undergone “one or more additional duplication(s)” (Simakov et al., 2020). Our characterization of the lamprey CRH-family members and their neighboring gene environment reveals striking similarity to the chromosome regions in gnathostomes, which appears to be in line with both WGD events being shared with gnathostomes. However, it remains theoretically possible that lampreys have had independent WGD (or other duplication) events as long as the sequence-based phylogenies cannot be completely resolved for the cyclostome-gnathostome divergence in relation to the genome doublings, as has been proposed for the Hox clusters (Smith et al., 2018).

## Conclusion

Homologs of the five gnathostome CRH-family members and their neighboring gene families were characterized in the genomes of two lampreys and one hagfish. In lampreys, like in gnathostomes, five members of the CRH family were identified, presumably representing orthologs. However, the exact orthologous relationships could not be resolved even after synteny and paralogon analyses, presumably due to crossing-over events or other rearrangements that have changed their chromosomal positions. Although orthology between lampreys and gnathostomes cannot be determined with certainty, our detailed analyses nevertheless find strong support for lamprey chromosome quadruplication in two paralogons, and thus that lampreys (and by inference also the hagfishes if these two lineages of agnathans are monophyletic) have undergone duplications that are highly similar to those experienced by gnathostomes. Most parsimoniously, these duplication events were probably the same as in the gnathostomes, i.e., WGD events, and the lamprey-gnathostome divergence took place soon after the second WGD event.

## Data Availability Statement

The accession numbers of all sequences used in this study were retrieved from public databases and their accession numbers are available in Supplementary Material.

## Author Contributions

JC, CB, and DL planned the study, evaluated the results, and wrote the manuscript. JC and CB collected the data and performed the analyses. All authors critically read and contributed to improve the manuscript.

## Conflict of Interest

The authors declare that the research was conducted in the absence of any commercial or financial relationships that could be construed as a potential conflict of interest.

## Footnotes

1 www.ensembl.org

2 https://www.ebi.ac.uk/interpro/search/sequence-search

3 https://www.ebi.ac.uk

4 https://inkscape.org

5 https://genomes.stowers.org/

6 http://www.ensembl.org/

7 https://www.ncbi.nlm.nih.gov/assembly/

## Supplementary Material

FIGURE S1 Bayesian inference tree of the CRH mature peptides. Branch support values (posterior probability values) are shown and tree was rooted with the tunicate CRH-like precursors. Tree was rooted with the tunicate CRH-family members. A simplified radial tree is represented in Figure 1. Accession numbers of the sequences used are available in Supplementary Table S2.

FIGURE S2 Bayesian inference tree of the CRH- peptide precursors. Branch support values (posterior probability values) are shown and tree was rooted with the tunicate CRH-like precursors. Tree was rooted with the tunicate CRH-family members. A simplified radial tree is represented in Figure 2. Accession numbers of the sequences used are available in Supplementary Table S2.

FIGURE S3 Maximum likelihood tree of the CRH mature peptides. Tree was constructed using the chordate deduced full-length CRH-family precursors with the PhyML 3.0 algorithm on the ATGC bioinformatics platform using LG substitution and 100 bootstrap replicates. Tree was rooted with the tunicate CRH-family members. Accession numbers of the sequences used are available in Supplementary Table S2.

FIGURE S4 Maximum likelihood tree of the CRH peptide precursors. Tree was constructed with the chordate deduced full-length CRH-family precursors using the PhyML 3.0 algorithm on the ATGC bioinformatics platform using LG substitution and 100 bootstrap replicates. Tree was rooted with the tunicate CRH-family members. Accession numbers of the sequences used are available in Supplementary Table S2.

FIGURE S5 Phylogenetic analysis of the opioid (CRH1/CRH2/UCN1) paralogon neighboring gene families. Trees were constructed with the PhyML 3.0 algorithm on the ATGC bioinformatics platform with automatic selection model and sequence branching support was given by the Approximate Likelihood-Ratio Test (aLTR). All neighboring gene trees were rooted using the invertebrate family members. The accession numbers and symbols of all the sequences used are available in Supplementary Table S3.

FIGURE S6 Phylogenetic analysis of the opsin (UCN2/UCN3) paralogon neighboring gene families. Trees were constructed with the PhyML 3.0 algorithm on the ATGC bioinformatics platform with automatic selection model and sequence branching support was given by the Approximate Likelihood-Ratio Test (aLTR). All neighboring gene trees were rooted using the invertebrate family members. The accession numbers and symbols of all the sequences used are available in Supplementary Table S4.

TABLE S1 Size (base pairs, bp) of the sea lamprey and Arctic lamprey CRH-family scaffolds. The analyzed genome regions of the sea lamprey scaffolds were selected using as starting point the position of the lamprey CRH-family member and retrieving approximately 1.5 Kb upstream and downstream of this gene.

TABLE S2 Accession numbers of all the CRH-family members used in phylogeny. The genome localization (chromosome and position) is give. ni, not identified; na, not available; un, uncharacterized.

TABLE S3 Accession numbers (ENSEMBL or NCBI) and nomenclature adopted for the neighboring gene families of the CRH1/CRH2/UCN1 paralogon that were used for phylogenetic analysis (Supplementary Figure S5). Gene families were ordered as they appear in Figure 5.

TABLE S4 Accession numbers (ENSEMBL or NCBI) and nomenclature adopted for the neighboring gene families of the UCN2/UCN3 paralogon that were used for phylogenetic analysis (Supplementary Figure S6). Gene families were ordered as they appear in Figure 6.

## References

[B1] Aoi M. C. Rourke B. C. (2011). Interspecific and intragenic differences in codon usage bias among vertebrate myosin heavy-chain genes. *J. Mol. Evol.* 73 74–93. 10.1007/s00239-011-9457-0 21915654

[B2] Audsley N. Kay I. Hayes T. K. Coast G. M. (1995). Cross reactivity studies of CRF-related peptides on insect Malpighian tubules. *Comp. Biochem. Physiol. Part A Physiol.* 110 87–93. 10.1016/0300-9629(94)00132-D 7866779

[B3] Cabrero P. Radford J. C. Broderick K. E. Costes L. Veenstra J. A. Spana E. P. (2002). The Dh gene of *Drosophila melanogaster* encodes a diuretic peptide that acts through cyclic AMP. *J. Exp. Biol.* 205 3799–3807. 12432004 10.1242/jeb.205.24.3799

[B4] Caputo Barucchi V. Giovannotti M. Nisi Cerioni P. Splendiani A. (2013). Genome duplication in early vertebrates: insights from agnathan cytogenetics. *Cytogenet. Genome Res.* 141 80–89. 10.1159/000354098 23949002

[B5] Cardoso J. C. R. Bergqvist C. A. Félix R. C. Larhammar D. (2016). Corticotropin-releasing hormone family evolution: five ancestral genes remain in some lineages. *J. Mol. Endocrinol.* 57 73–86. 10.1530/JME-16-0051 27220618

[B6] Conesa A. Götz S. García-Gómez J. M. Terol J. Talón M. Robles M. (2005). Blast2GO: a universal tool for annotation, visualization and analysis in functional genomics research. *Bioinformatics* 21 3674–3676. 10.1093/bioinformatics/bti610 16081474

[B7] Decatur W. A. Hall J. A. Smith J. J. Li W. Sower S. A. (2013). Insight from the lamprey genome: glimpsing early vertebrate development via neuroendocrine-associated genes and shared synteny of gonadotropin-releasing hormone (GnRH). *Gen. Comp. Endocrinol.* 192 237–245. 10.1016/j.ygcen.2013.05.020 23770021 PMC8715641

[B8] Dreborg S. Sundström G. Larsson T. A. Larhammar D. (2008). Evolution of vertebrate opioid receptors. *Proc. Natl. Acad. Sci. U.S.A.* 105 15487–15492. 10.1073/pnas.0805590105 18832151 PMC2563095

[B9] Dunn A. J. Berridge C. W. (1990). Physiological and behavioral responses to corticotropin-releasing factor administration: is CRF a mediator of anxiety or stress responses? *Brain Res. Rev.* 15 71–100. 10.1016/0165-0173(90)90012-D 1980834

[B10] Endsin M. J. Michalec O. Manzon L. A. Lovejoy D. A. Manzon R. G. (2017). CRH peptide evolution occurred in three phases: evidence from characterizing sea lamprey CRH system members. *Gen. Comp. Endocrinol.* 240 162–173. 10.1016/j.ygcen.2016.10.009 27777046

[B11] Fox J. H. Lowry C. A. (2013). Corticotropin-releasing factor-related peptides, serotonergic systems, and emotional behavior. *Front. Neurosci.* 7:169. 10.3389/fnins.2013.00169 24065880 PMC3778254

[B12] Fujimoto S. Oisi Y. Kuraku S. Ota K. G. Kuratani S. (2013). Non-parsimonious evolution of hagfish Dlx genes. *BMC Evol. Biol.* 13:15. 10.1186/1471-2148-13-15 23331926 PMC3552724

[B13] Gillis W. J. Bowerman B. Schneider S. Q. (2007). Ectoderm- and endomesoderm-specific GATA transcription factors in the marine annelid Platynereis dumerilli. *Evol. Dev.* 9 39–50. 10.1111/j.1525-142X.2006.00136.x 17227365

[B14] Gillis W. Q. Bowerman B. A. Schneider S. Q. (2008). The evolution of protostome GATA factors: molecular phylogenetics, synteny, and intron/exon structure reveal orthologous relationships. *BMC Evol. Biol.* 8:112. 10.1186/1471-2148-8-112 18412965 PMC2383905

[B15] Grantham R. Gautier C. Gouy M. Mercier R. Pavé A. (1980). Codon catalog usage and the genome hypothesis. *Nucleic Acids Res.* 8:197. 10.1093/nar/8.1.197-c 6986610 PMC327256

[B16] Grone B. P. Maruska K. P. (2015a). A second corticotropin-releasing hormone gene (CRH2) is conserved across vertebrate classes and expressed in the hindbrain of a basal Neopterygian fish, the spotted gar (Lepisosteus oculatus). *J. Comp. Neurol.* 523 1125–1143. 10.1002/cne.23729 25521515

[B17] Grone B. P. Maruska K. P. (2015b). Divergent evolution of two corticotropin-releasing hormone (CRH) genes in teleost fishes. *Front. Neurosci.* 9:365. 10.3389/fnins.2015.00365 26528116 PMC4602089

[B18] Gysling K. Forray M. I. Haeger P. Daza C. Rojas R. (2004). Corticotropin-releasing hormone and urocortin: redundant or distinctive functions? *Brain Res. Rev.* 47 116–125. 10.1016/j.brainresrev.2004.06.001 15572167

[B19] Hwang J. I. Moon M. J. Park S. Kim D. K. Cho E. B. Ha N. (2013). Expansion of secretin-like G protein-coupled receptors and their peptide ligands via local duplications before and after two rounds of whole-genome duplication. *Mol. Biol. Evol.* 30 1119–1130. 10.1093/molbev/mst031 23427277

[B20] Jaillon O. Aury J. M. Brunet F. Petit J. L. Stange-Thomann N. Maucell E. (2004). Genome duplication in the teleost fish Tetraodon nigroviridis reveals the early vertebrate proto-karyotype. *Nature* 431 946–957. 10.1038/nature03025 15496914

[B21] Kawada T. Sekiguchi T. Sakai T. Aoyama M. Satake H. (2010). Neuropeptides, hormone peptides, and their receptors in ciona intestinalis: an update. *Zoolog. Sci.* 27 134–153. 10.2108/zsj.27.134 20141419

[B22] Koob G. F. Heinrichs S. C. (1999). A role for corticotropin releasing factor and urocortin in behavioral responses to stressors. *Brain Res.* 848 141–152. 10.1016/S0006-8993(99)01991-5 10612706

[B23] Kuraku S. (2008). Insights into cyclostome phylogenomics: pre-2R or Post-2R. *Zool. Sci.* 25 960–968. 10.2108/zsj.25.960 19267631

[B24] Kuraku S. Kuratani S. (2006). Time scale for cyclostome evolution inferred with a phylogenetic diagnosis of hagfish and lamprey cDNA sequences. *Zool. Sci.* 23 1053–1064. 10.2108/zsj.23.1053 17261918

[B25] Lagman D. Ocampo Daza D. Widmark J. Abalo X. M. Sundström G. Larhammar D. (2013). The vertebrate ancestral repertoire of visual opsins, transducin alpha subunits and oxytocin/vasopressin receptors was established by duplication of their shared genomic region in the two rounds of early vertebrate genome duplications. *BMC Evol. Biol.* 13:238. 10.1186/1471-2148-13-238 24180662 PMC3826523

[B26] Larsson A. (2014). AliView: a fast and lightweight alignment viewer and editor for large datasets. *Bioinformatics* 30 3276–3278. 10.1093/bioinformatics/btu531 25095880 PMC4221126

[B27] Le S. Q. Gascuel O. (2008). An improved general amino acid replacement matrix. *Mol. Biol. Evol.* 25 1307–1320. 10.1093/molbev/msn067 18367465

[B28] Lefort V. Longueville J. E. Gascuel O. (2017). SMS: smart Model Selection in PhyML. *Mol. Biol. Evol.* 34 2422–2424. 10.1093/molbev/msx149 28472384 PMC5850602

[B29] Lewis K. Li C. Perrin M. H. Blount A. Kunitake K. Donaldson C. (2001). Identification of urocortin III, an additional member of the corticotropin-releasing factor (CRF) family with high affinity for the CRF2 receptor. *Proc. Natl. Acad. Sci. U.S.A.* 98 7570–7575. 10.1073/pnas.121165198 11416224 PMC34709

[B30] Lovejoy D. A. Balment R. J. (1999). Evolution and physiology of the corticotropin-releasing factor (CRF) family of neuropeptides in vertebrates. *Gen. Comp. Endocrinol.* 115 1–22. 10.1006/gcen.1999.7298 10375459

[B31] Lovejoy D. A. de Lannoy L. (2013). Evolution and phylogeny of the corticotropin-releasing factor (CRF) family of peptides: expansion and specialization in the vertebrates. *J. Chem. Neuroanat.* 54 50–56. 10.1016/j.jchemneu.2013.09.006 24076419

[B32] Manousaki T. Qiu H. Noro M. Hildebrand F. Meyer A. Kuraku S. (2016). “Molecular evolution in the lamprey genomes and its relevance to the timing of whole genome duplications,” in *Jawless Fishes of the World?*, Vol. 1 eds Orlov A. Beamish R. (Newcastle: Cambridge Scholars Publishing), 2–16.

[B33] Mehta T. K. Raèi È Yamasaki S. Lee A. P. Lian M. M. Tay B. H. (2013). Evidence for at least six Hox clusters in the Japanese lamprey (*Lethenteron japonicum*). *Proc. Natl. Acad. Sci. U.S.A.* 110 16044–16049. 10.1073/pnas.1315760110 24043829 PMC3791769

[B34] Miller M. A. Pfeiffer W. Schwartz T. (2010). “Creating the CIPRES Science Gateway for inference of large phylogenetic trees,” in *2010 Gateway Computing Environments Workshop, GCE 2010*, New Orleans, LA.

[B35] Mirabeau O. Joly J. S. (2013). Molecular evolution of peptidergic signaling systems in bilaterians. *Proc. Natl. Acad. Sci. U.S.A.* 110 E2028–E2203. 10.1073/pnas.1219956110 23671109 PMC3670399

[B36] Nah G. S. S. Tay B. H. Brenner S. Osato M. Venkatesh B. (2014). Characterization of the runx gene family in a jawless vertebrate, the Japanese lamprey (*Lethenteron japonicum*). *PLoS One* 9:e113445. 10.1371/journal.pone.0113445 25405766 PMC4236176

[B37] Nakatani Y. Takeda H. Kohara Y. Morishita S. (2007). Reconstruction of the vertebrate ancestral genome reveals dynamic genome reorganization in early vertebrates. *Genome Res.* 17 1254–1265. 10.1101/gr.6316407 17652425 PMC1950894

[B38] Nock T. G. Chand D. Lovejoy D. A. (2011). Identification of members of the gonadotropin-releasing hormone (GnRH), corticotropin-releasing factor (CRF) families in the genome of the holocephalan, *Callorhinchus milii* (elephant shark). *Gen. Comp. Endocrinol.* 171 237–244. 10.1016/j.ygcen.2011.02.001 21310155

[B39] Palidwor G. A. Perkins T. J. Xia X. (2010). A general model of Codon bias due to GC mutational bias. *PLoS One* 5:e13431. 10.1371/journal.pone.0013431 21048949 PMC2965080

[B40] Putnam N. H. Butts T. Ferrier D. E. K. Furlong R. F. Hellsten U. Kawashima T. (2008). The amphioxus genome and the evolution of the chordate karyotype. *Nature* 453 1064–1071. 10.1038/nature06967 18563158

[B41] Qin H. Wu W. B. Comeron J. M. Kreitman M. Li W. H. (2004). Intragenic spatial patterns of codon usage bias in prokaryotic and eukaryotic genomes. *Genetics* 68 2245–2260. 10.1534/genetics.104.030866 15611189 PMC1448744

[B42] Qiu H. Hildebrand F. Kuraku S. Meyer A. (2011). Unresolved orthology and peculiar coding sequence properties of lamprey genes: the KCNA gene family as test case. *BMC Genomics* 12:325. 10.1186/1471-2164-12-325 21699680 PMC3141671

[B43] Reyes T. M. Lewis K. Perrin M. H. Kunitake K. S. Vaughan J. Arias C. A. (2001). Urocortin II: a member of the corticotropin-releasing factor (CRF) neuropeptide family that is selectively bound by type 2 CRF receptors. *Proc. Natl. Acad. Sci. U.S.A.* 98 2843–2848. 10.1073/pnas.051626398 11226328 PMC30227

[B44] Roberts B. W. Didier W. Rai S. Johnson N. S. Libants S. Yun S. S. (2014). Regulation of a putative corticosteroid, 17,21-dihydroxypregn-4-ene,3,20-one, in sea lamprey, *Petromyzon marinus*. *Gen. Comp. Endocrinol.* 196 17–25. 10.1016/j.ygcen.2013.11.008 24287339

[B45] Romiguier J. Ranwez V. Douzery E. J. P. Galtier N. (2010). Contrasting GC-content dynamics across 33 mammalian genomes: relationship with life-history traits and chromosome sizes. *Genome Res.* 20 1001–1009. 10.1101/gr.104372.109 20530252 PMC2909565

[B46] Ronquist F. Teslenko M. Van Der Mark P. Ayres D. L. Darling A. Höhna S. (2012). MrBayes 3.2: efficient bayesian phylogenetic inference and model choice across a large model space. *Syst. Biol.* 61 539–542. 10.1093/sysbio/sys029 22357727 PMC3329765

[B47] Sacerdot C. Louis A. Bon C. Berthelot C. Roest Crollius H. (2018). Chromosome evolution at the origin of the ancestral vertebrate genome. *Genome Biol.* 19:166. 10.1186/s13059-018-1559-1 30333059 PMC6193309

[B48] Shibahara S. Morimoto Y. Furutani Y. Notake M. Takahashi H. Shimizu S. (1983). Isolation and sequence analysis of the human corticotropin-releasing factor precursor gene. *EMBO J.* 2 775–779. 10.1002/j.1460-2075.1983.tb01499.x 6605851 PMC555184

[B49] Sievers F. Wilm A. Dineen D. Gibson T. J. Karplus K. Li W. (2011). Fast, scalable generation of high-quality protein multiple sequence alignments using clustal omega. *Mol. Syst. Biol.* 7:539. 10.1038/msb.2011.75 21988835 PMC3261699

[B50] Simakov O. Marlétaz F. Yue J.-X. O’Connell B. Jenkins J. Brandt A. (2020). Deeply conserved synteny resolves early events in vertebrate evolution. *Nat. Ecol. Evol.* 4 820–830. 10.1038/s41559-020-1156-z 32313176 PMC7269912

[B51] Smith J. J. Keinath M. C. (2015). The sea lamprey meiotic map improves resolution of ancient vertebrate genome duplications. *Genome Res.* 25 1081–1090. 10.1101/gr.184135.114 26048246 PMC4509993

[B52] Smith J. J. Kuraku S. Holt C. Sauka-Spengler T. Jiang N. Campbell M. S. (2013). Sequencing of the sea lamprey (*Petromyzon marinus*) genome provides insights into vertebrate evolution. *Nat. Genet.* 45 415–421. 10.1038/ng.2568 23435085 PMC3709584

[B53] Smith J. J. Timoshevskaya N. Ye C. Holt C. Keinath M. C. Parker H. J. (2018). The sea lamprey germline genome provides insights into programmed genome rearrangement and vertebrate evolution. *Nat. Genet.* 50 270–277. 10.1038/s41588-017-0036-1 29358652 PMC5805609

[B54] Stanke M. Diekhans M. Baertsch R. Haussler D. (2008). Using native and syntenically mapped cDNA alignments to improve de novo gene finding. *Bioinformatics* 24 637–644. 10.1093/bioinformatics/btn013 18218656

[B55] Stanke M. Steinkamp R. Waack S. Morgenstern B. (2004). AUGUSTUS: a web server for gene finding in eukaryotes. *Nucleic Acids Res.* 32 W309–W312. 10.1093/nar/gkh379 15215400 PMC441517

[B56] Sundström G. Dreborg S. Larhammar D. (2010). Concomitant duplications of opioid peptide and receptor genes before the origin of jawed vertebrates. *PLoS One* 5:e10512. 10.1371/journal.pone.0010512 20463905 PMC2865548

[B57] Thompson R. C. Seasholtz A. F. Herbert E. (1987). Rat corticotropin-releasing hormone gene: sequence and tissue-specific expression. *Mol. Endocrinol.* 1 363–370. 10.1210/mend-1-5-3 3274895

[B58] Tostivint H. Dettaï A. Quan F. B. Ravi V. Tay B. H. Rodicio M. C. (2016). Identification of three somatostatin genes in lampreys. *Gen. Comp. Endocrinol.* 237 89–97. 10.1016/j.ygcen.2016.08.006 27524287

[B59] Vale W. Spiess J. Rivier C. Rivier J. (1981). Characterization of a 41-residue ovine hypothalamic peptide that stimulates secretion of corticotropin and β-endorphin. *Science* 213 1394–1397. 10.1126/science.6267699 6267699

[B60] Wotton K. R. Shimeld S. M. (2011). Analysis of lamprey clustered Fox genes: insight into Fox gene evolution and expression in vertebrates. *Gene* 489 30–40. 10.1016/j.gene.2011.08.007 21907770

[B61] Zhang H. Ravi V. Tay B. H. Tohari S. Pillai N. E. Prasad A. (2017). Lampreys, the jawless vertebrates, contain only two ParaHox gene clusters. *Proc. Natl. Acad. Sci. U.S.A.* 114 9146–9151. 10.1073/pnas.1704457114 28784804 PMC5576799

