# Supplementary material for: Corticotropin-Releasing Hormone (CRH) Gene Family Duplications in Lampreys Correlate With Two Early Vertebrate Genome Doublings
Source: Front Neurosci. 2020 Jul 30;14:672. doi: 10.3389/fnins.2020.00672 (PMC7406891; doi:10.3389/fnins.2020.00672)
Supplement: FIGURE S1 — Bayesian inference tree of the CRH mature peptides. Branch support values (posterior probability values) are shown and tree was rooted with the tunicate CRH-like precursors. Tree was rooted with the tunicate CRH-family members. A simplified radial tree is represented in Figure 1. Accession numbers of the sequences used are available in Supplementary Table S2. [file Data_Sheet_1.zip › Figure S5.PDF]

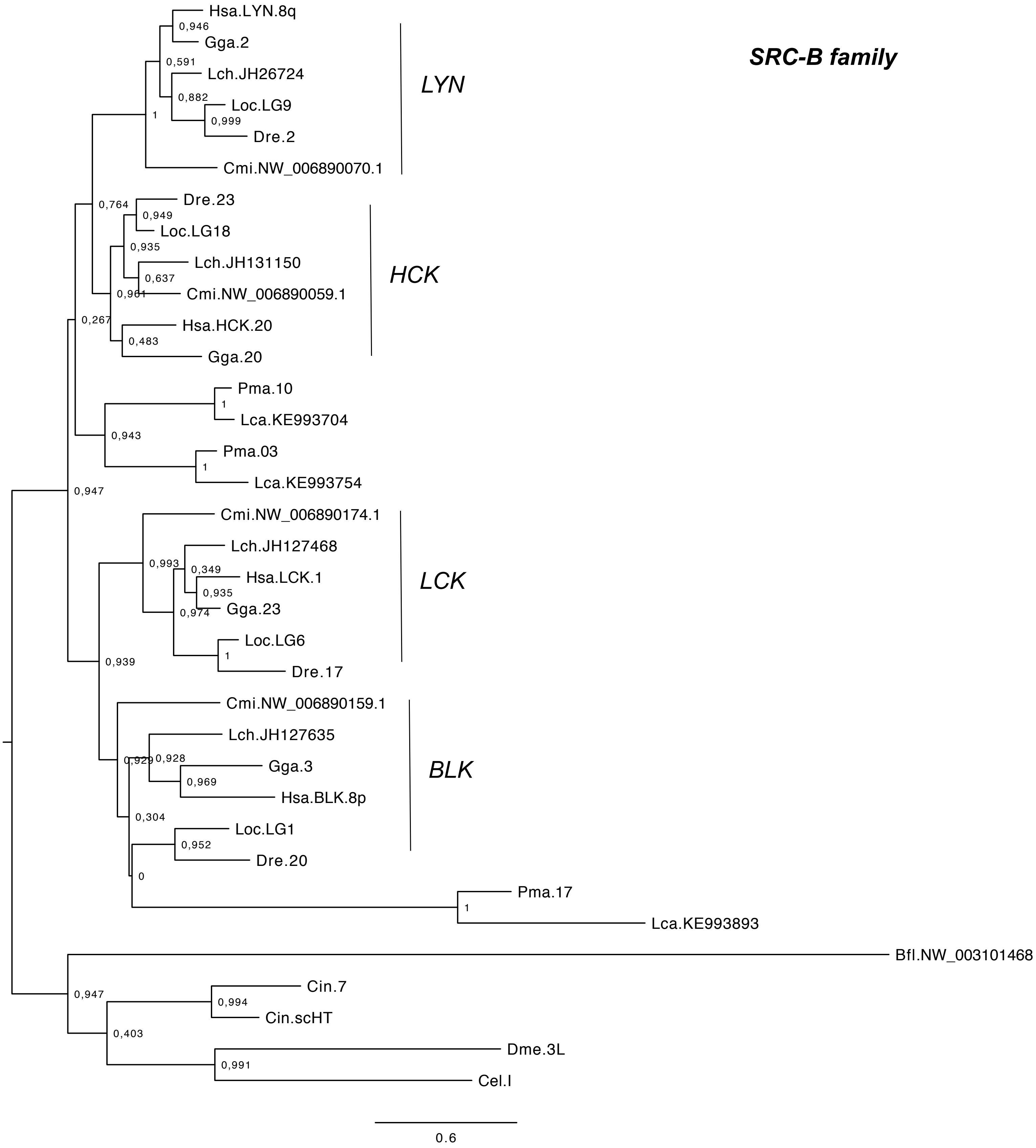
SRC-B family
LYN
HCK
LCK
BLK
Hsa.LYN.8q
Gga.2
Lch.JH26724
Loc.LG9
Dre.2
Cmi.NW_006890070.1
Dre.23
Loc.LG18
Lch.JH131150
Cmi.NW_006890059.1
Hsa.HCK.20
Gga.20
Pma.10
Lca.KE993704
Pma.03
Lca.KE993754
Cmi.NW_006890174.1
Lch.JH127468
Hsa.LCK.1
Gga.23
Loc.LG6
Dre.17
Cmi.NW_006890159.1
Lch.JH127635
Gga.3
Hsa.BLK.8p
Loc.LG1
Dre.20
Pma.17
Lca.KE993893
Bfl.NW_003101468
Cin.7
Cin.scHT
Dme.3L
Cel.I
0,946
0,591
0,882
0,999
1
0,764
0,949
0,935
0,637
0,961
0,267
0,483
1
0,943
1
0,947
0,993
0,349
0,935
0,974
1
0,939
0,929
0,928
0,969
0,304
0,952
0
1
0,947
0,994
0,403
0,991
0.6

SLC30A family

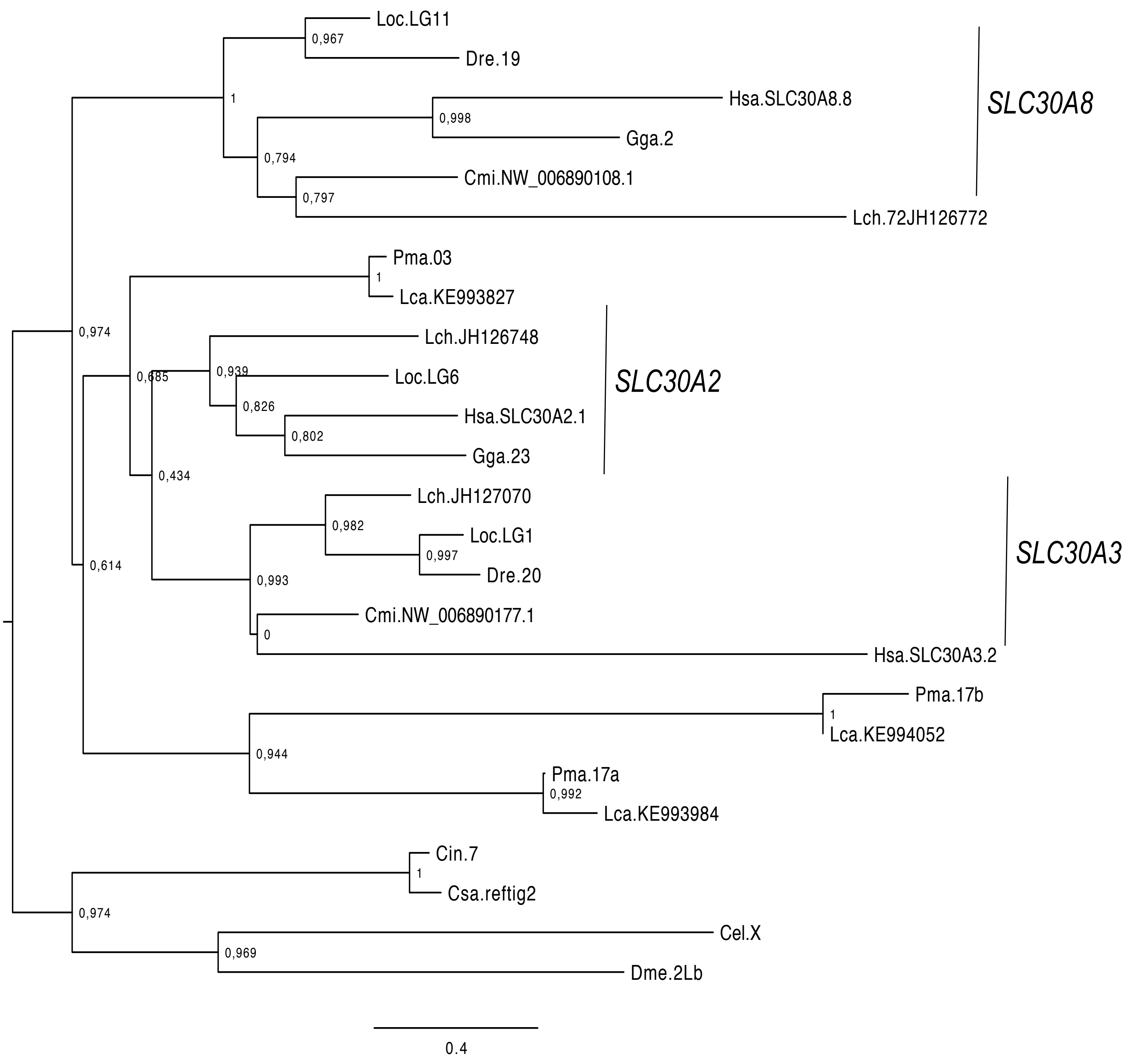

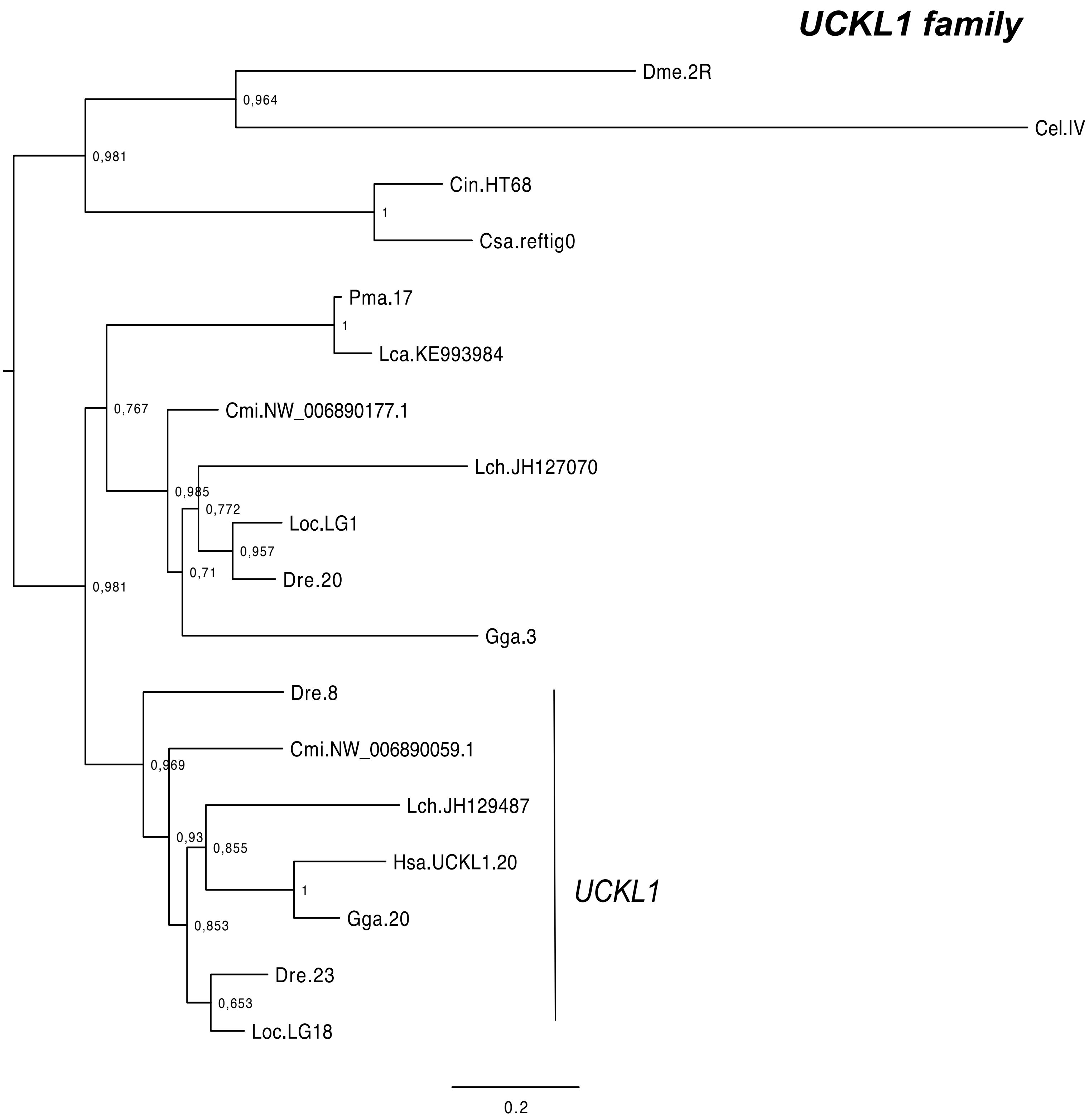
UCKL1 family
Dme.2R
0,964
Cel.IV
0,981
Cin.HT68
1
Csa.reftig0
Pma.17
1
Lca.KE993984
0,767
Cmi.NW_006890177.1
Lch.JH127070
0,985
0,772
Loc.LG1
0,957
Dre.20
0,71
0,981
Gga.3
Dre.8
Cmi.NW_006890059.1
0,969
Lch.JH129487
0,93
0,855
Hsa.UCKL1.20
1
UCKL1
Gga.20
0,853
Dre.23
0,653
Loc.LG18
0.2

MVP17 family

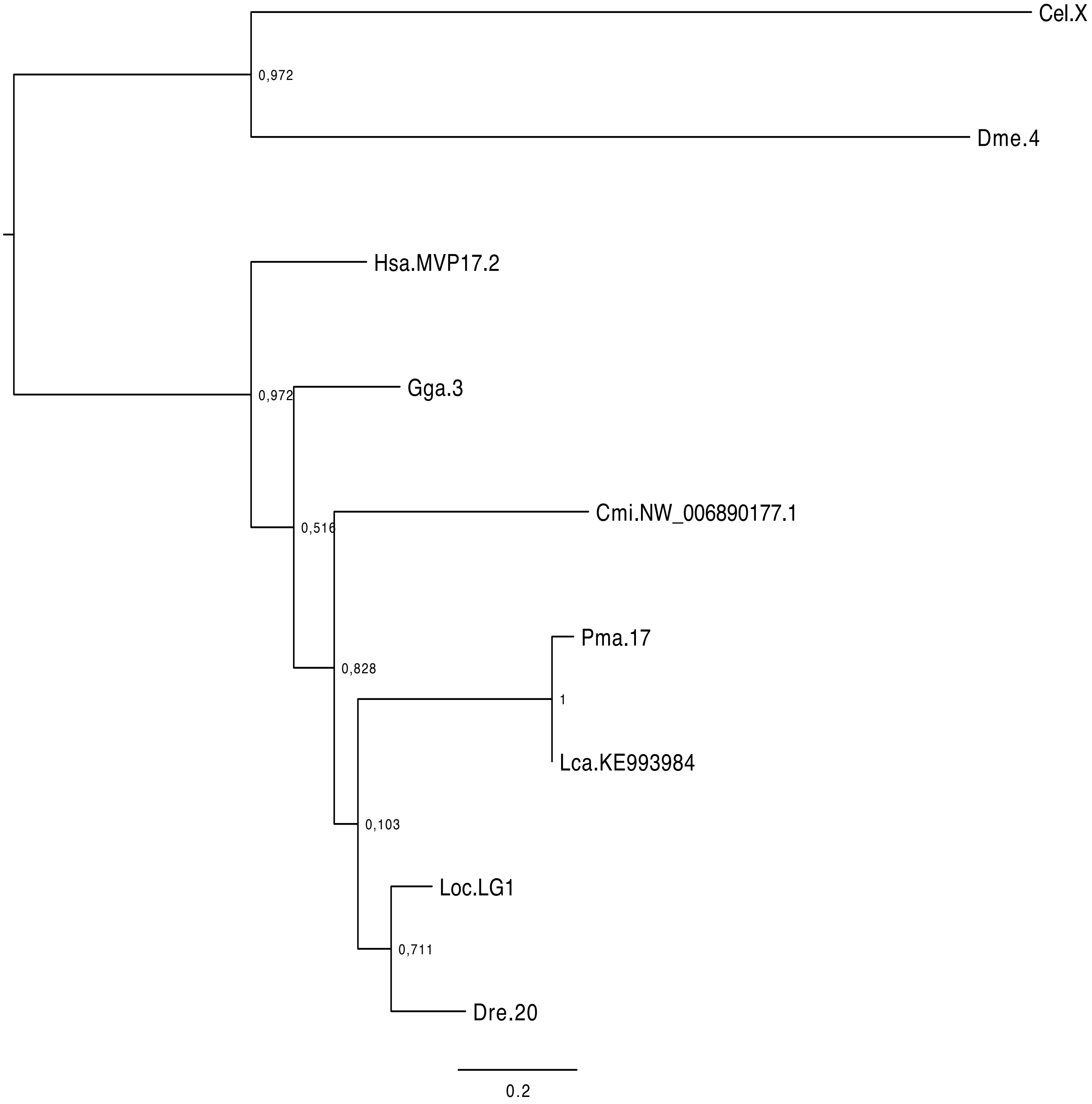

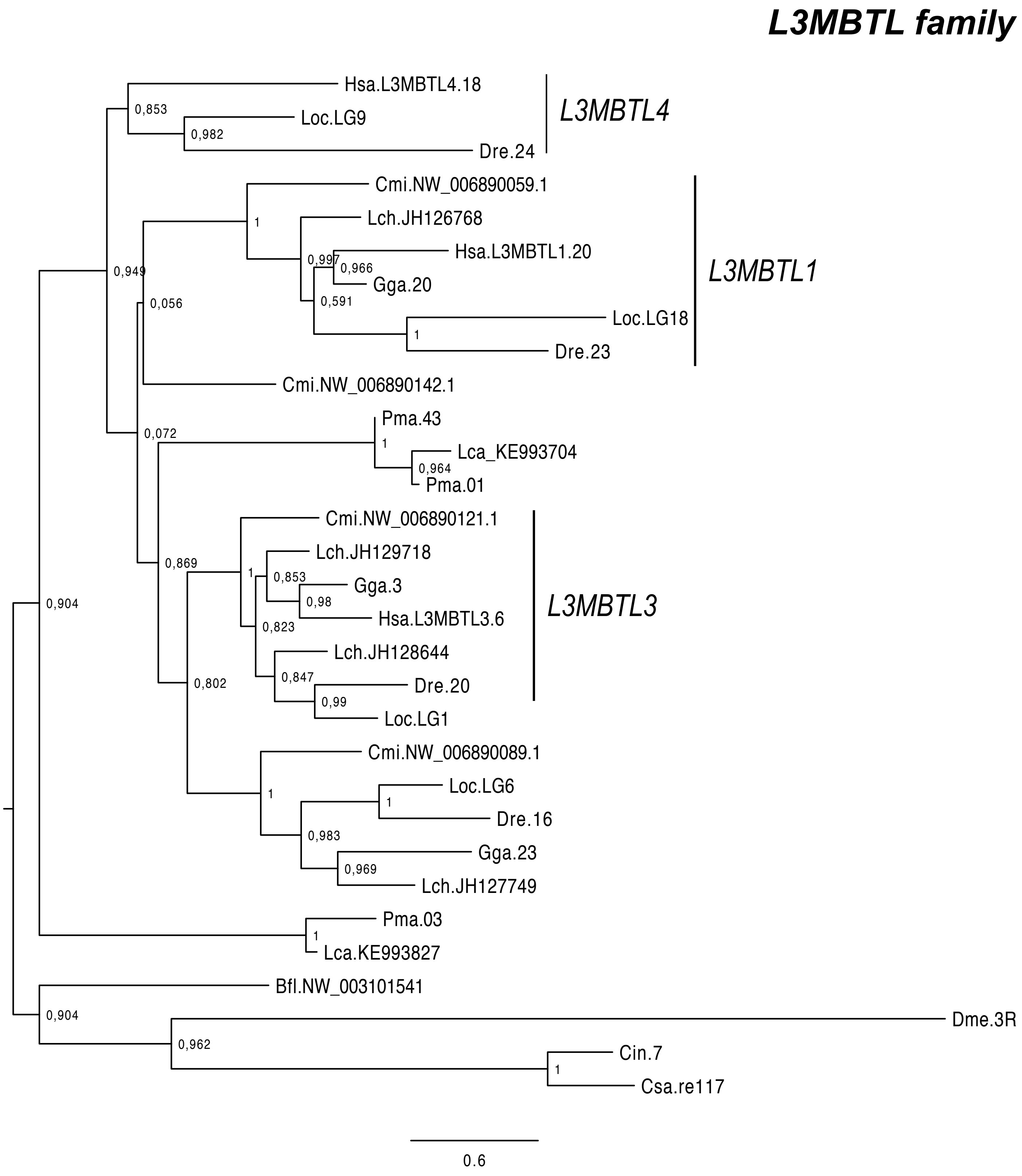
L3MBTL family
Hsa.L3MBTL4.18
Loc.LG9
Dre.24
L3MBTL4
Cmi.NW_006890059.1
Lch.JH126768
Hsa.L3MBTL1.20
Gga.20
Loc.LG18
Dre.23
L3MBTL1
Cmi.NW_006890142.1
Pma.43
Lca_KE993704
Pma.01
Cmi.NW_006890121.1
Lch.JH129718
Gga.3
Hsa.L3MBTL3.6
Lch.JH128644
Dre.20
Loc.LG1
L3MBTL3
Cmi.NW_006890089.1
Loc.LG6
Dre.16
Gga.23
Lch.JH127749
Pma.03
Lca.KE993827
Bfl.NW_003101541
Dme.3R
Cin.7
Csa.re117
0,853
0,982
1
0,997
0,966
0,591
1
0,949
0,056
0,072
1
0,964
0,869
1
0,853
0,98
0,823
0,847
0,99
0,802
1
1
0,983
0,969
0,904
1
0,904
0,962
1
0.6

MECR family

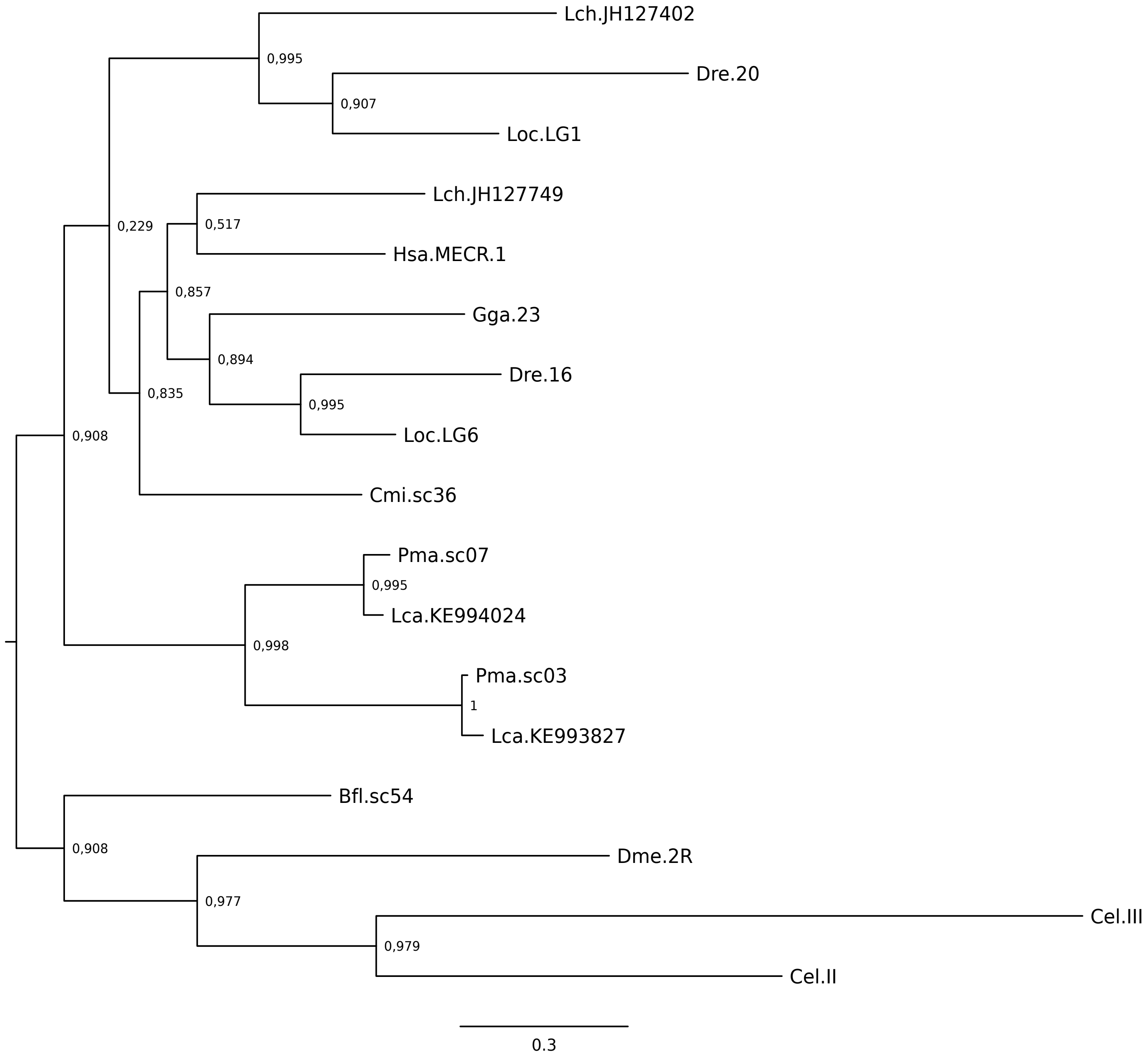

Lch.JH127402
0,995
Dre.20
0,907
Loc.LG1
Lch.JH127749
0,229
0,517
Hsa.MECR.1
0,857
Gga.23
0,894
Dre.16
0,835
0,995
0,908
Loc.LG6
Cmi.sc36
Pma.sc07
0,995
Lca.KE994024
0,998
Pma.sc03
1
Lca.KE993827
Bfl.sc54
0,908
Dme.2R
0,977
Cel.III
0,979
Cel.II
0.3

RPA2 family

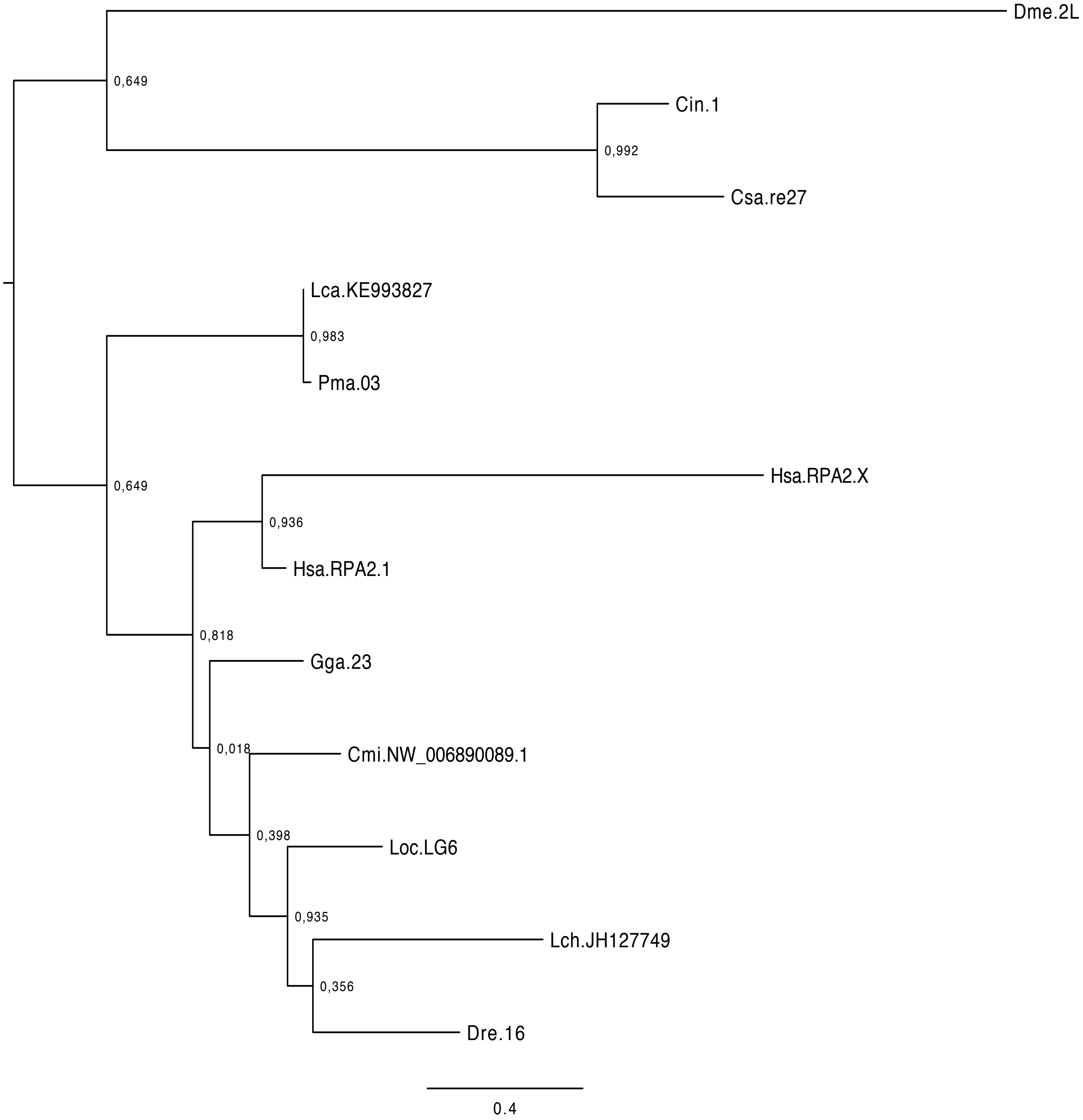
Dme.2L
0,649
Cin.1
0,992
Csa.re27
Lca.KE993827
0,983
Pma.03
0,649
Hsa.RPA2.X
0,936
Hsa.RPA2.1
0,818
Gga.23
0,018
Cmi.NW_006890089.1
0,398
Loc.LG6
0,935
Lch.JH127749
0,356
Dre.16
0.4

VNN/BTD family

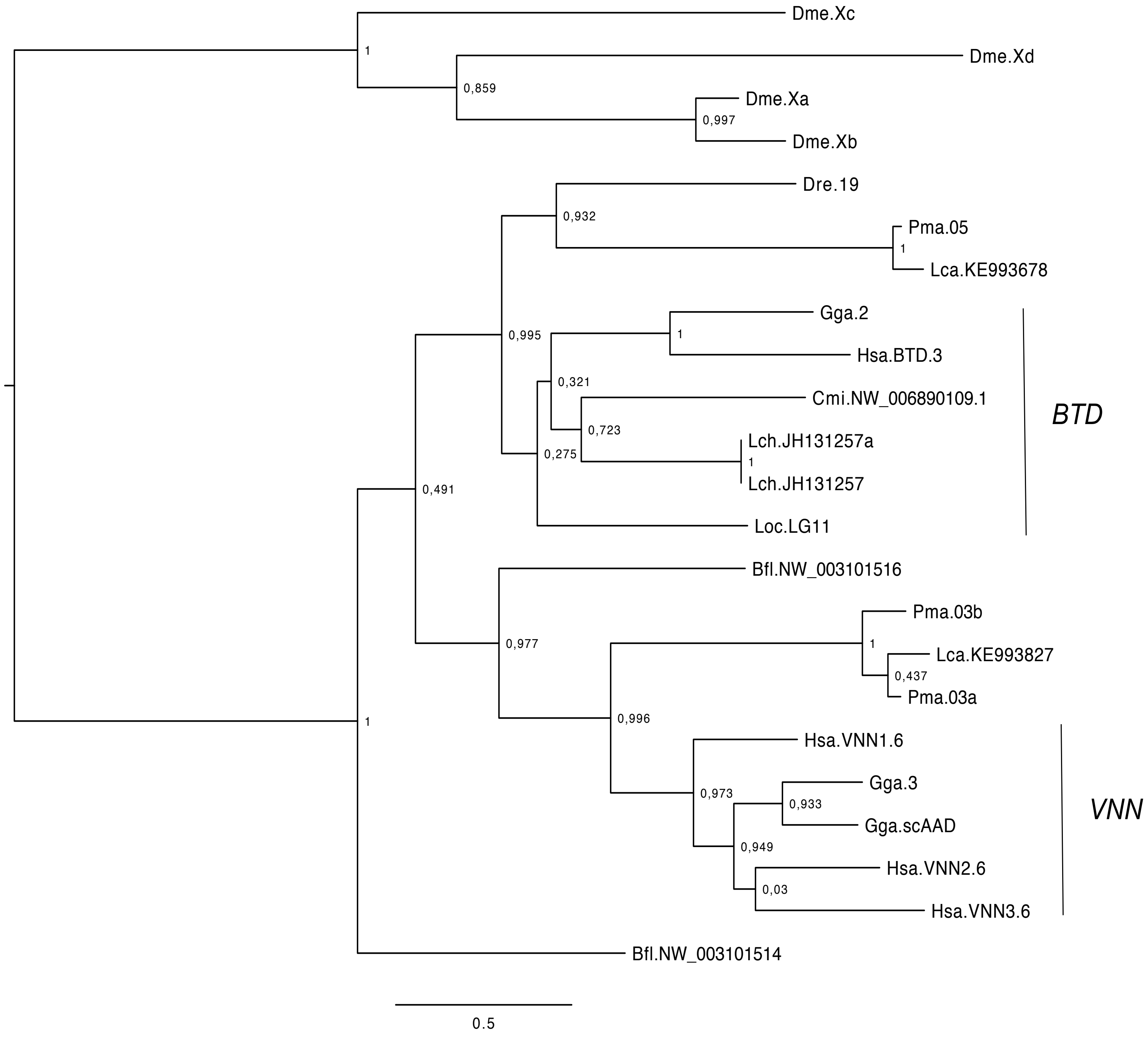

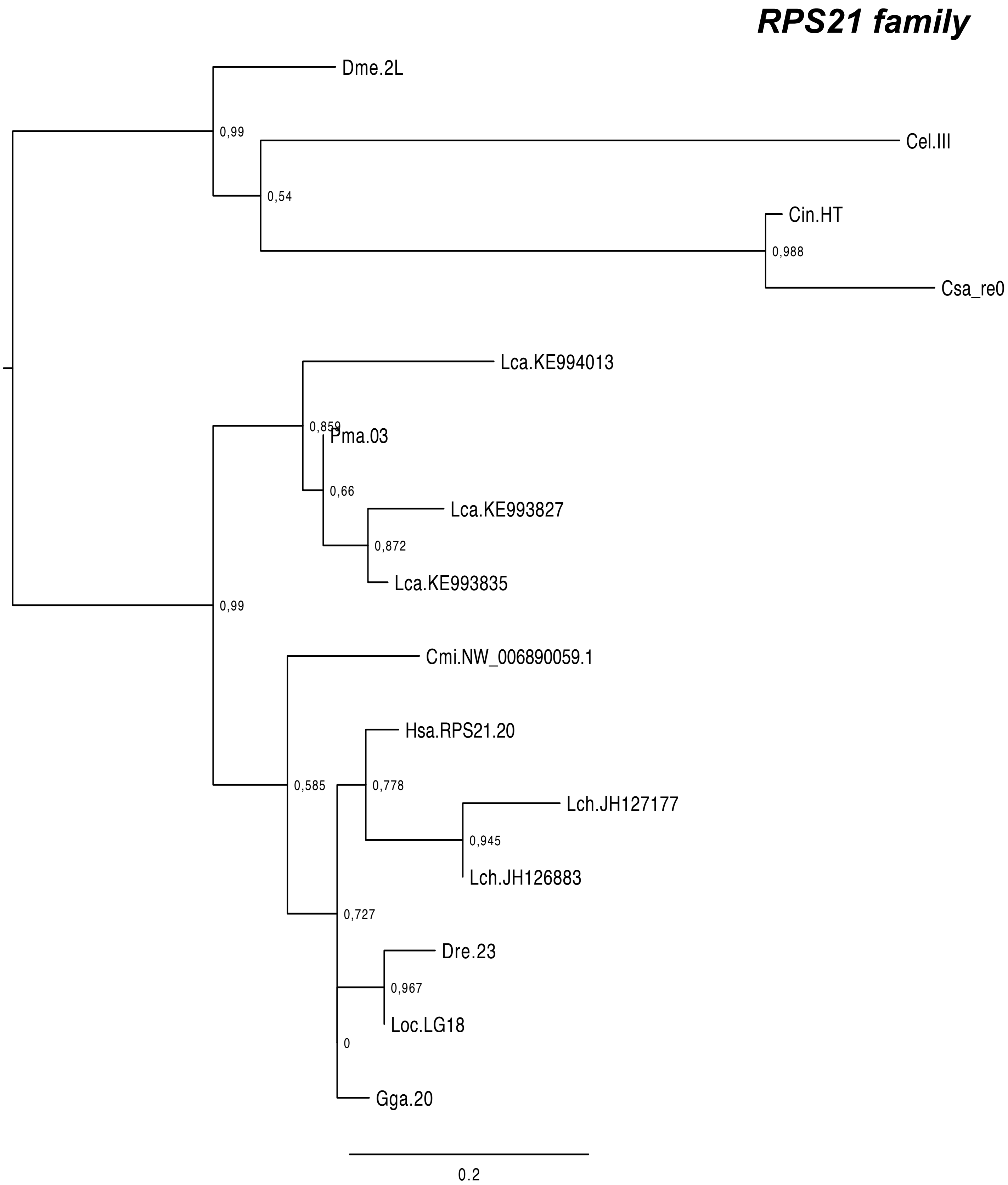

RPS21 family
Dme.2L
0,99
Cel.III
0,54
Cin.HT
0,988
Csa_re0
Lca.KE994013
0,859
Pma.03
0,66
Lca.KE993827
0,872
Lca.KE993835
0,99
Cmi.NW_006890059.1
Hsa.RPS21.20
0,585
0,778
Lch.JH127177
0,945
Lch.JH126883
0,727
Dre.23
0,967
Loc.LG18
0
Gga.20
0.2

TRNAUP1 family

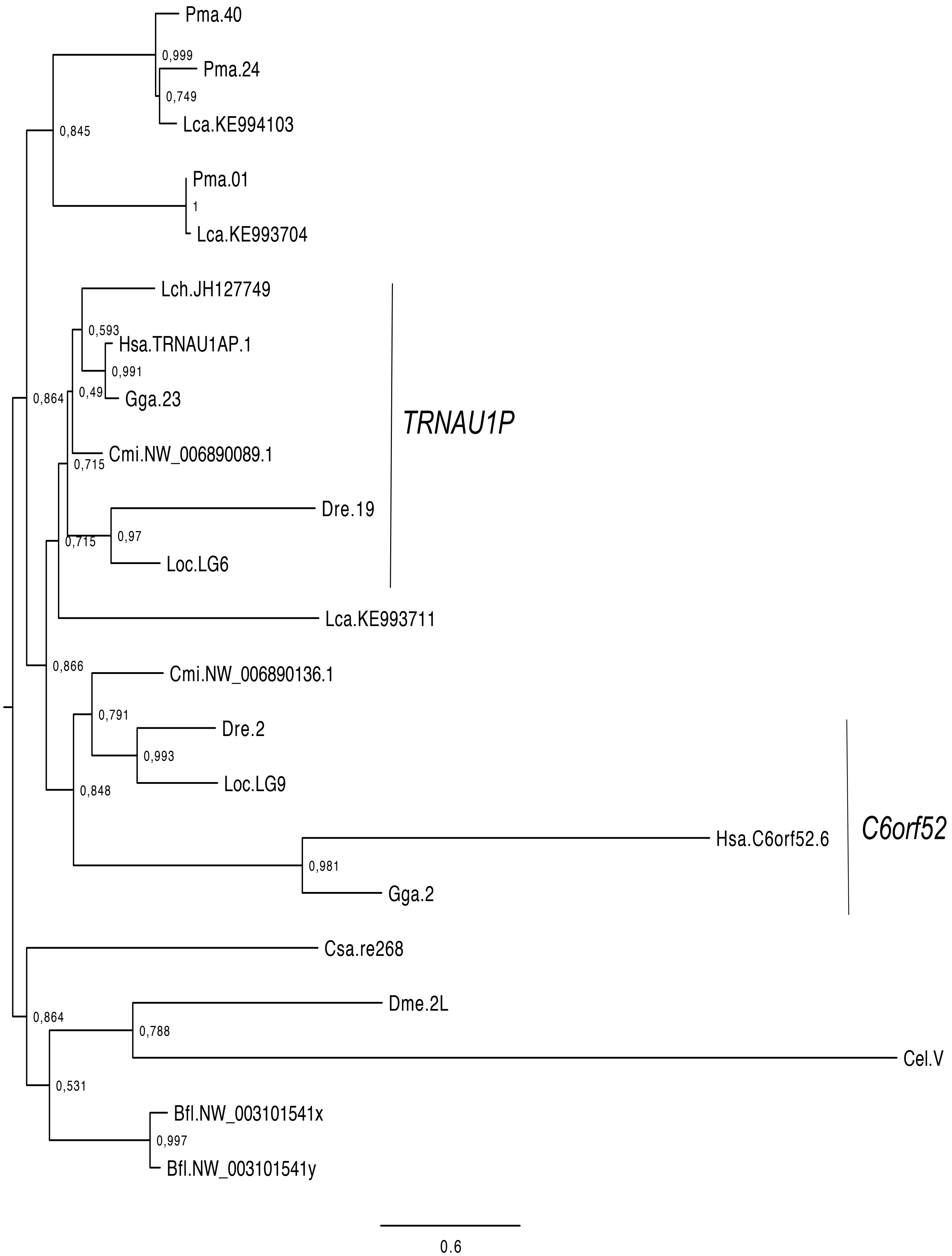

PAK1IP family

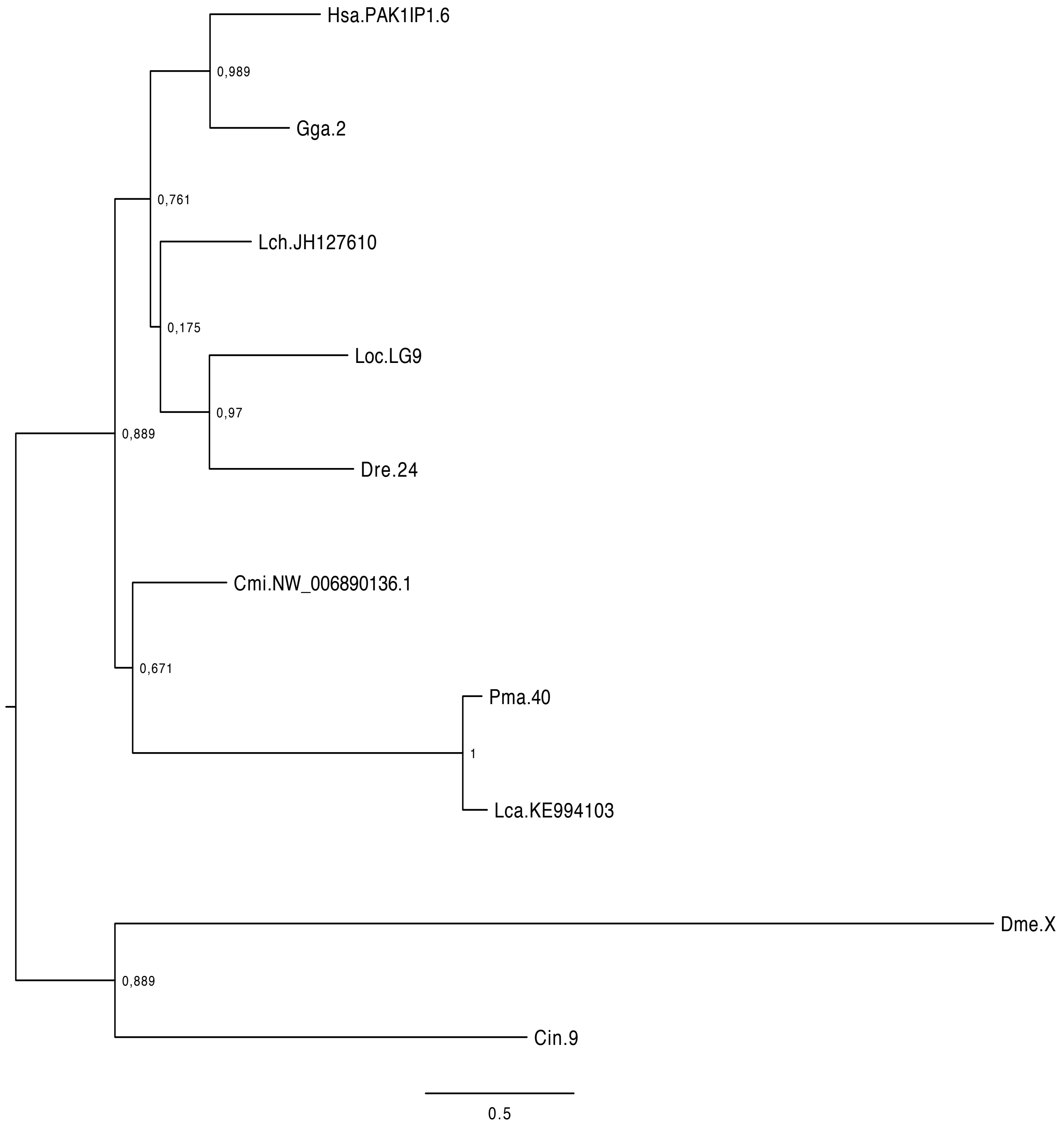

GAREM family

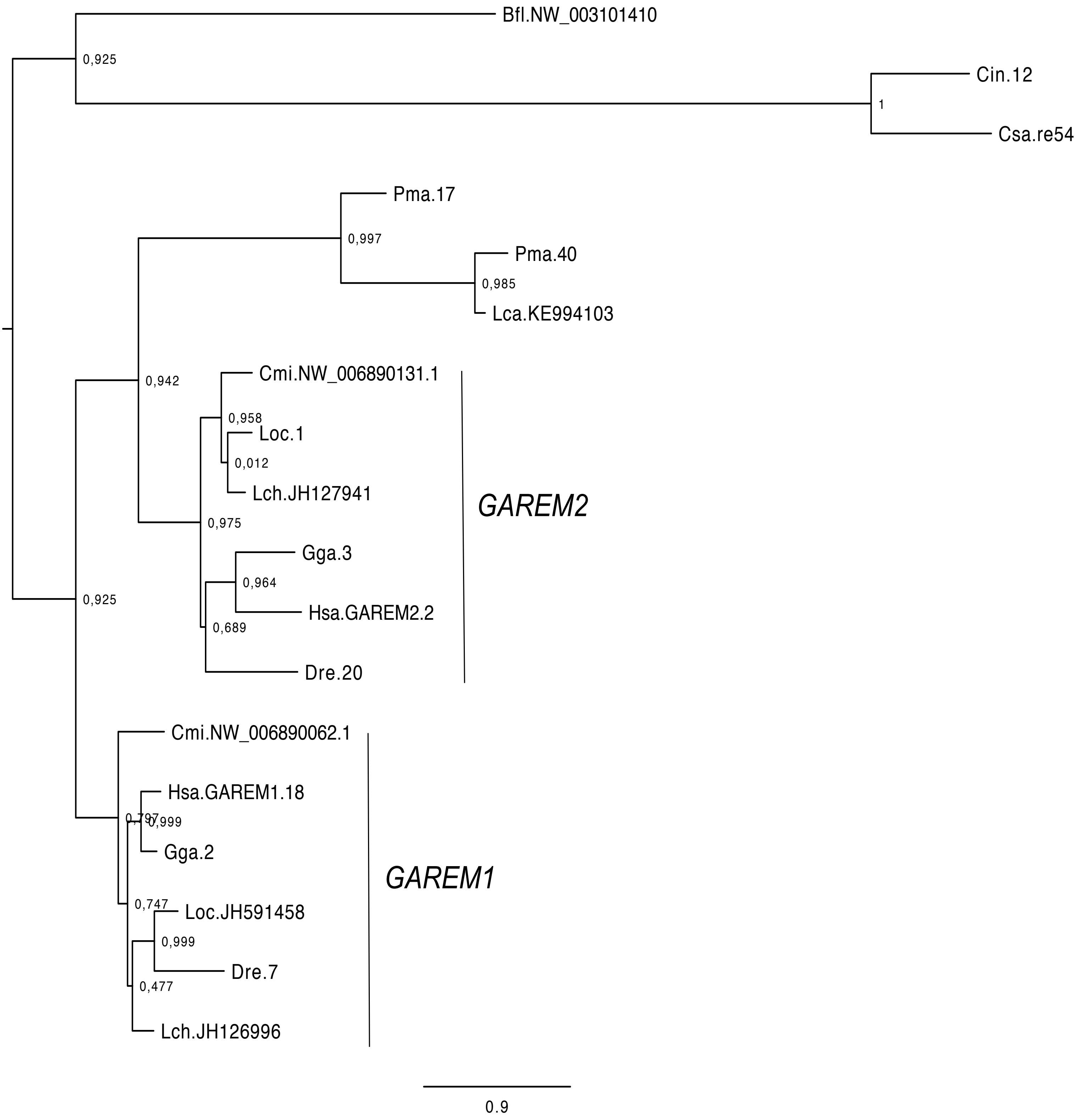

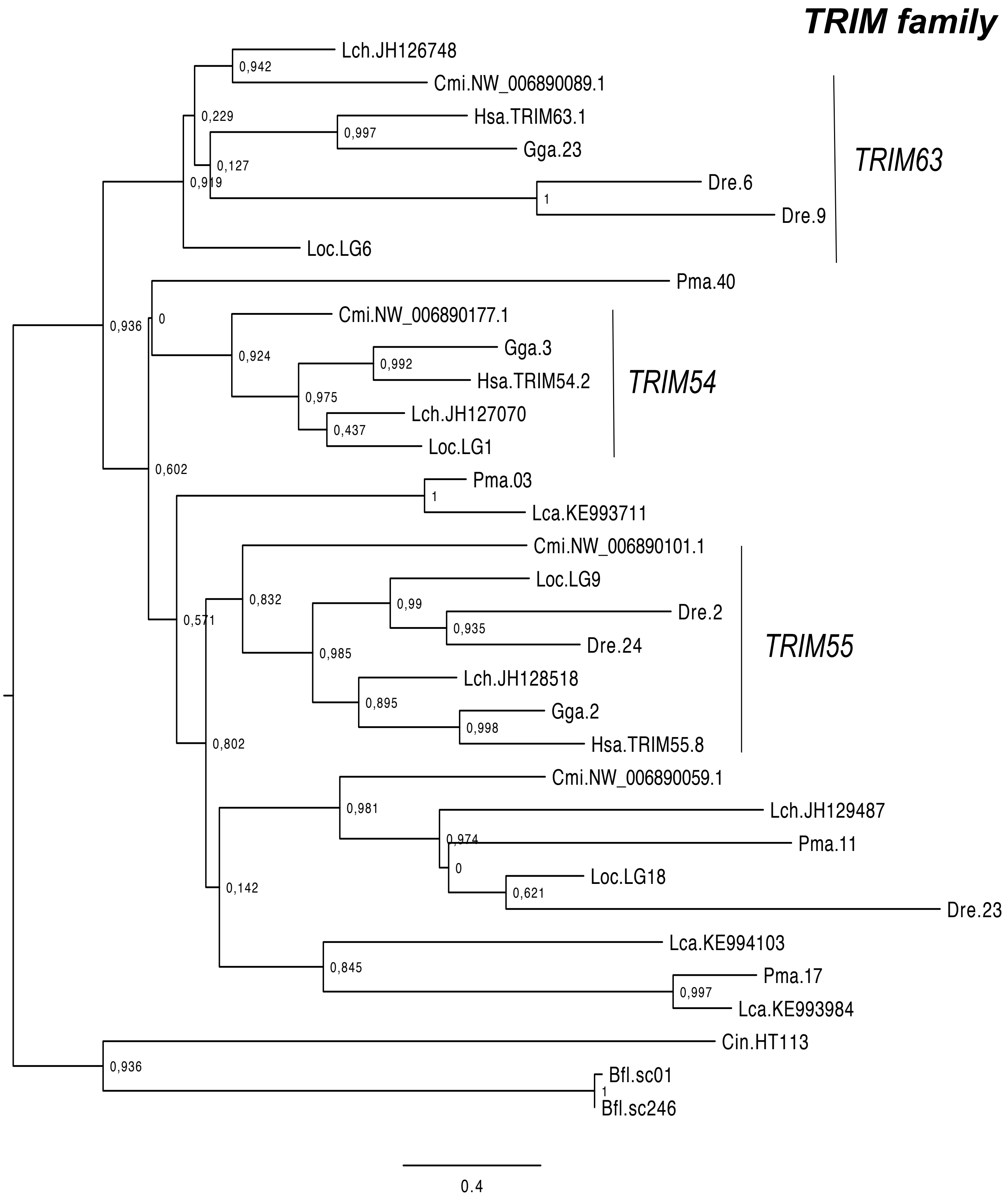
TRIM family
Lch.JH126748
Cmi.NW_006890089.1
Hsa.TRIM63.1
Gga.23
Dre.6
Dre.9
Loc.LG6
TRIM63
Pma.40
Cmi.NW_006890177.1
Gga.3
Hsa.TRIM54.2
Lch.JH127070
Loc.LG1
TRIM54
Pma.03
Lca.KE993711
Cmi.NW_006890101.1
Loc.LG9
Dre.2
Dre.24
Lch.JH128518
Gga.2
Hsa.TRIM55.8
TRIM55
Cmi.NW_006890059.1
Lch.JH129487
Pma.11
Loc.LG18
Dre.23
Lca.KE994103
Pma.17
Lca.KE993984
Cin.HT113
Bfl.sc01
Bfl.sc246
0,942
0,229
0,997
0,127
0,919
1
0,936
0
0,924
0,992
0,975
0,437
0,602
1
0,571
0,832
0,99
0,935
0,985
0,895
0,998
0,802
0,981
0,974
0
0,621
0,142
0,845
0,997
0,936
1
0.4

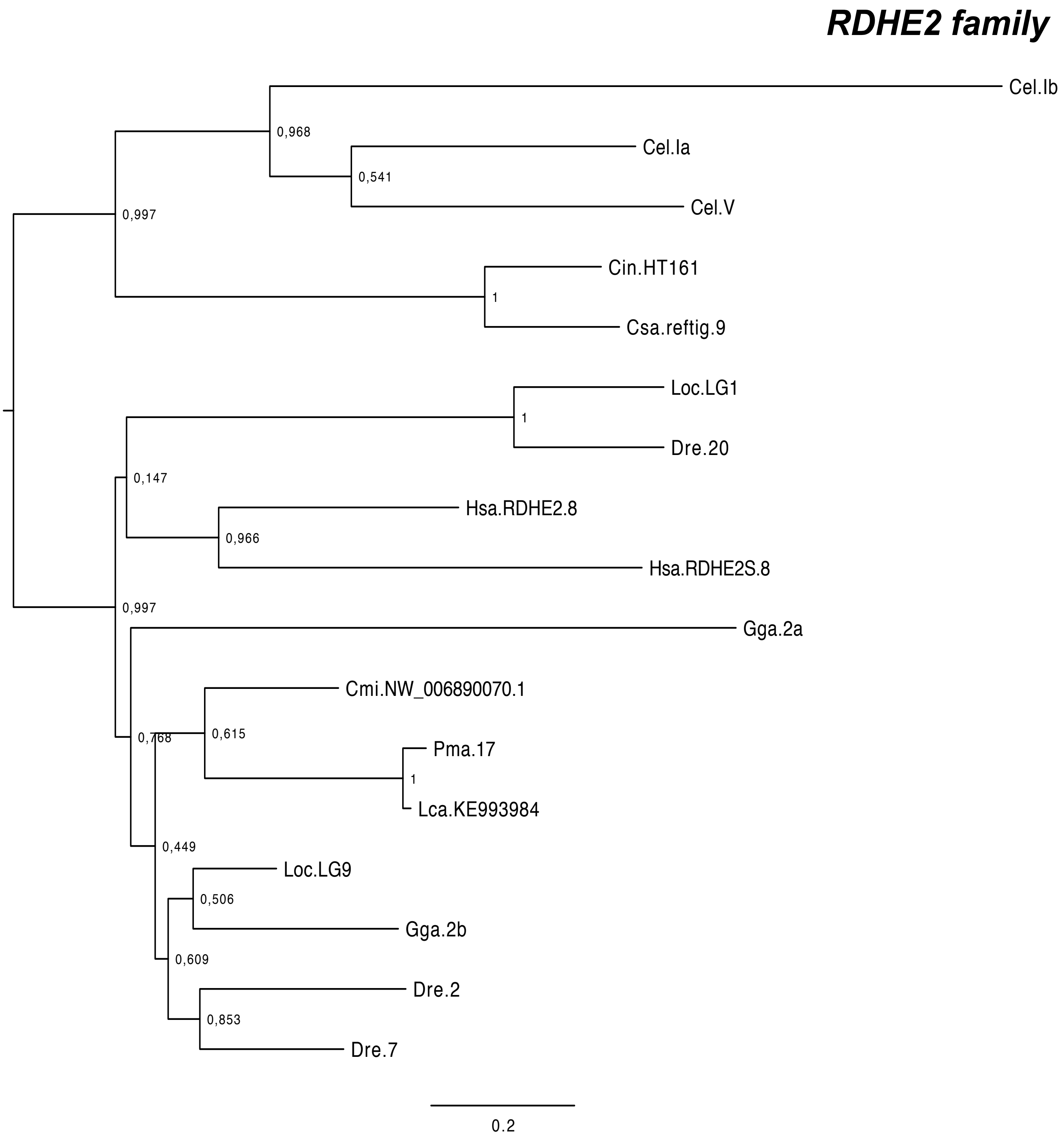
RDHE2 family
Cel.Ib
0,968
Cel.Ia
0,541
Cel.V
0,997
Cin.HT161
1
Csa.reftig.9
Loc.LG1
1
Dre.20
0,147
Hsa.RDHE2.8
0,966
Hsa.RDHE2S.8
0,997
Gga.2a
Cmi.NW_006890070.1
0,768
0,615
Pma.17
1
Lca.KE993984
0,449
Loc.LG9
0,506
Gga.2b
0,609
Dre.2
0,853
Dre.7
0.2

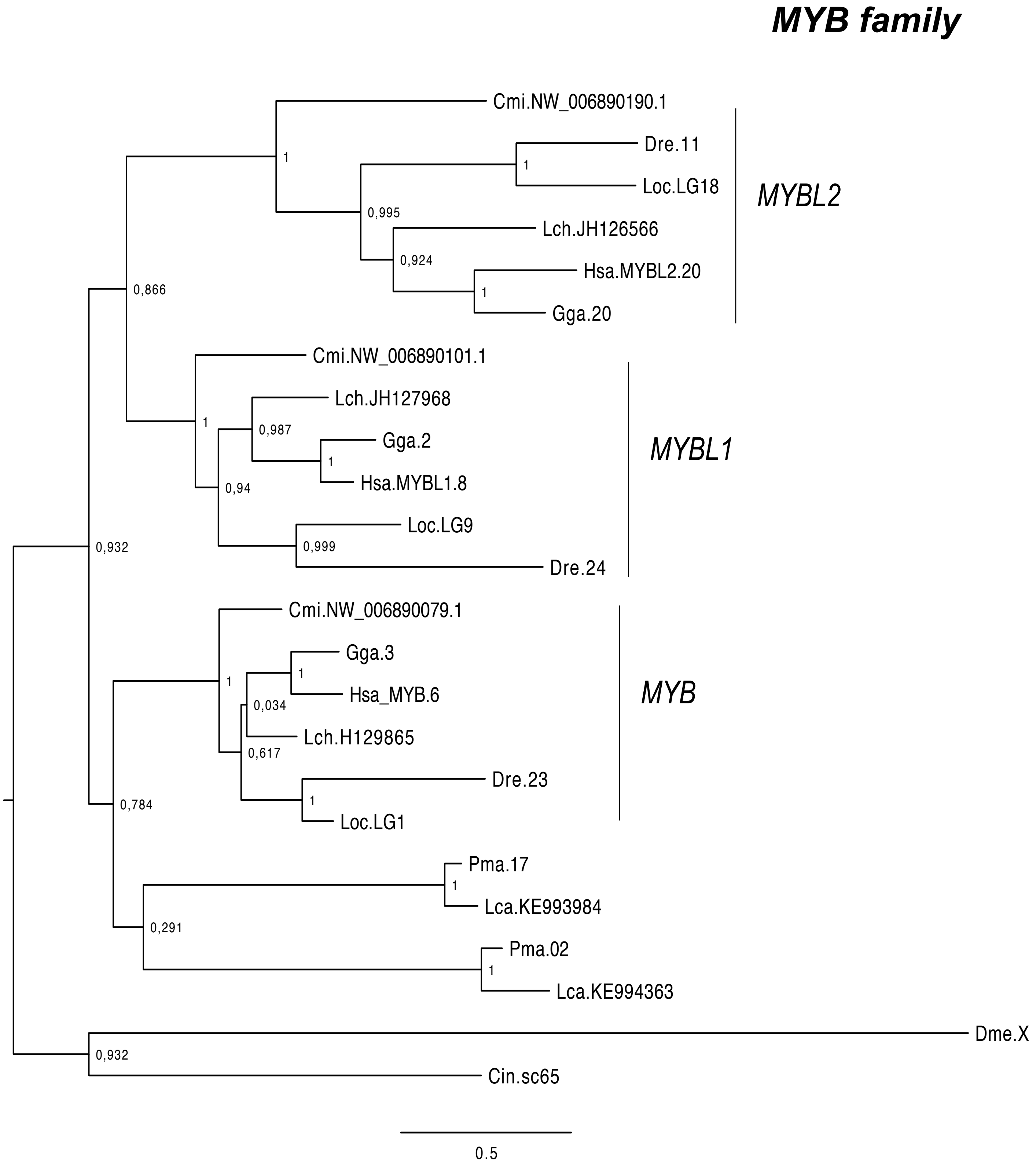
MYB family
Cmi.NW_006890190.1
Dre.11
Loc.LG18
Lch.JH126566
Hsa.MYBL2.20
Gga.20
MYBL2
Cmi.NW_006890101.1
Lch.JH127968
Gga.2
Hsa.MYBL1.8
Loc.LG9
Dre.24
MYBL1
Cmi.NW_006890079.1
Gga.3
Hsa_MYB.6
Lch.H129865
Dre.23
Loc.LG1
MYB
Pma.17
Lca.KE993984
Pma.02
Lca.KE994363
Dme.X
Cin.sc65
1
1
0,995
0,924
1
0,866
1
0,987
1
0,94
0,999
0,932
1
1
0,034
0,617
1
0,784
1
0,291
1
0,932
0.5

NCOA family

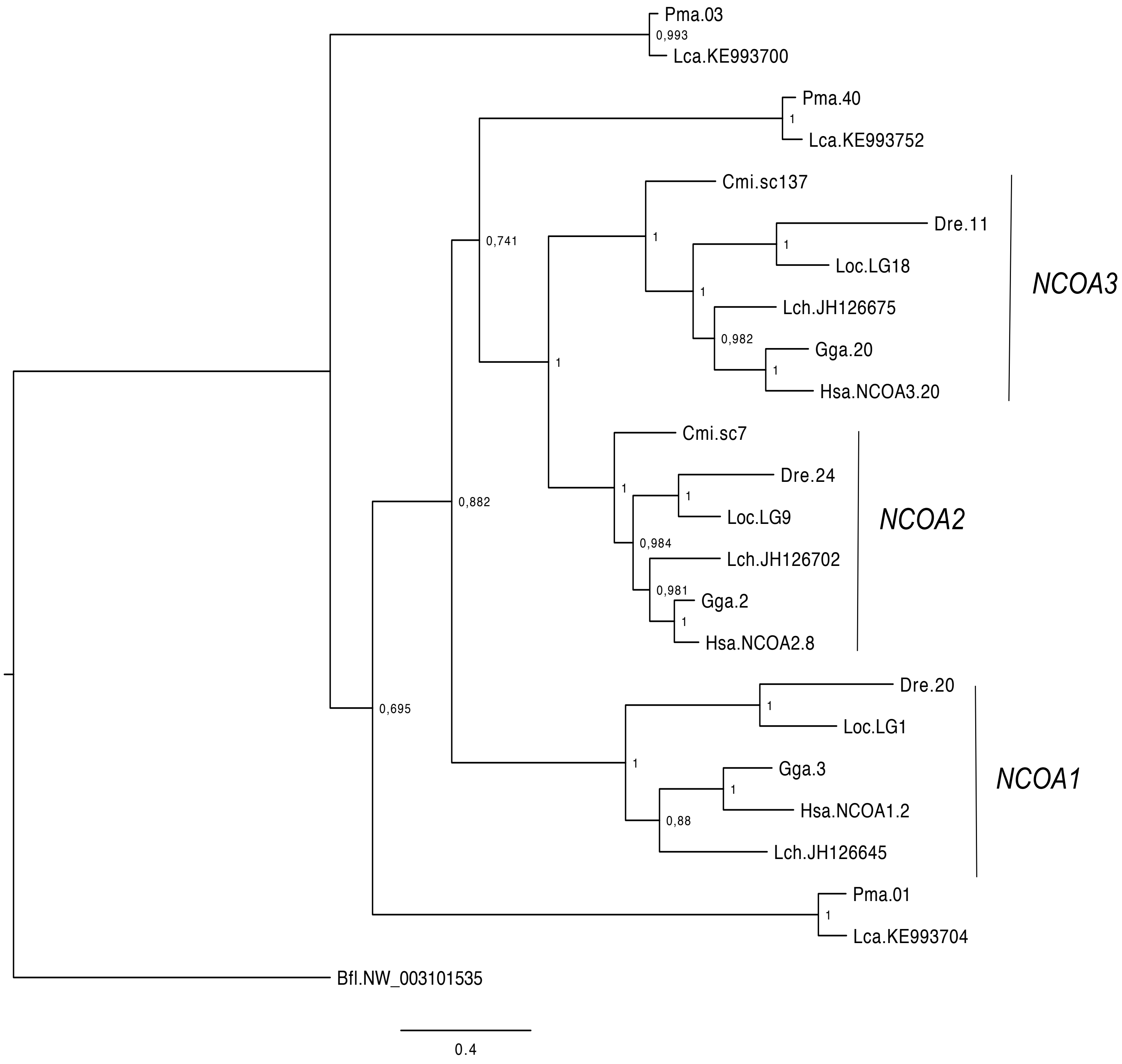
Pma.03
0,993
Lca.KE993700
Pma.40
1
Lca.KE993752
Cmi.sc137
Dre.11
1
Loc.LG18
0,741
1
1
Lch.JH126675
0,982
Gga.20
1
1
Hsa.NCOA3.20
NCOA3
Cmi.sc7
Dre.24
1
1
0,882
Loc.LG9
0,984
Lch.JH126702
NCOA2
0,981
Gga.2
1
Hsa.NCOA2.8
Dre.20
1
0,695
Loc.LG1
1
Gga.3
1
Hsa.NCOA1.2
NCOA1
0,88
Lch.JH126645
Pma.01
1
Lca.KE993704
Bfl.NW_003101535
0.4

EPB41 family

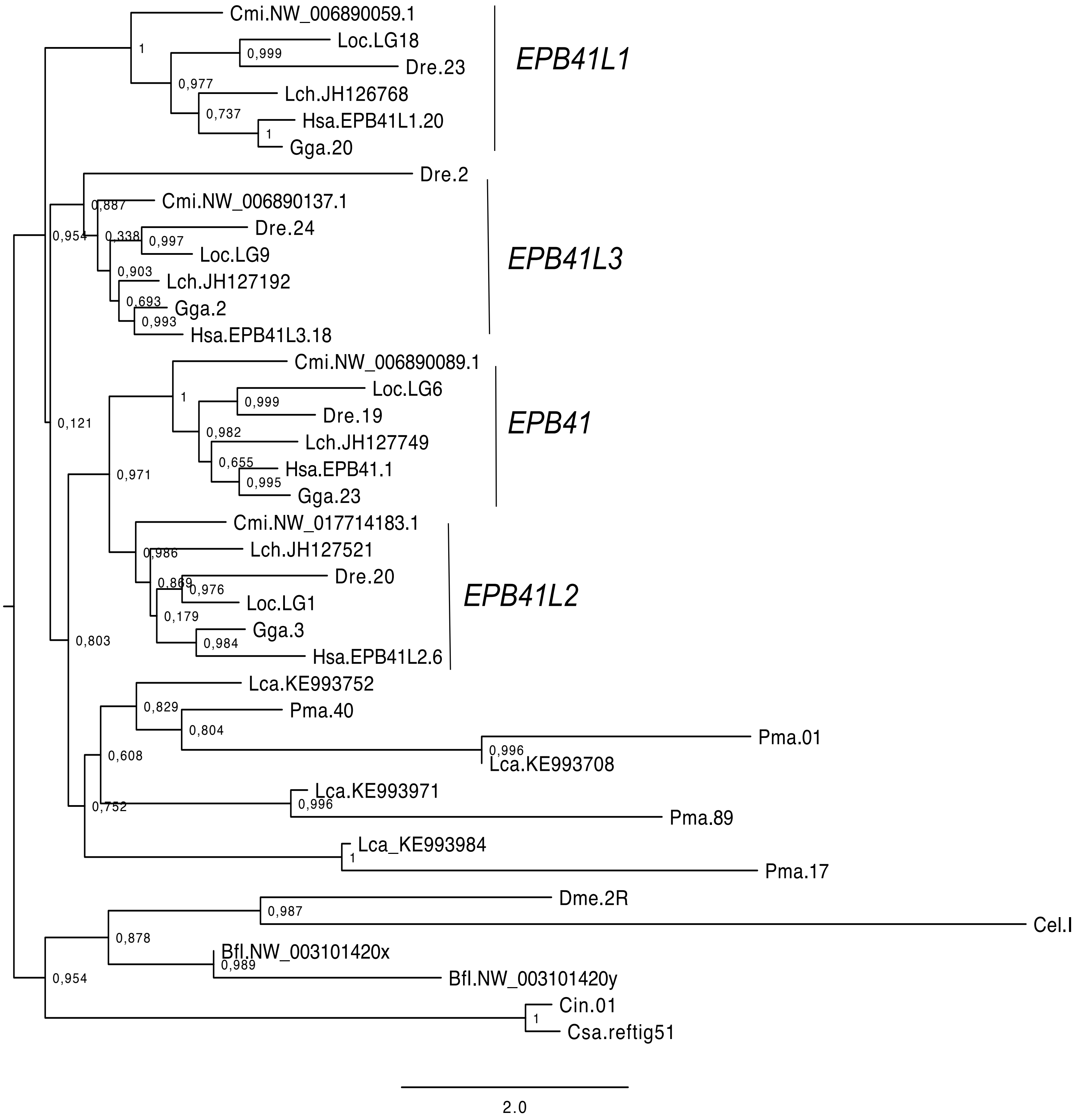

GATA family

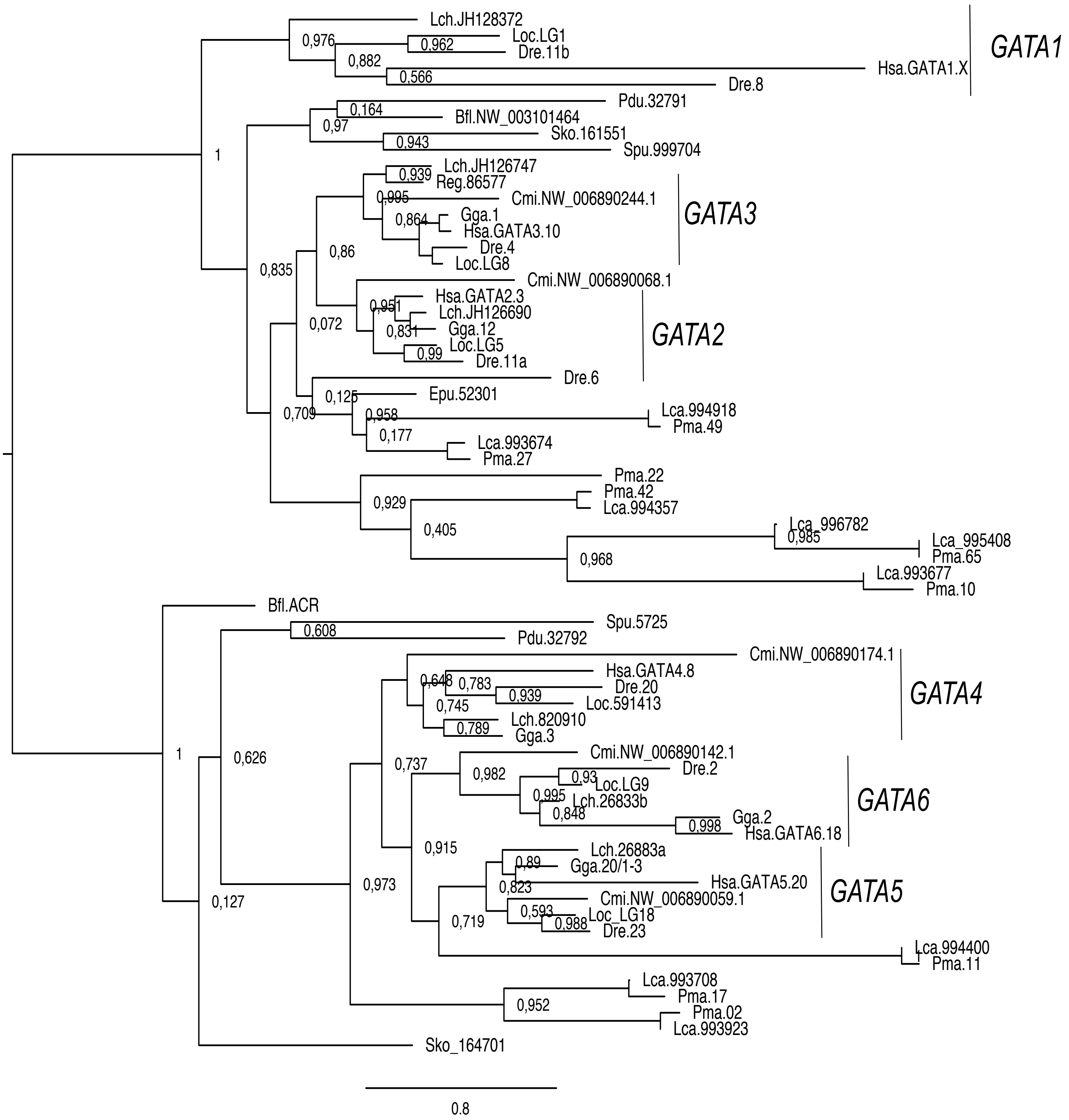
GATA1
GATA3
GATA2
GATA4
GATA6
GATA5
Lch.JH128372
Loc.LG1
Dre.11b
Hsa.GATA1.X
Dre.8
Pdu.32791
Bfl.NW_003101464
Sko.161551
Spu.999704
Lch.JH126747
Reg.86577
Cmi.NW_006890244.1
Gga.1
Hsa.GATA3.10
Dre.4
Loc.LG8
Cmi.NW_006890068.1
Hsa.GATA2.3
Lch.JH126690
Gga.12
Loc.LG5
Dre.11a
Dre.6
Epu.52301
Lca.994918
Pma.49
Lca.993674
Pma.27
Pma.22
Pma.42
Lca.994357
Lca_996782
Lca_995408
Pma.65
Lca.993677
Pma.10
Bfl.ACR
Spu.5725
Pdu.32792
Cmi.NW_006890174.1
Hsa.GATA4.8
Dre.20
Loc.591413
Lch.820910
Gga.3
Cmi.NW_006890142.1
Dre.2
Loc.LG9
Lch.26833b
Gga.2
Hsa.GATA6.18
Lch.26883a
Gga.20/1-3
Hsa.GATA5.20
Cmi.NW_006890059.1
Loc_LG18
Dre.23
Lca.994400
Pma.11
Lca.993708
Pma.17
Pma.02
Lca.993923
Sko_164701
0.8

STMN family

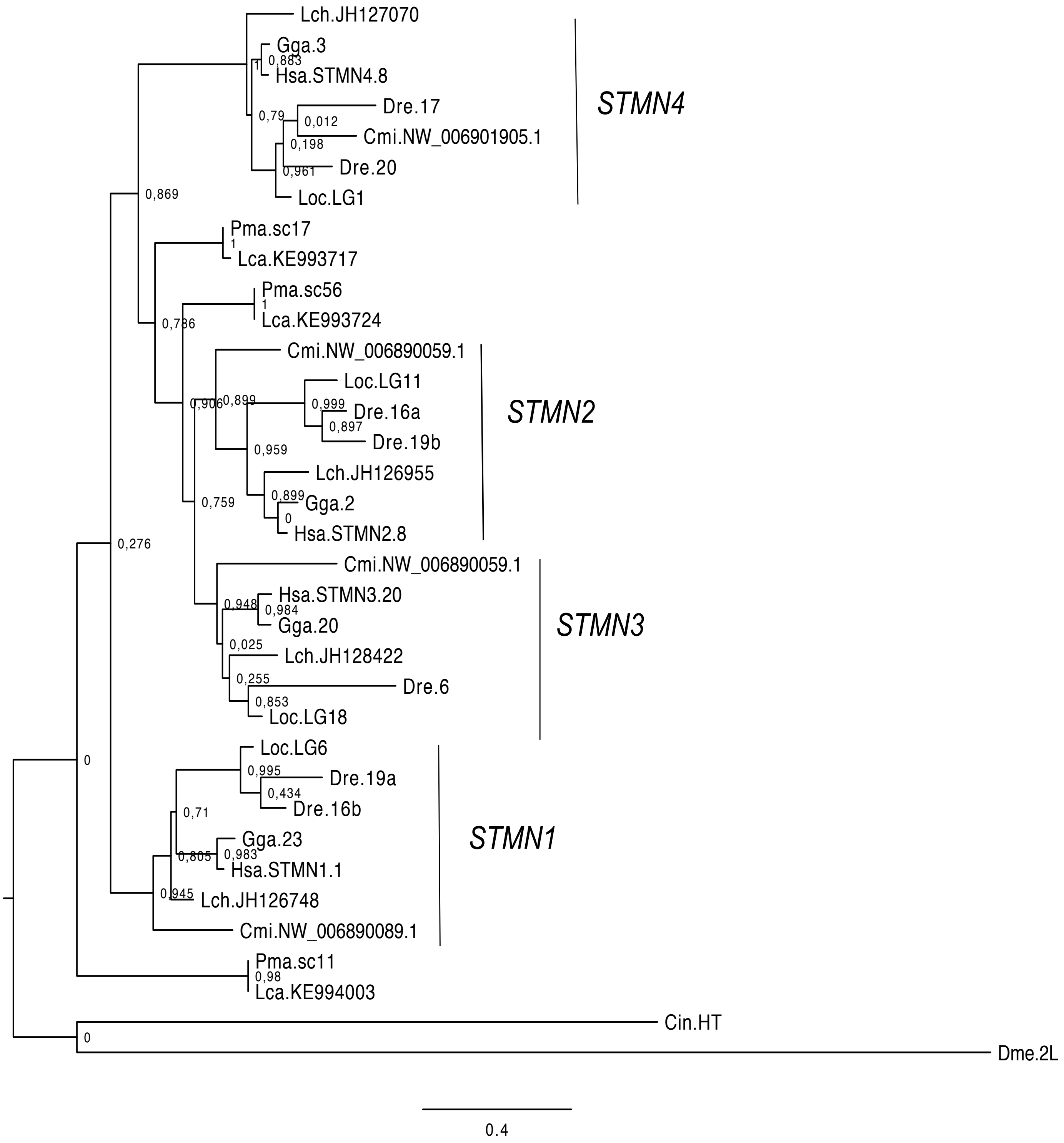

Lch.JH127070
Gga.3
0,883
1
Hsa.STMN4.8
Dre.17
0,79
0,012
Cmi.NW_006901905.1
0,198
Dre.20
0,961
Loc.LG1
0,869
STMN4
Pma.sc17
1
Lca.KE993717
Pma.sc56
1
Lca.KE993724
0,736
Cmi.NW_006890059.1
Loc.LG11
0,906
0,899
0,999
Dre.16a
0,897
Dre.19b
0,959
Lch.JH126955
0,899
Gga.2
0
Hsa.STMN2.8
0,759
STMN2
0,276
Cmi.NW_006890059.1
Hsa.STMN3.20
0,948
0,984
Gga.20
0,025
Lch.JH128422
0,255
Dre.6
0,853
Loc.LG18
STMN3
Loc.LG6
0
0,995
Dre.19a
0,434
Dre.16b
0,71
Gga.23
0,805
0,983
Hsa.STMN1.1
0,945
Lch.JH126748
Cmi.NW_006890089.1
STMN1
Pma.sc11
0,98
Lca.KE994003
Cin.HT
0
Dme.2L
0.4

DLGAP family

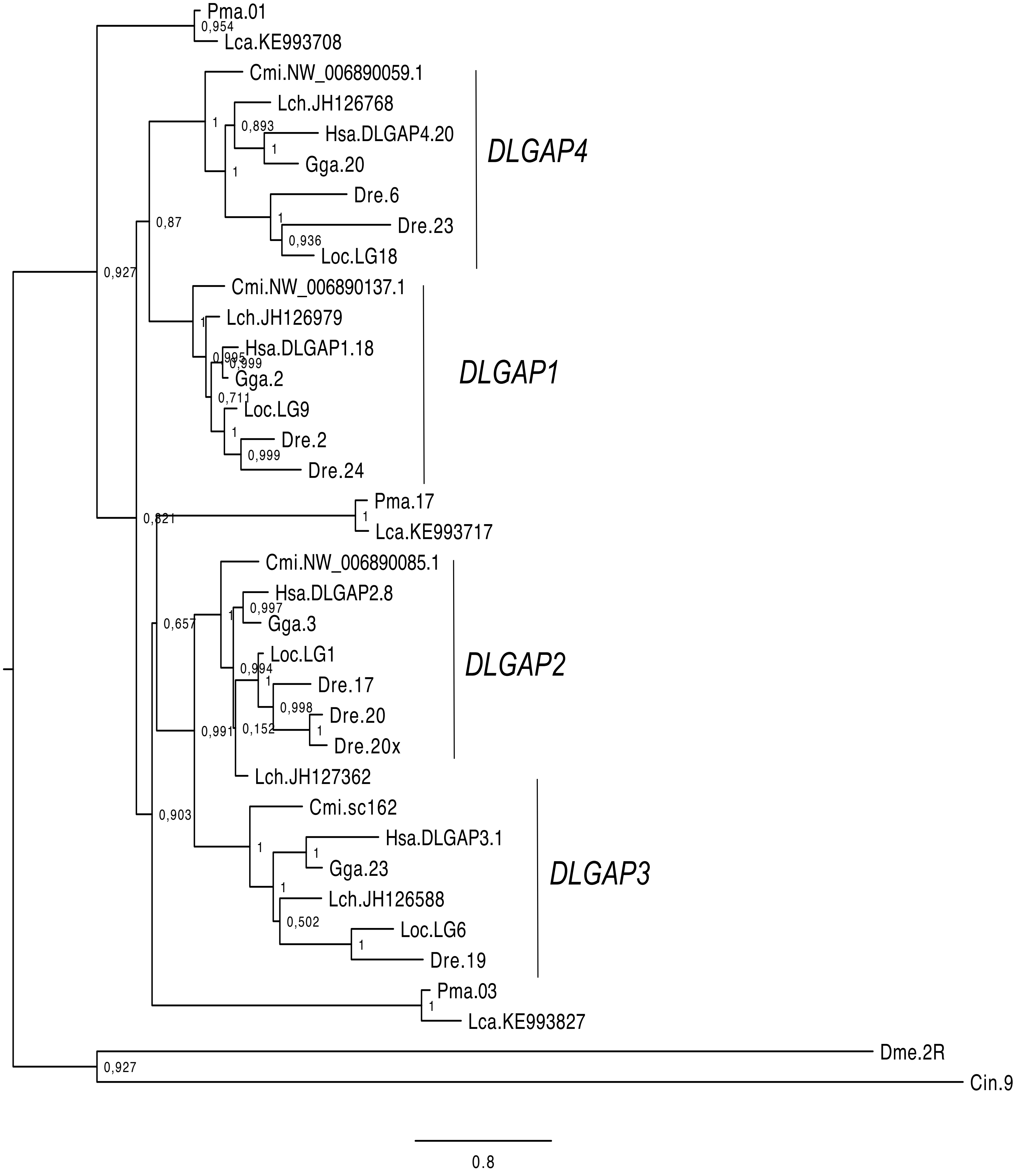

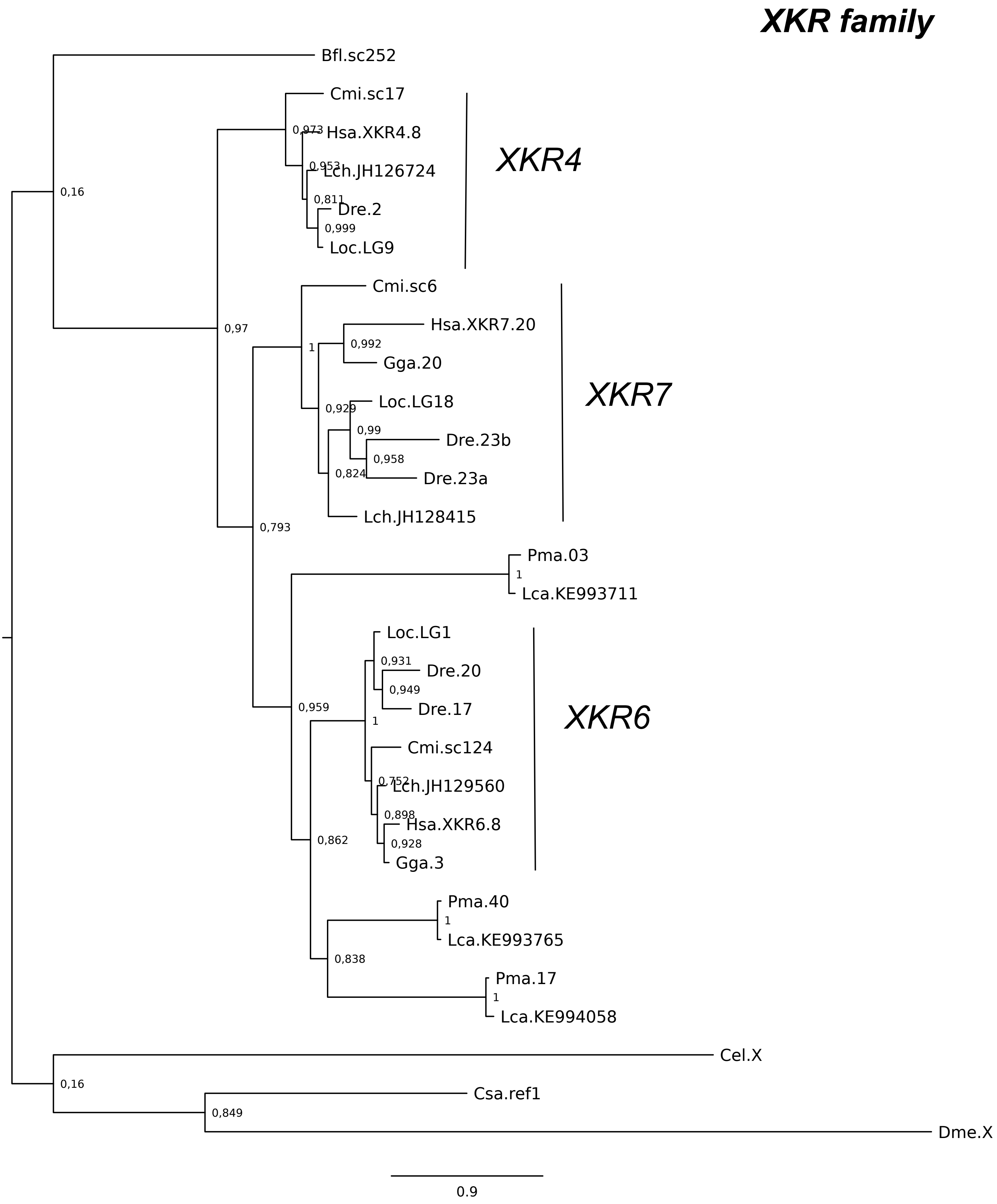

XKR family
Bfl.sc252
Cmi.sc17
0,973
Hsa.XKR4.8
0,953
Lch.JH126724
0,811
Dre.2
0,999
Loc.LG9
XKR4
0,16
Cmi.sc6
Hsa.XKR7.20
0,992
Gga.20
1
0,97
Loc.LG18
0,929
0,99
Dre.23b
0,958
Dre.23a
0,824
Lch.JH128415
XKR7
0,793
Pma.03
1
Lca.KE993711
Loc.LG1
0,931
Dre.20
0,949
Dre.17
1
Cmi.sc124
0,752
Lch.JH129560
0,898
Hsa.XKR6.8
0,928
Gga.3
XKR6
0,959
0,862
Pma.40
1
Lca.KE993765
0,838
Pma.17
1
Lca.KE994058
Cel.X
0,16
Csa.ref1
0,849
Dme.X
0.9
