# Supplementary material for: Corticotropin-Releasing Hormone (CRH) Gene Family Duplications in Lampreys Correlate With Two Early Vertebrate Genome Doublings
Source: Front Neurosci. 2020 Jul 30;14:672. doi: 10.3389/fnins.2020.00672 (PMC7406891; doi:10.3389/fnins.2020.00672)
Supplement: FIGURE S1 — Bayesian inference tree of the CRH mature peptides. Branch support values (posterior probability values) are shown and tree was rooted with the tunicate CRH-like precursors. Tree was rooted with the tunicate CRH-family members. A simplified radial tree is represented in Figure 1. Accession numbers of the sequences used are available in Supplementary Table S2. [file Data_Sheet_1.zip › Figure S6.PDF]

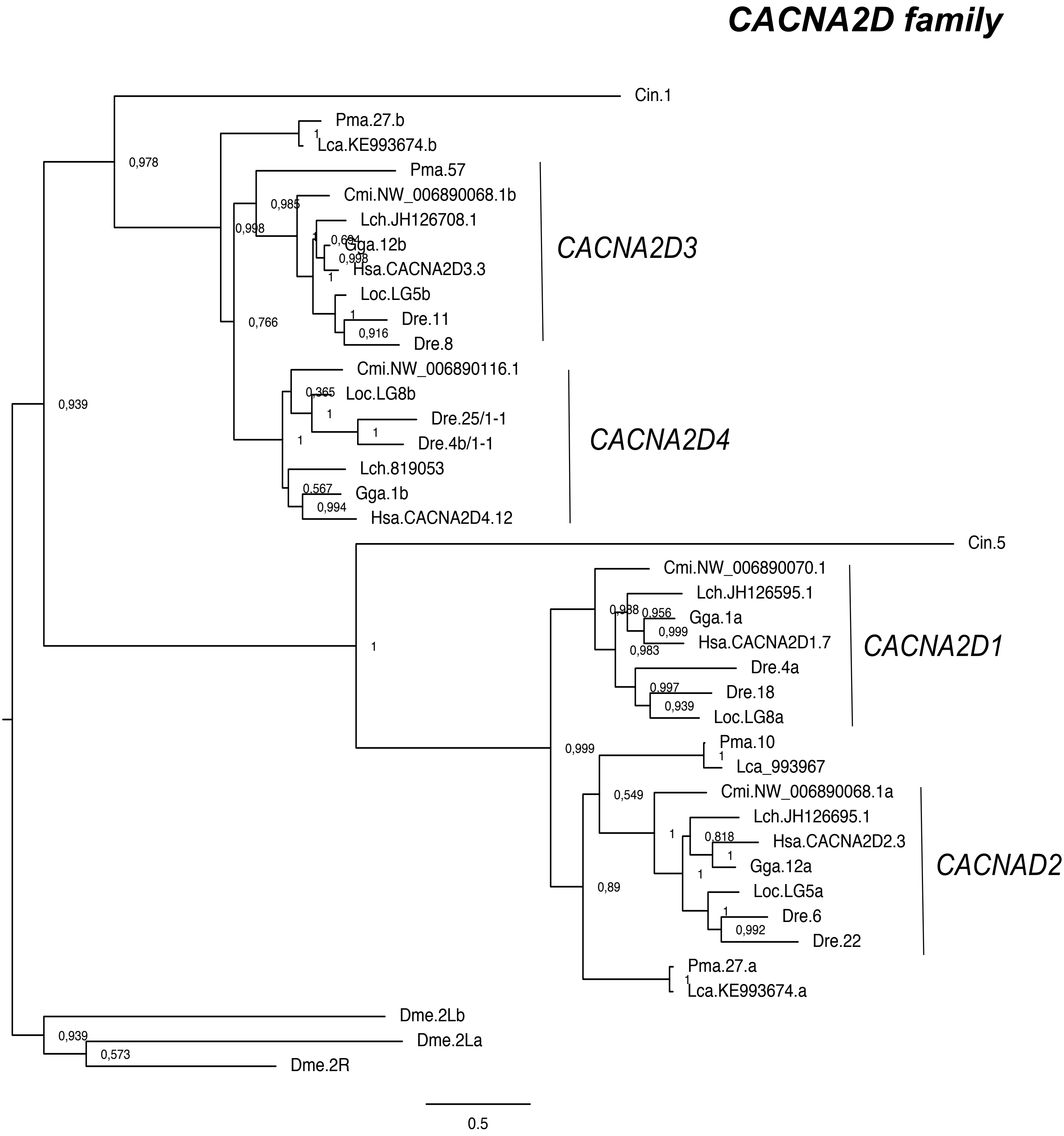
CACNA2D family
Cin.1
Pma.27.b
Lca.KE993674.b
Pma.57
Cmi.NW_006890068.1b
Lch.JH126708.1
Gga.12b
Hsa.CACNA2D3.3
Loc.LG5b
Dre.11
Dre.8
CACNA2D3
Cmi.NW_006890116.1
Loc.LG8b
Dre.25/1-1
Dre.4b/1-1
Lch.819053
Gga.1b
Hsa.CACNA2D4.12
CACNA2D4
Cin.5
Cmi.NW_006890070.1
Lch.JH126595.1
Gga.1a
Hsa.CACNA2D1.7
Dre.4a
Dre.18
Loc.LG8a
CACNA2D1
Pma.10
Lca_993967
Cmi.NW_006890068.1a
Lch.JH126695.1
Hsa.CACNA2D2.3
Gga.12a
Loc.LG5a
Dre.6
Dre.22
CACNAD2
Pma.27.a
Lca.KE993674.a
Dme.2Lb
Dme.2La
Dme.2R
0,978
0,985
0,998
0,694
0,998
0,766
0,916
0,939
0,365
0,567
0,994
0,988
0,956
0,999
0,983
0,997
0,939
0,999
0,549
0,818
0,89
0,992
0,939
0,573
0.5

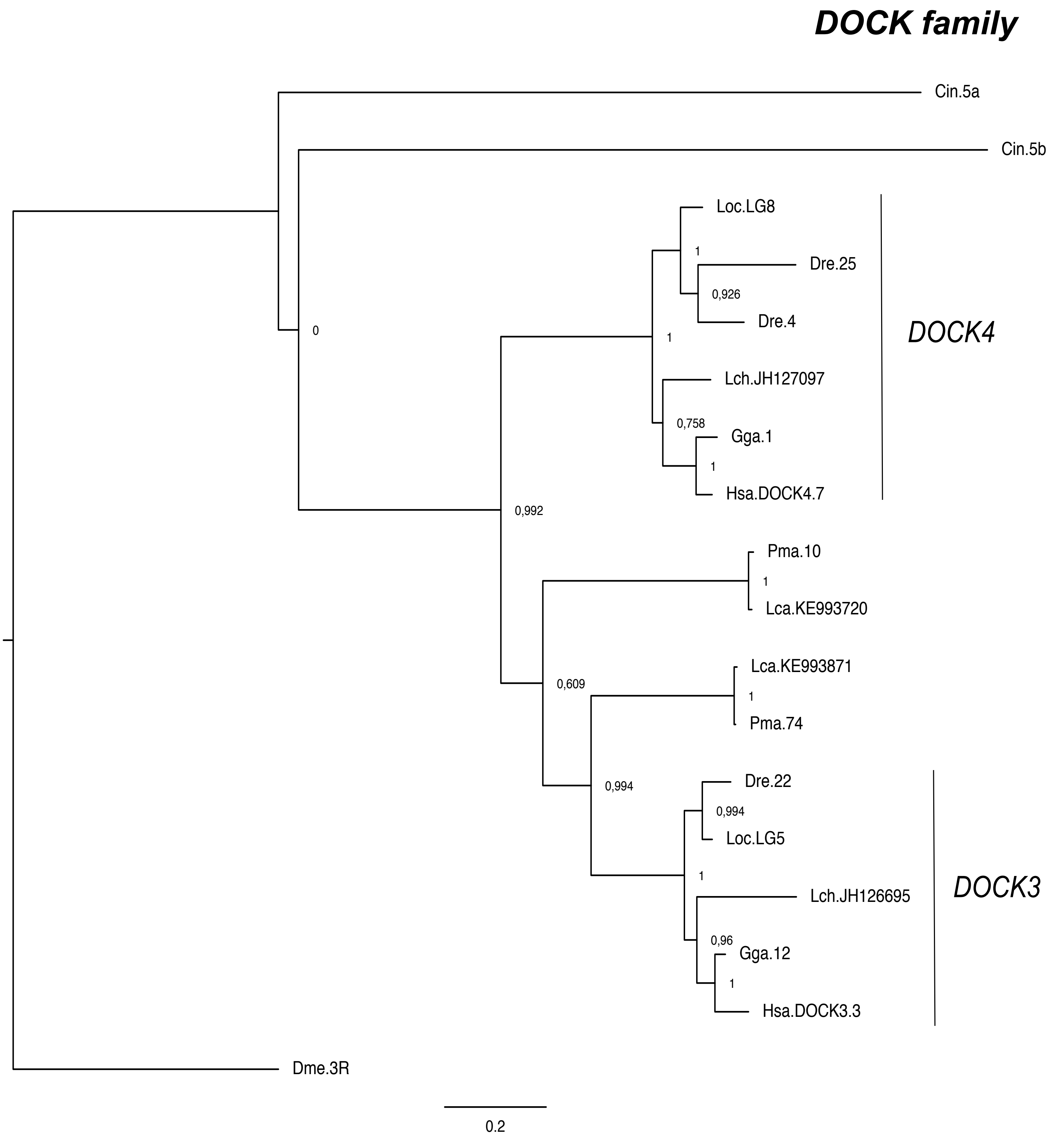
DOCK family
Cin.5a
Cin.5b
Loc.LG8
1
Dre.25
0,926
Dre.4
DOCK4
0
1
Lch.JH127097
0,758
Gga.1
1
Hsa.DOCK4.7
0,992
Pma.10
1
Lca.KE993720
Lca.KE993871
0,609
1
Pma.74
Dre.22
0,994
0,994
Loc.LG5
1
Lch.JH126695
DOCK3
0,96
Gga.12
1
Hsa.DOCK3.3
Dme.3R
0.2

MAPKAPK family

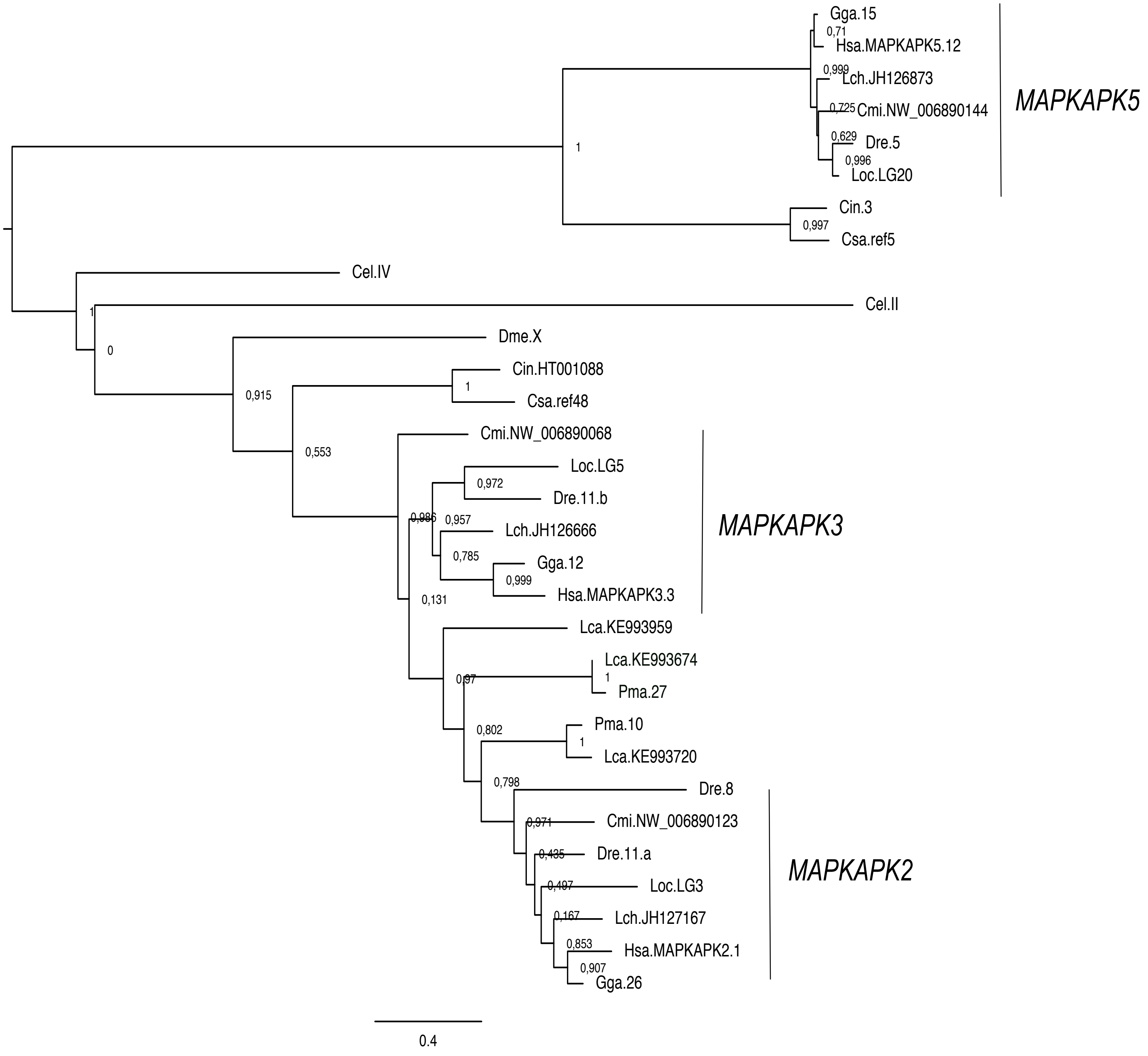

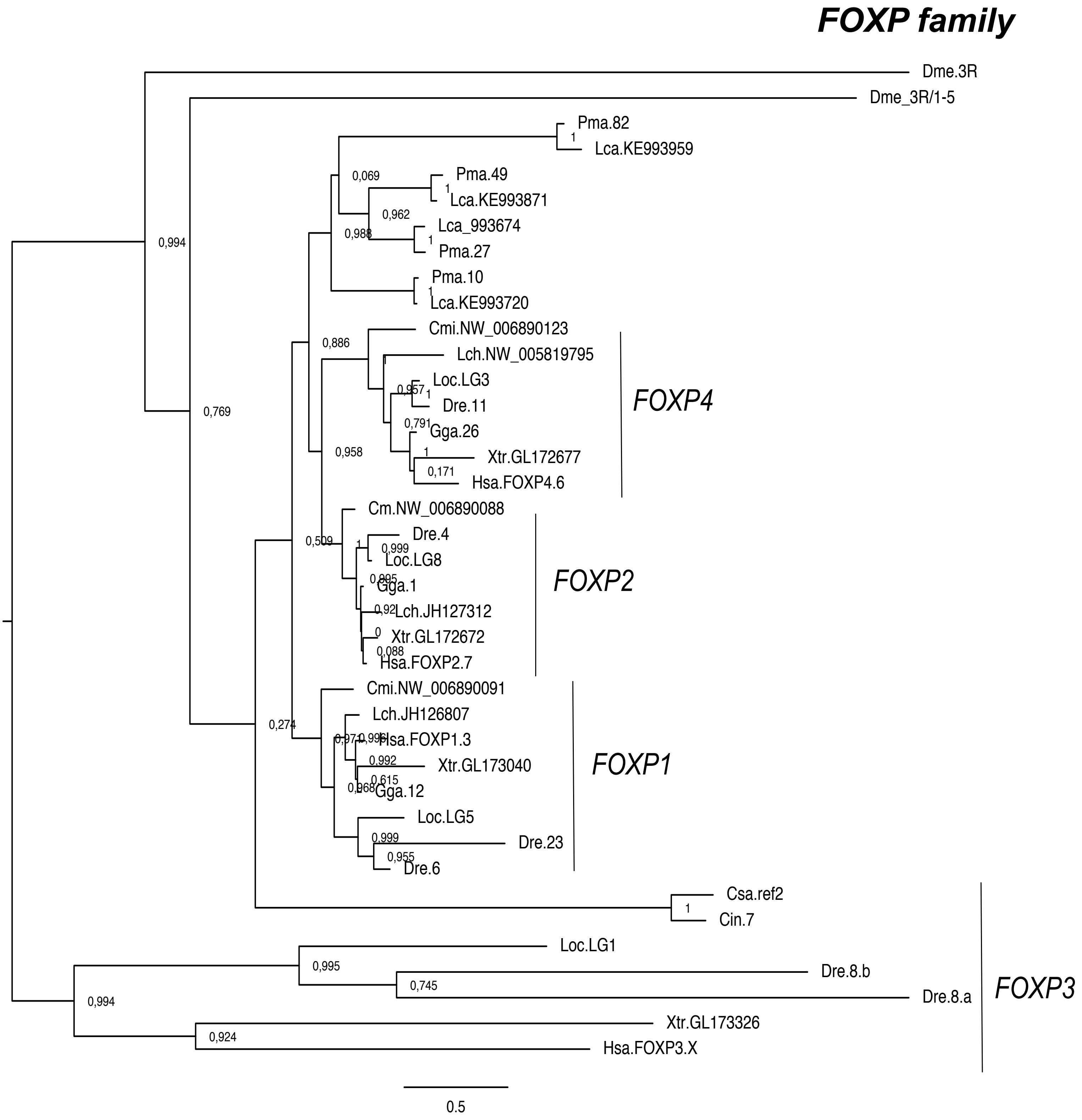
FOXP family
Dme.3R
Dme_3R/1-5
Pma.82
Lca.KE993959
Pma.49
Lca.KE993871
Lca_993674
Pma.27
Pma.10
Lca.KE993720
Cmi.NW_006890123
Lch.NW_005819795
Loc.LG3
Dre.11
Gga.26
Xtr.GL172677
Hsa.FOXP4.6
FOXP4
Cm.NW_006890088
Dre.4
Loc.LG8
Gga.1
Lch.JH127312
Xtr.GL172672
Hsa.FOXP2.7
FOXP2
Cmi.NW_006890091
Lch.JH126807
Hsa.FOXP1.3
Xtr.GL173040
Gga.12
Loc.LG5
Dre.23
Dre.6
FOXP1
Csa.ref2
Cin.7
Loc.LG1
Dre.8.b
Dre.8.a
Xtr.GL173326
Hsa.FOXP3.X
FOXP3
0,994
0,769
0,069
0,962
0,988
0,886
0,957
0,791
0,958
0,171
0,509
0,999
0,274
0,992
0,615
0,999
0,955
0,995
0,745
0,994
0,924
0.5

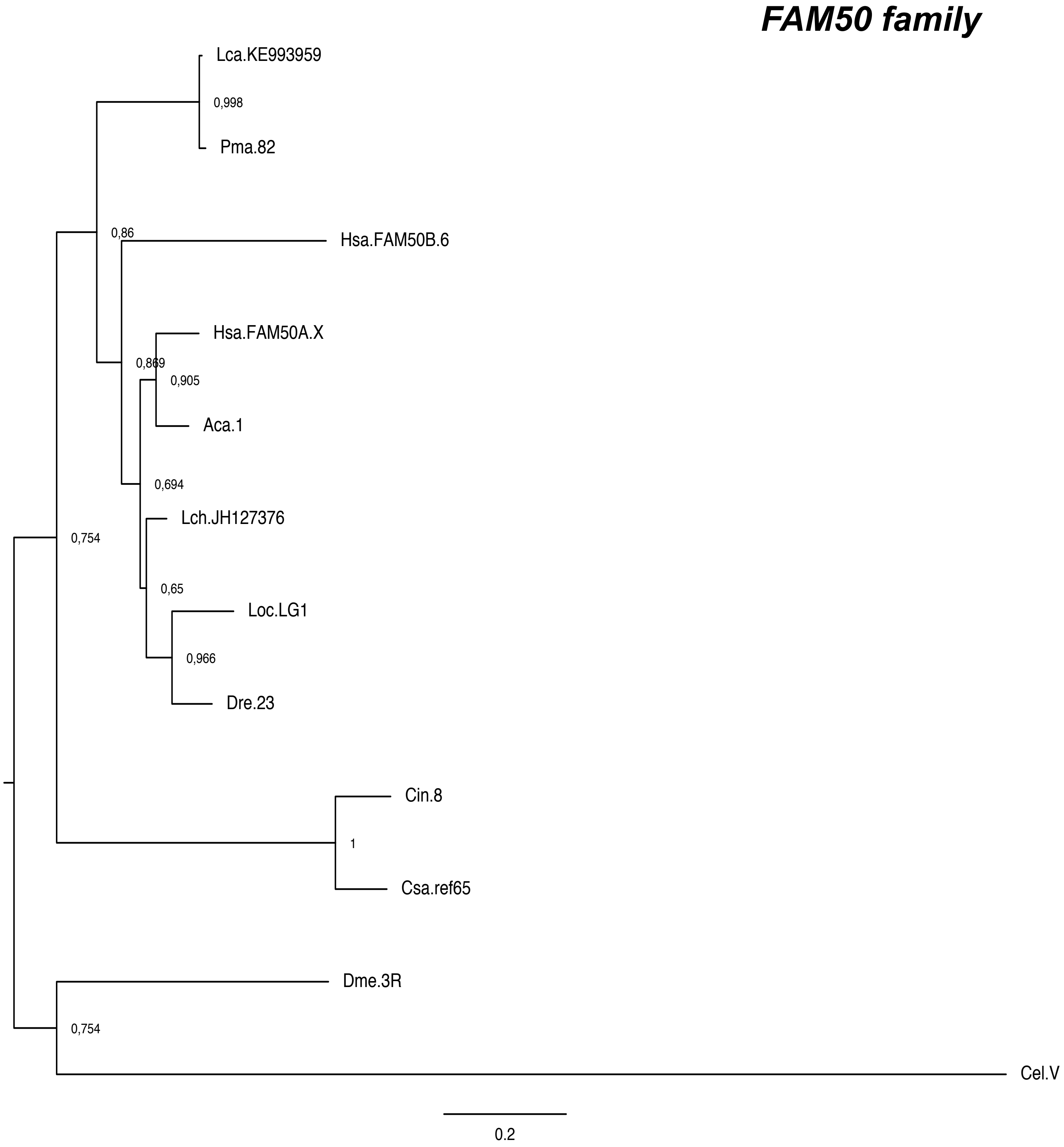
FAM50 family
Lca.KE993959
0,998
Pma.82
0,86
Hsa.FAM50B.6
Hsa.FAM50A.X
0,869
0,905
Aca.1
0,694
Lch.JH127376
0,754
0,65
Loc.LG1
0,966
Dre.23
Cin.8
1
Csa.ref65
Dme.3R
0,754
Cel.V
0.2

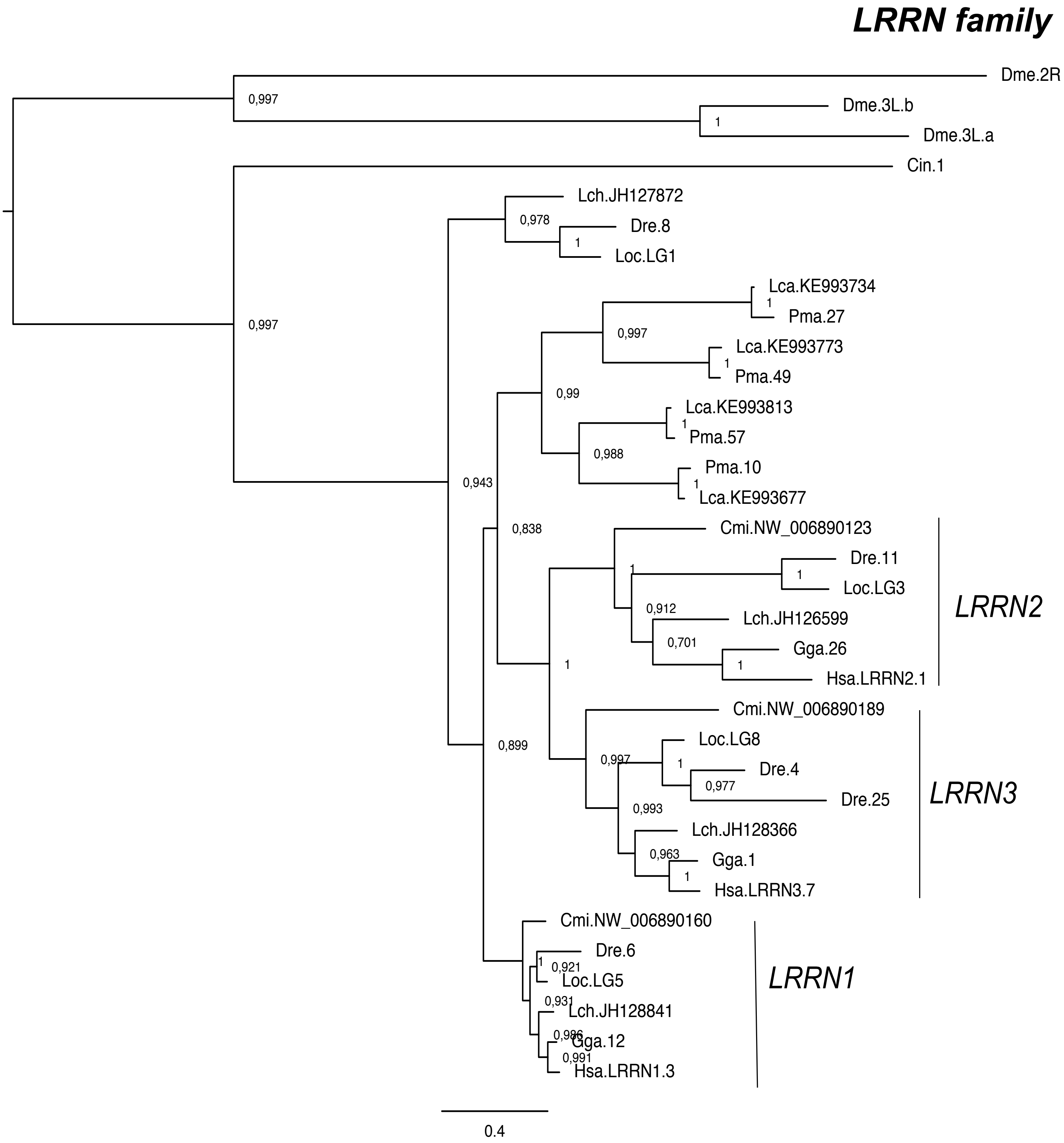

LRRN family
Dme.2R
Dme.3L.b
Dme.3L.a
Cin.1
Lch.JH127872
Dre.8
Loc.LG1
Lca.KE993734
Pma.27
Lca.KE993773
Pma.49
Lca.KE993813
Pma.57
Pma.10
Lca.KE993677
Cmi.NW_006890123
Dre.11
Loc.LG3
Lch.JH126599
Gga.26
Hsa.LRRN2.1
LRRN2
Cmi.NW_006890189
Loc.LG8
Dre.4
Dre.25
Lch.JH128366
Gga.1
Hsa.LRRN3.7
LRRN3
Cmi.NW_006890160
Dre.6
Loc.LG5
Lch.JH128841
Gga.12
Hsa.LRRN1.3
LRRN1
0.4

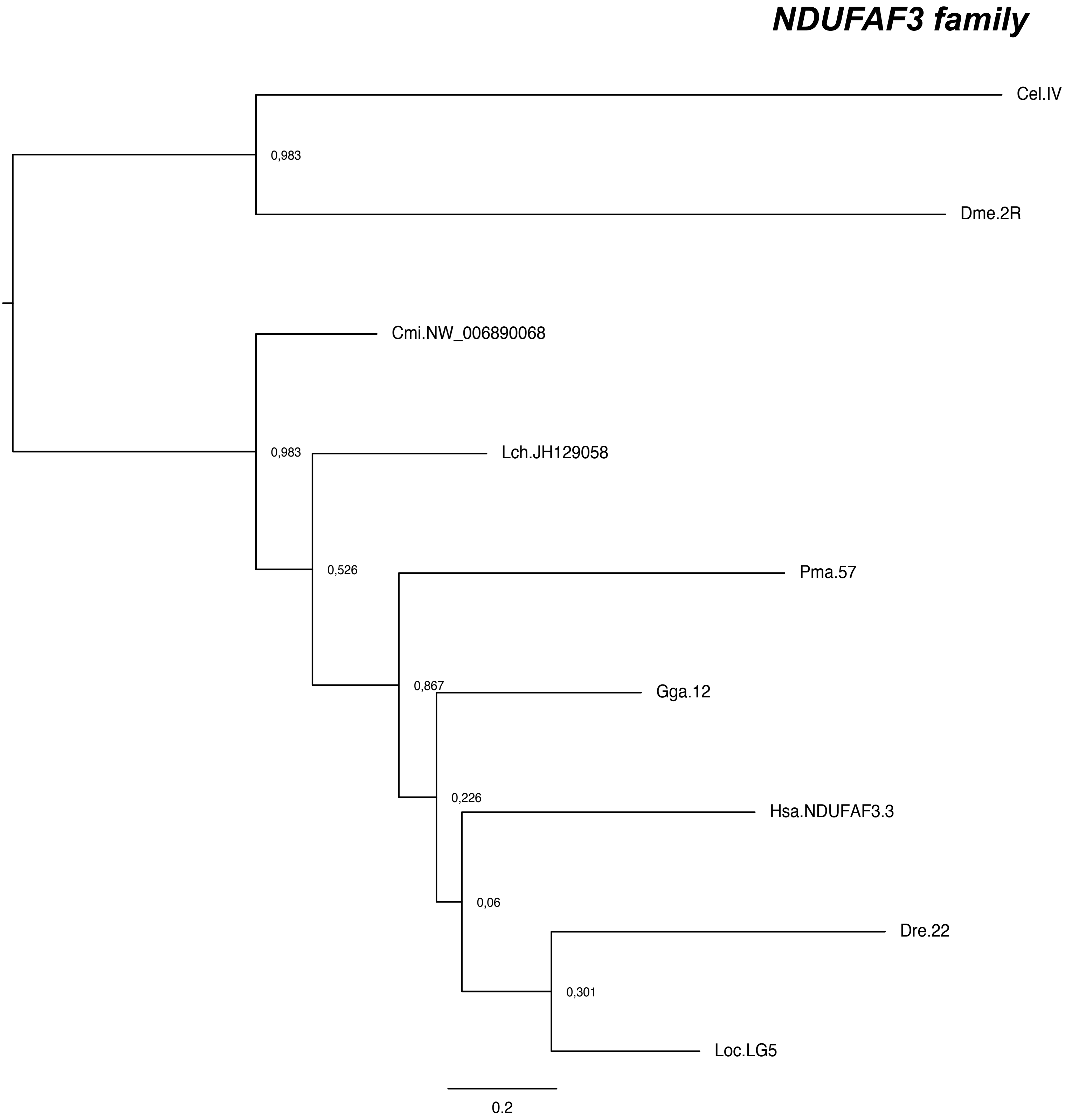
NDUFAF3 family
Cel.IV
0,983
Dme.2R
Cmi.NW_006890068
0,983
Lch.JH129058
0,526
Pma.57
0,867
Gga.12
0,226
Hsa.NDUFAF3.3
0,06
Dre.22
0,301
Loc.LG5
0.2

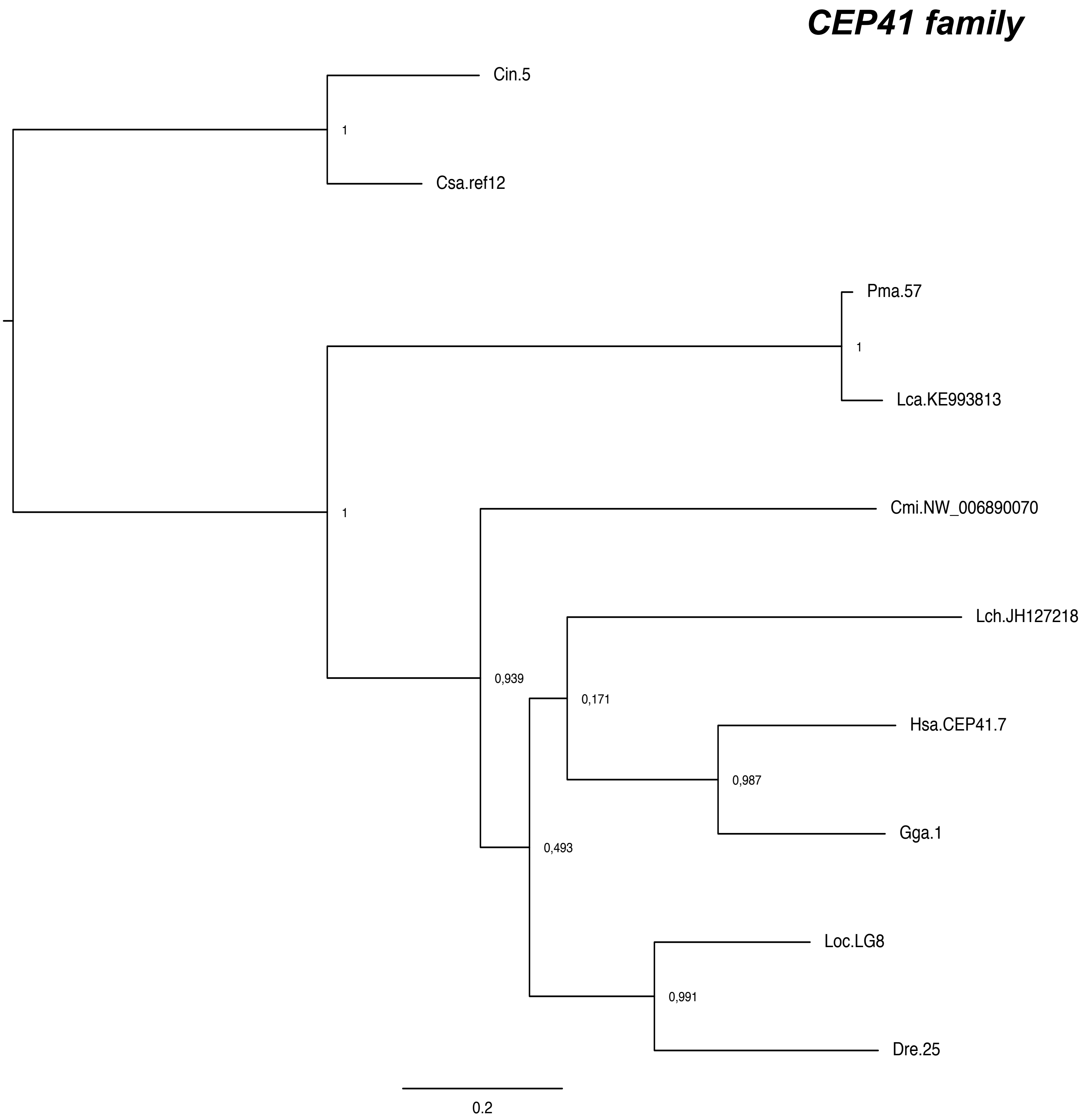
CEP41 family
Cin.5
1
Csa.ref12
Pma.57
1
Lca.KE993813
1
Cmi.NW_006890070
Lch.JH127218
0,939
0,171
Hsa.CEP41.7
0,987
Gga.1
0,493
Loc.LG8
0,991
Dre.25
0.2

MEST family

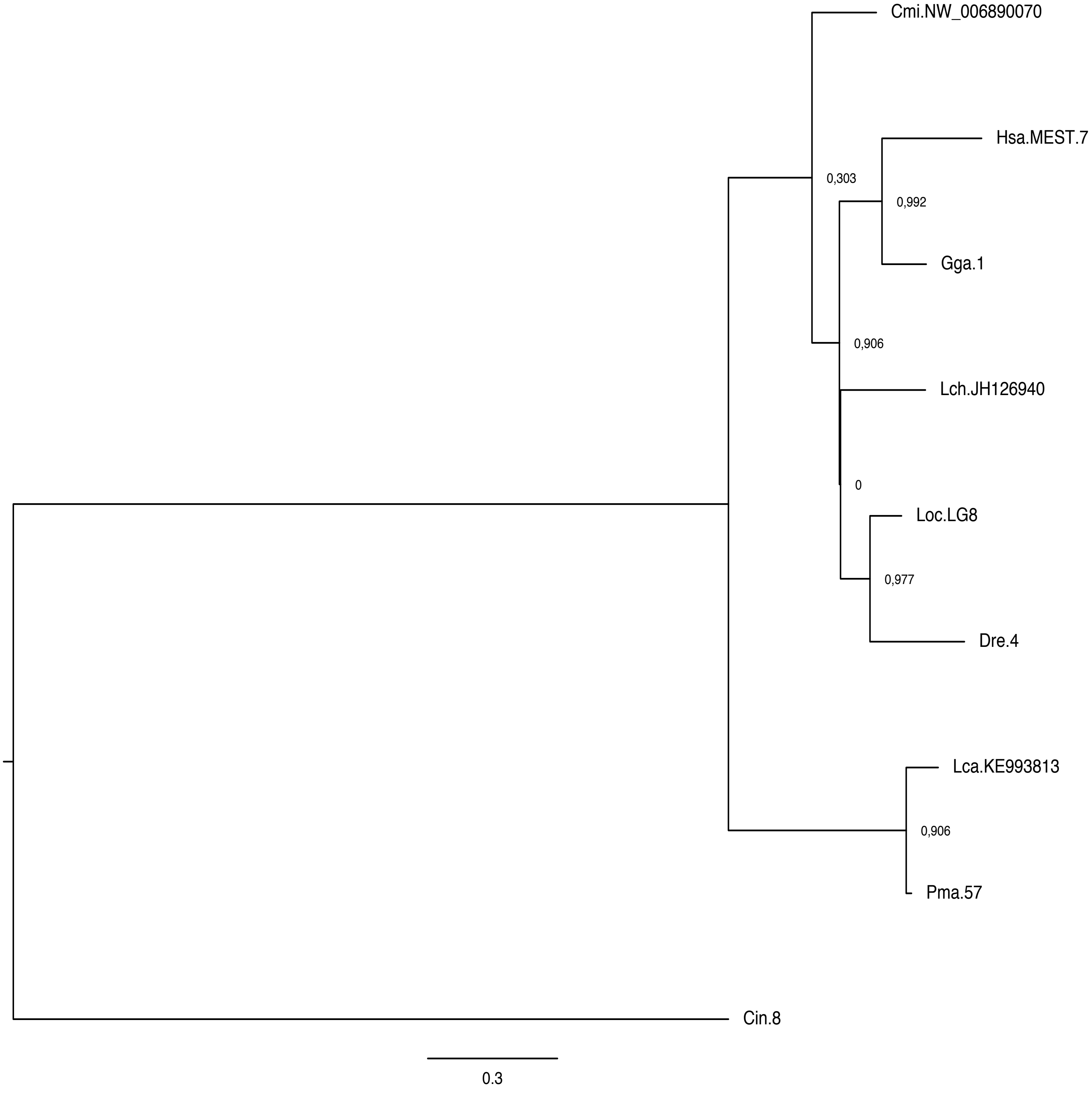
Cmi.NW_006890070
Hsa.MEST.7
0,303
0,992
Gga.1
0,906
Lch.JH126940
0
Loc.LG8
0,977
Dre.4
Lca.KE993813
0,906
Pma.57
Cin.8
0.3

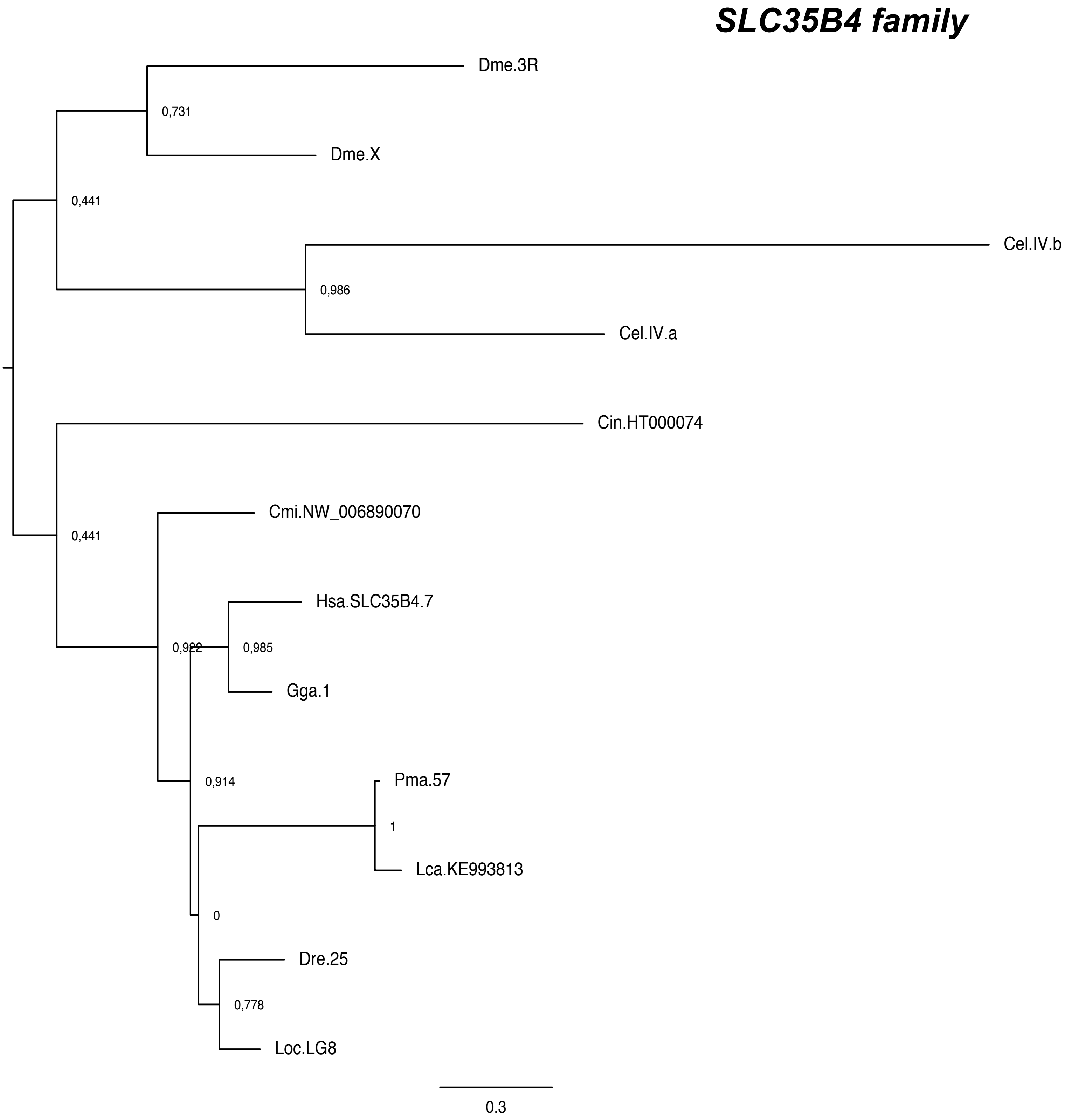
SLC35B4 family
Dme.3R
0,731
Dme.X
0,441
Cel.IV.b
0,986
Cel.IV.a
Cin.HT000074
Cmi.NW_006890070
0,441
Hsa.SLC35B4.7
0,922
0,985
Gga.1
0,914
Pma.57
1
Lca.KE993813
0
Dre.25
0,778
Loc.LG8
0.3

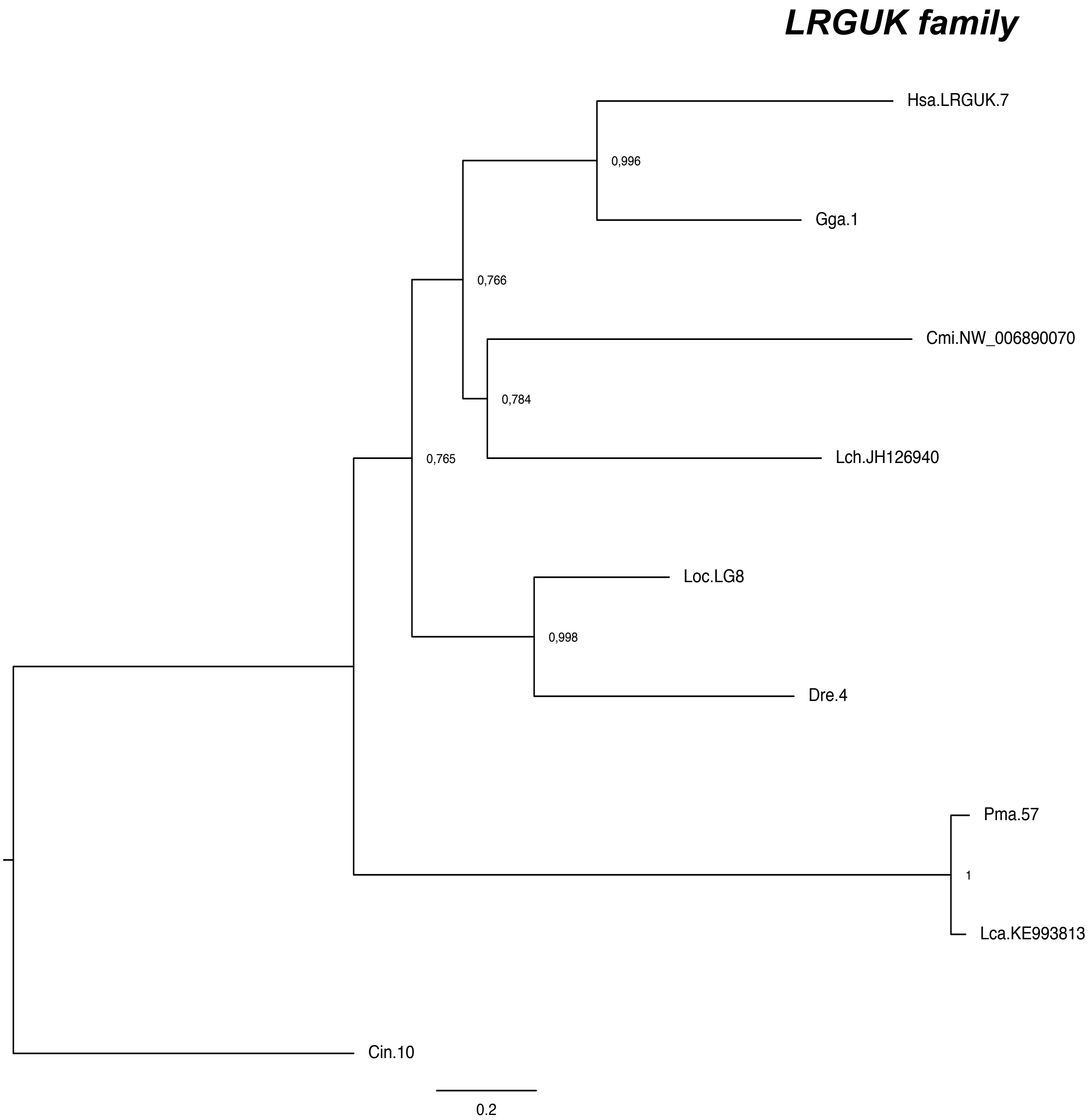
LRGUK family
Hsa.LRGUK.7
0,996
Gga.1
0,766
Cmi.NW_006890070
0,784
Lch.JH126940
0,765
Loc.LG8
0,998
Dre.4
Pma.57
1
Lca.KE993813
Cin.10
0.2

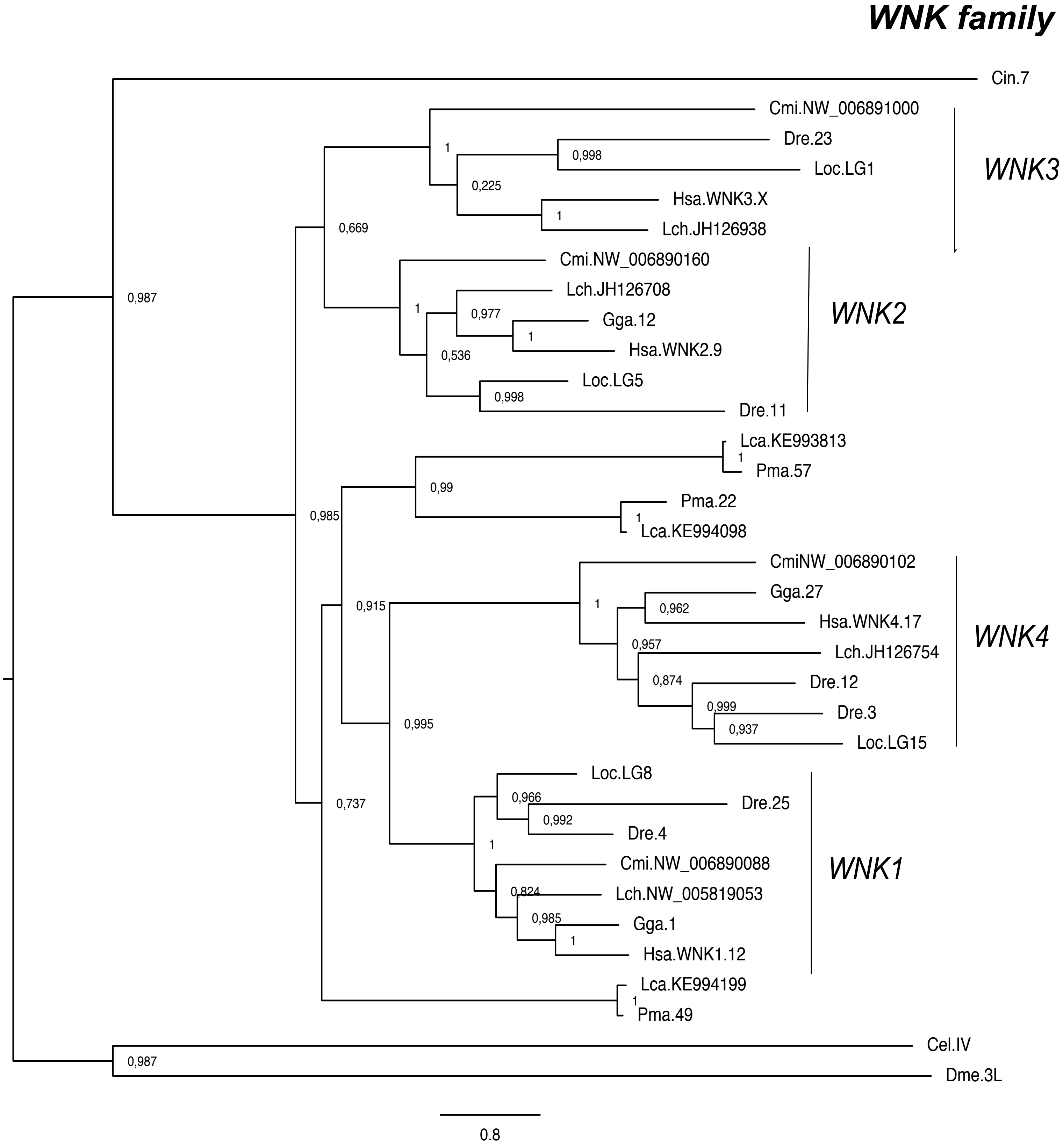
WNK family
Cin.7
Cmi.NW_006891000
Dre.23
Loc.LG1
Hsa.WNK3.X
Lch.JH126938
WNK3
Cmi.NW_006890160
Lch.JH126708
Gga.12
Hsa.WNK2.9
Loc.LG5
Dre.11
WNK2
Lca.KE993813
Pma.57
Pma.22
Lca.KE994098
CmiNW_006890102
Gga.27
Hsa.WNK4.17
Lch.JH126754
Dre.12
Dre.3
Loc.LG15
WNK4
Loc.LG8
Dre.25
Dre.4
Cmi.NW_006890088
Lch.NW_005819053
Gga.1
Hsa.WNK1.12
WNK1
Lca.KE994199
Pma.49
Cel.IV
Dme.3L
0,987
1
0,998
0,225
0,669
1
0,977
0,536
0,998
0,985
0,99
0,915
0,962
0,957
0,874
0,999
0,937
0,995
0,737
0,966
0,992
0,824
0,985
0.8

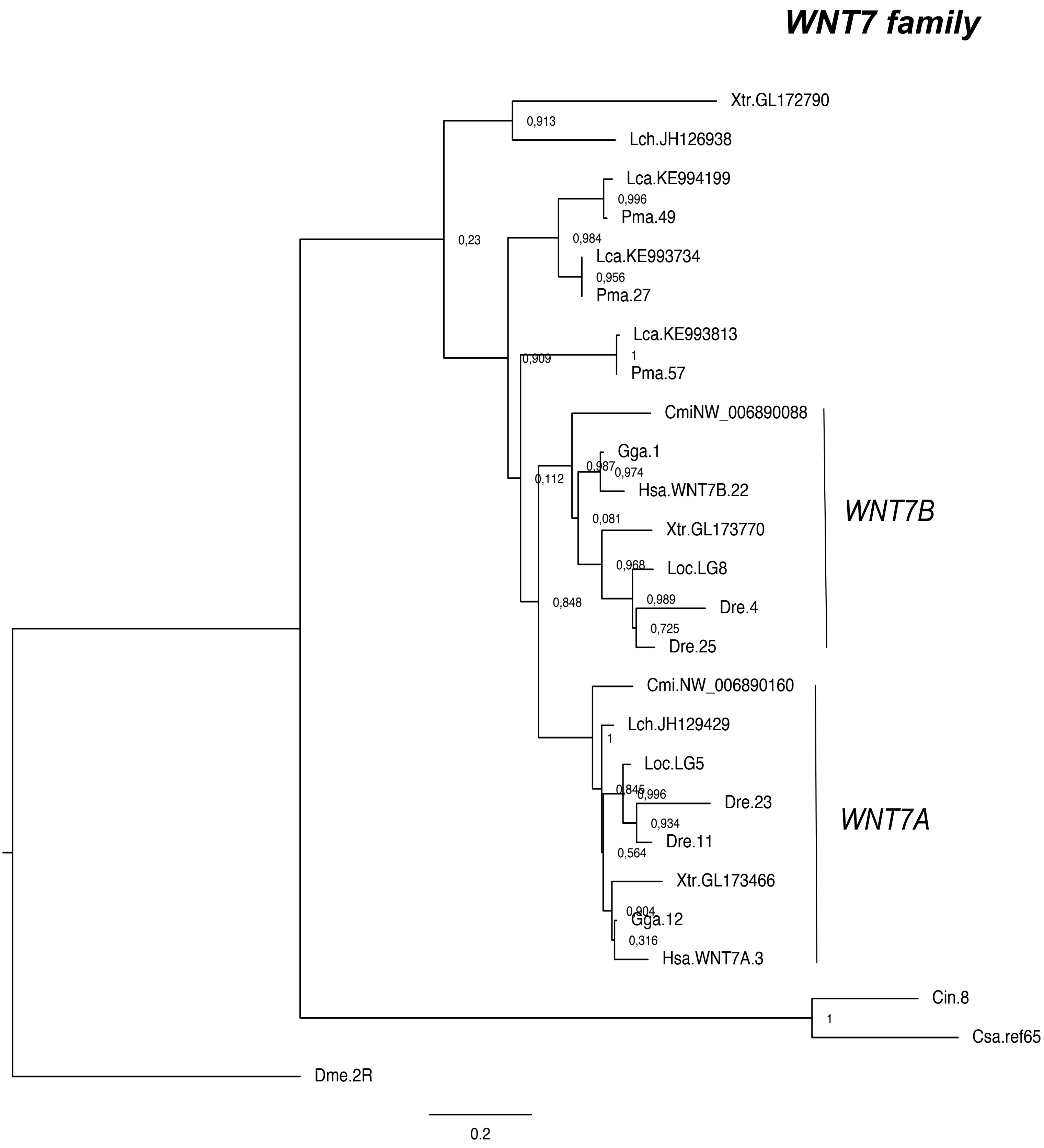
WNT7 family
Xtr.GL172790
0,913
Lch.JH126938
Lca.KE994199
0,996
Pma.49
0,23
0,984
Lca.KE993734
0,956
Pma.27
Lca.KE993813
1
0,909
Pma.57
CmiNW_006890088
Gga.1
0,987
0,974
0,112
Hsa.WNT7B.22
0,081
Xtr.GL173770
0,968
Loc.LG8
0,989
0,848
Dre.4
0,725
Dre.25
WNT7B
Cmi.NW_006890160
Lch.JH129429
1
Loc.LG5
0,845
0,996
Dre.23
0,934
Dre.11
WNT7A
0,564
Xtr.GL173466
0,904
Gga.12
0,316
Hsa.WNT7A.3
Cin.8
1
Csa.ref65
Dme.2R
0.2

TMEM243 family

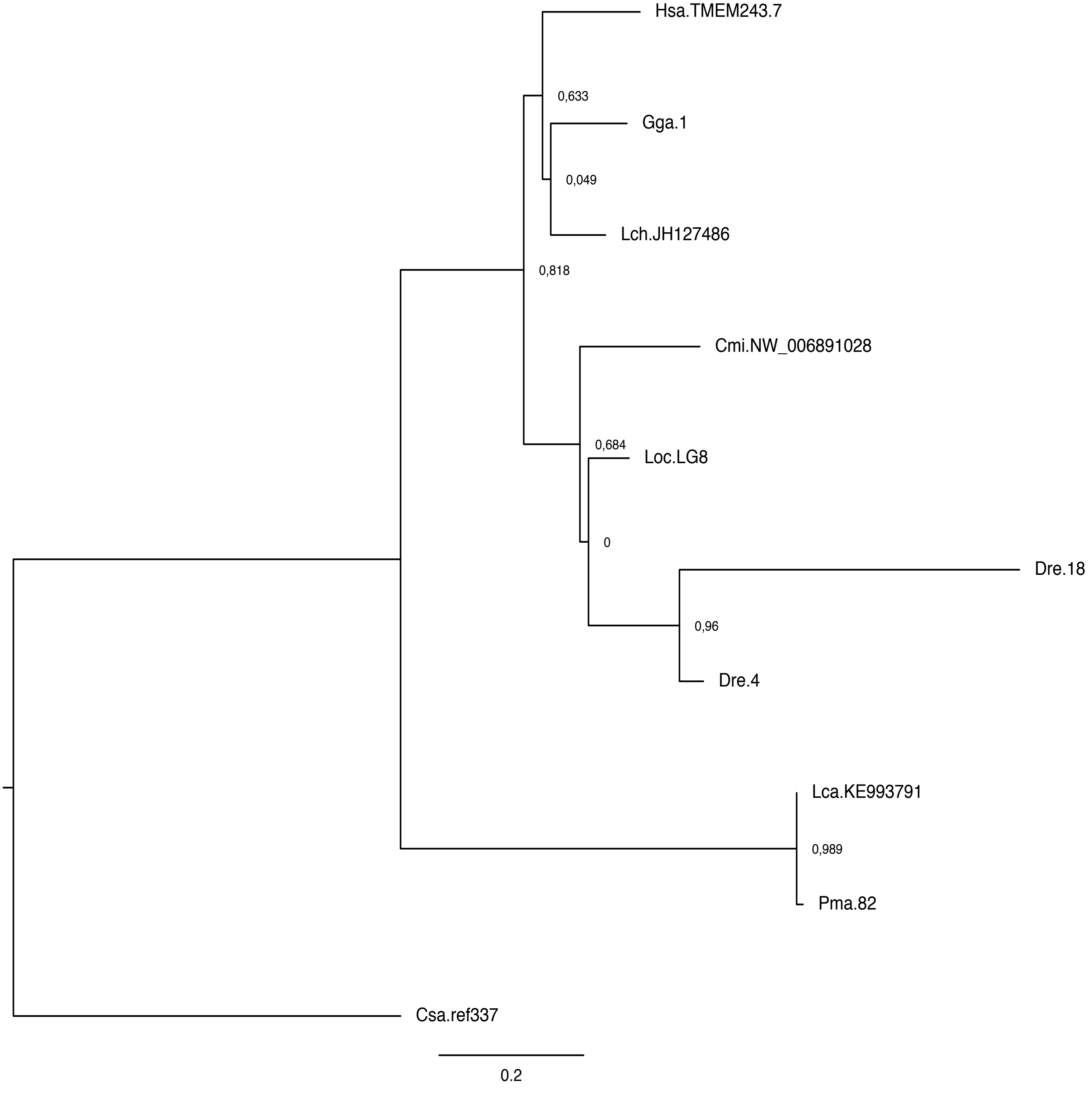

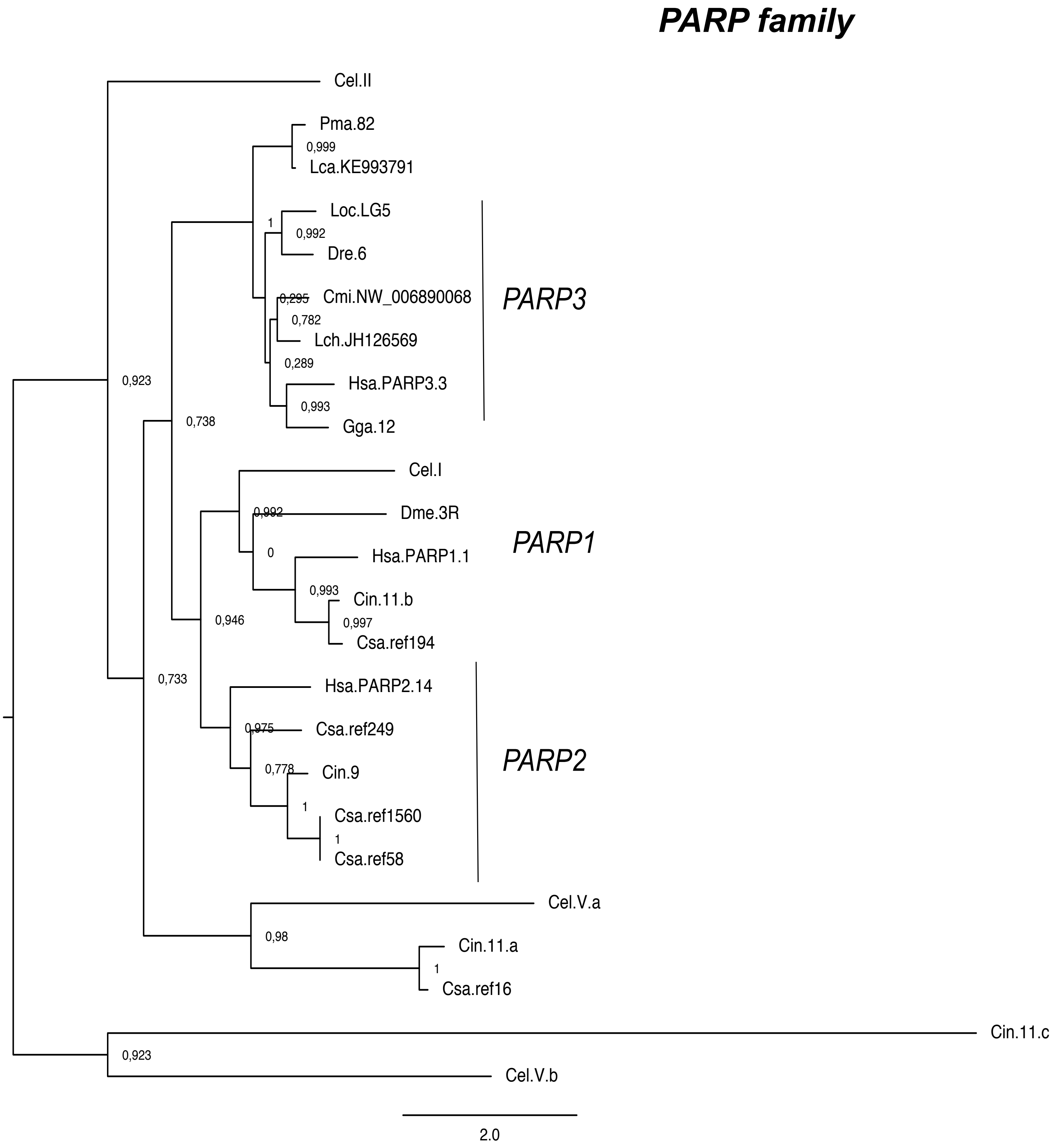
PARP family
Cel.II
Pma.82
0,999
Lca.KE993791
Loc.LG5
1
0,992
Dre.6
0,295
Cmi.NW_006890068
0,782
Lch.JH126569
0,289
Hsa.PARP3.3
0,993
Gga.12
PARP3
0,923
0,738
Cel.I
0,992
Dme.3R
0
Hsa.PARP1.1
0,993
Cin.11.b
0,997
Csa.ref194
PARP1
0,946
0,733
Hsa.PARP2.14
0,975
Csa.ref249
0,778
Cin.9
1
Csa.ref1560
1
Csa.ref58
PARP2
Cel.V.a
0,98
Cin.11.a
1
Csa.ref16
Cin.11.c
0,923
Cel.V.b
2.0

CPM family

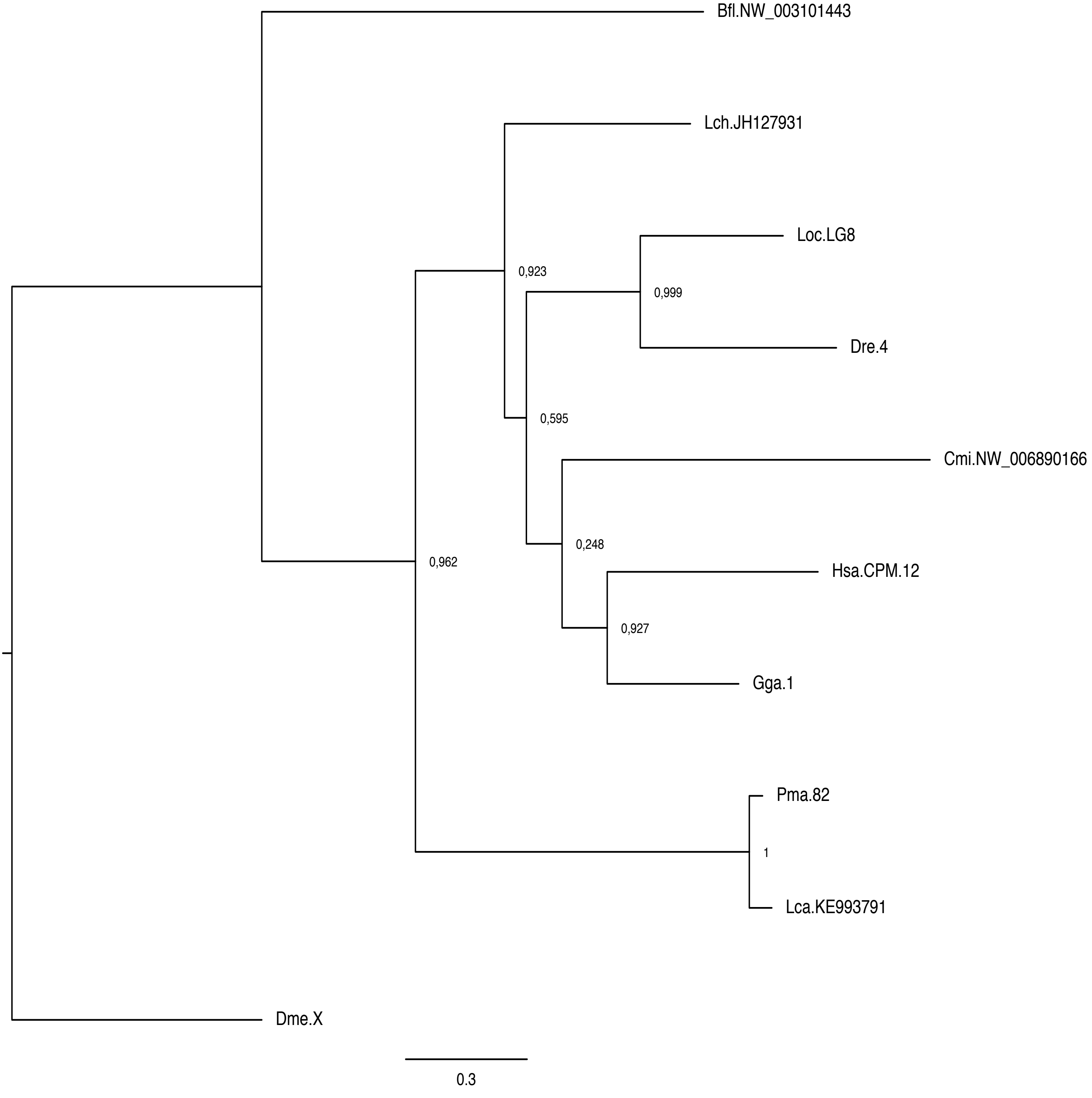

Bfl.NW_003101443
Lch.JH127931
Loc.LG8
0,923
0,999
Dre.4
0,595
Cmi.NW_006890166
0,248
0,962
Hsa.CPM.12
0,927
Gga.1
Pma.82
1
Lca.KE993791
Dme.X
0.3
