# Supplementary figures and images for: Corticotropin-Releasing Hormone (CRH) Gene Family Duplications in Lampreys Correlate With Two Early Vertebrate Genome Doublings
Source: Front Neurosci. 2020 Jul 30;14:672. doi: 10.3389/fnins.2020.00672 (PMC7406891; doi:10.3389/fnins.2020.00672)

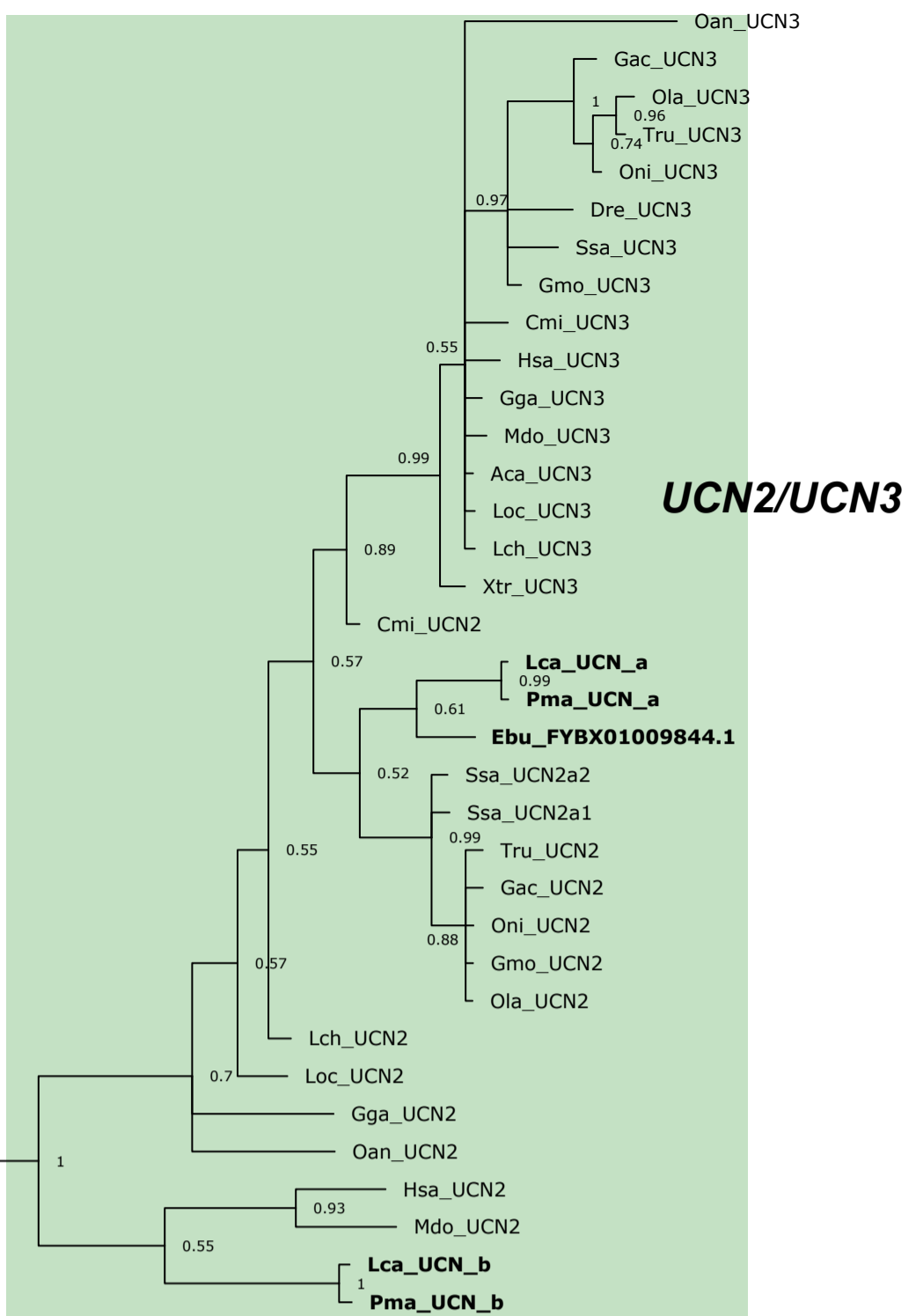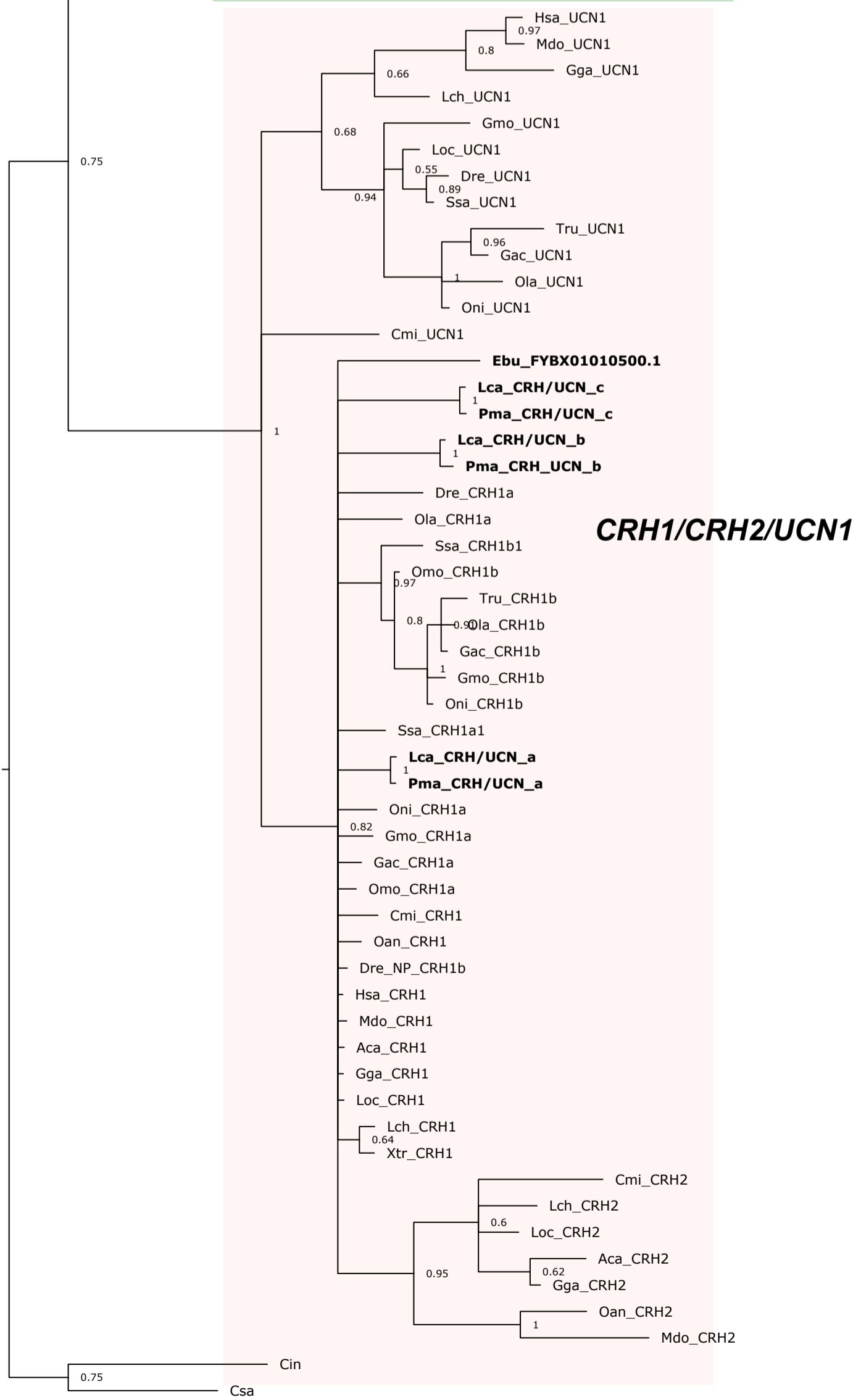

Supplement: FIGURE S1 — Bayesian inference tree of the CRH mature peptides. Branch support values (posterior probability values) are shown and tree was rooted with the tunicate CRH-like precursors. Tree was rooted with the tunicate CRH-family members. A simplified radial tree is represented in Figure 1. Accession numbers of the sequences used are available in Supplementary Table S2. [file Data_Sheet_1.zip › Figure S1.pdf]

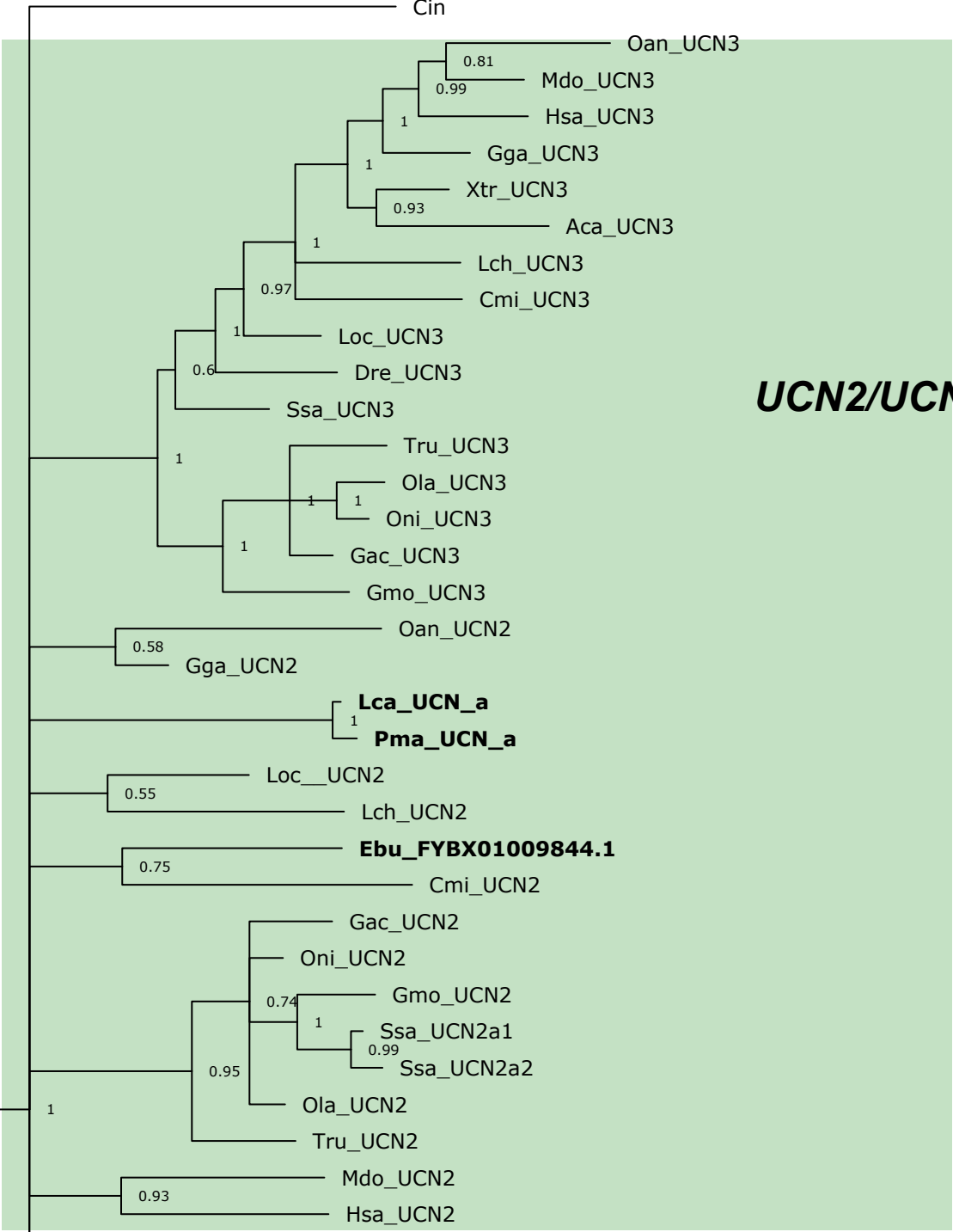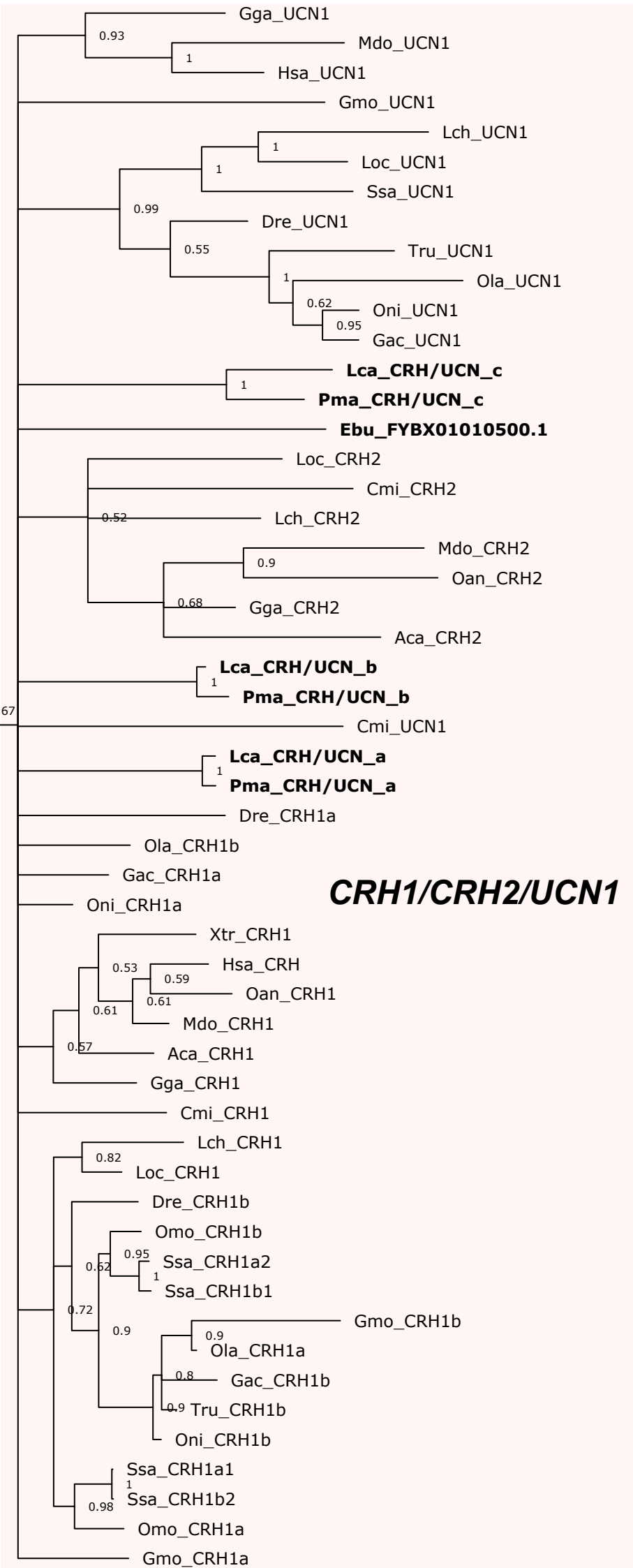

Csa

Supplement: FIGURE S1 — Bayesian inference tree of the CRH mature peptides. Branch support values (posterior probability values) are shown and tree was rooted with the tunicate CRH-like precursors. Tree was rooted with the tunicate CRH-family members. A simplified radial tree is represented in Figure 1. Accession numbers of the sequences used are available in Supplementary Table S2. [file Data_Sheet_1.zip › Figure S2.pdf]

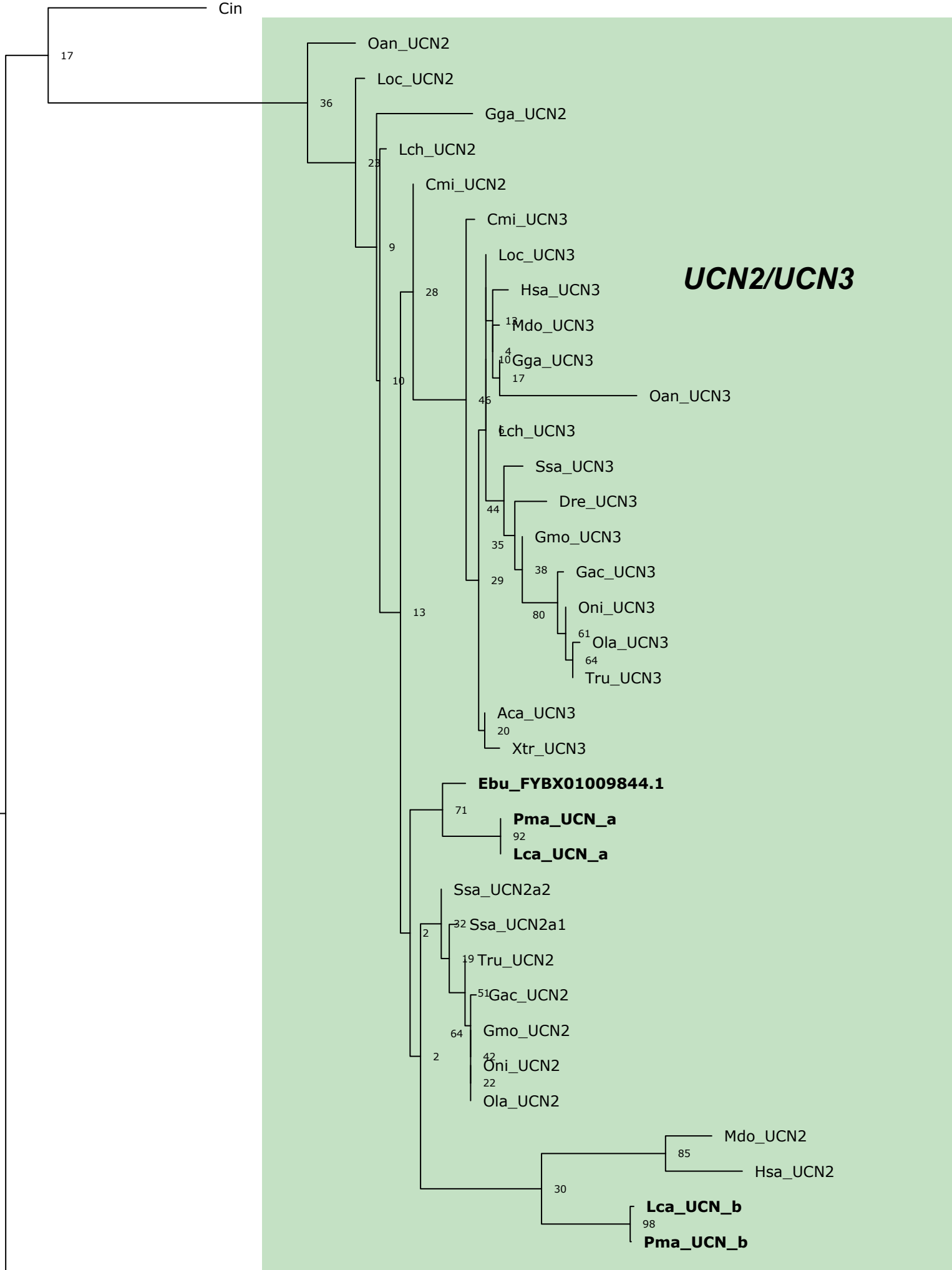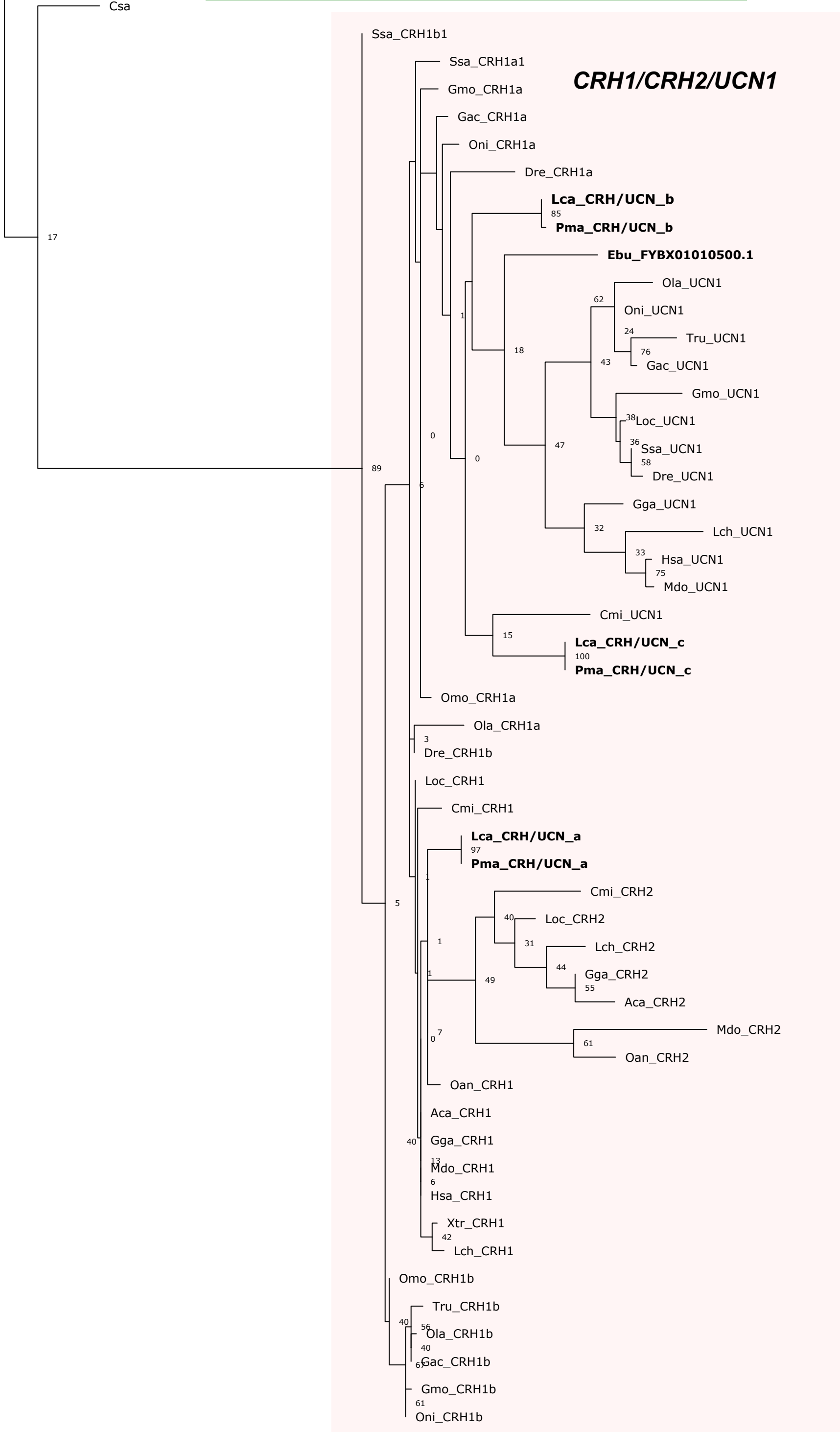

Supplement: FIGURE S1 — Bayesian inference tree of the CRH mature peptides. Branch support values (posterior probability values) are shown and tree was rooted with the tunicate CRH-like precursors. Tree was rooted with the tunicate CRH-family members. A simplified radial tree is represented in Figure 1. Accession numbers of the sequences used are available in Supplementary Table S2. [file Data_Sheet_1.zip › Figure S3.pdf]

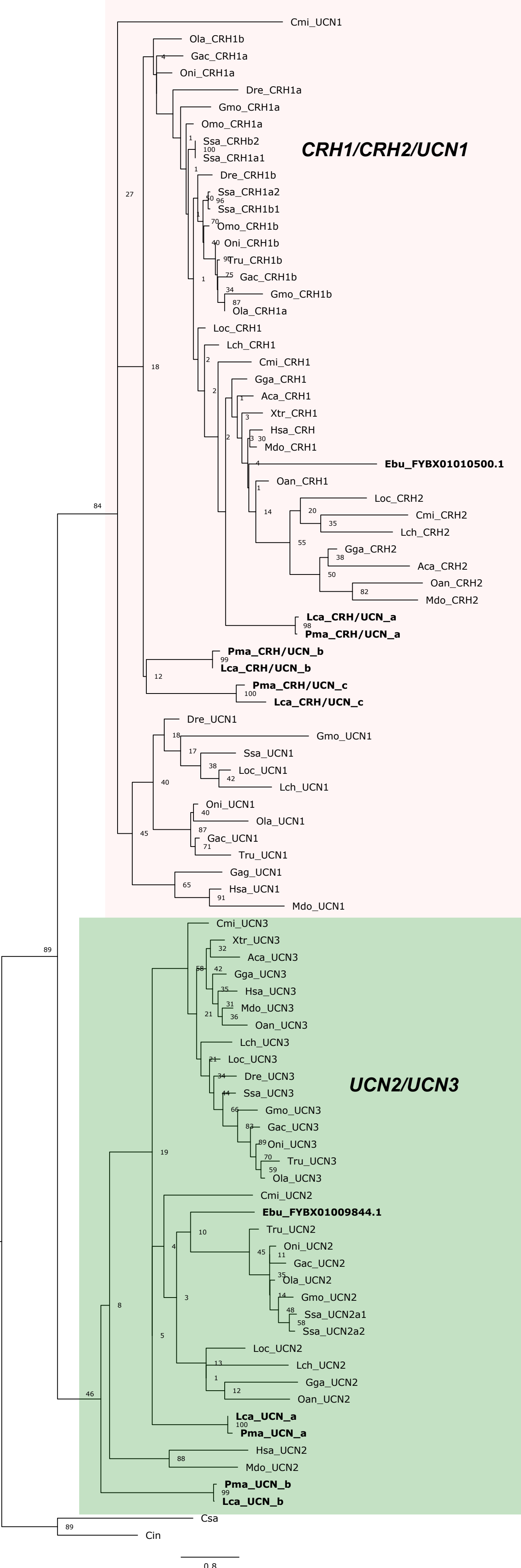

Supplement: FIGURE S1 — Bayesian inference tree of the CRH mature peptides. Branch support values (posterior probability values) are shown and tree was rooted with the tunicate CRH-like precursors. Tree was rooted with the tunicate CRH-family members. A simplified radial tree is represented in Figure 1. Accession numbers of the sequences used are available in Supplementary Table S2. [file Data_Sheet_1.zip › Figure S4.pdf]
