# Supplementary material for: Corticotropin-Releasing Hormone (CRH) Gene Family Duplications in Lampreys Correlate With Two Early Vertebrate Genome Doublings
Source: Front Neurosci. 2020 Jul 30;14:672. doi: 10.3389/fnins.2020.00672 (PMC7406891; doi:10.3389/fnins.2020.00672)
Supplement: TABLE S1 — Size (base pairs, bp) of the sea lamprey and Arctic lamprey CRH-family scaffolds. The analyzed genome regions of the sea lamprey scaffolds were selected using as starting point the position of the lamprey CRH-family member and retrieving approximately 1.5 Kb upstream and downstream of this gene. [file Table_1.DOCX]

**Supplementary Table 1:**

| Sea lamprey | bp (total) | Arctic lamprey | bp (total) |
| --- | --- | --- | --- |
| sc_00057 | 5,818,967 | KE993813 | 1,564,629 |
| sc_00003 | 25,736,823 | KE993827 | 1,417,095 |
| sc_00017 | 15,686,775 | KE993984 | 623,865 |
| sc_00040 | 10,986,173 | KE994104 | 391,907 |
| sc_00082 | 1,961,081 | KE993959 | 702,280 |
