# Supplementary material for: Corticotropin-Releasing Hormone (CRH) Gene Family Duplications in Lampreys Correlate With Two Early Vertebrate Genome Doublings
Source: Front Neurosci. 2020 Jul 30;14:672. doi: 10.3389/fnins.2020.00672 (PMC7406891; doi:10.3389/fnins.2020.00672)
Supplement: TABLE S2 — Accession numbers of all the CRH-family members used in phylogeny. The genome localization (chromosome and position) is give. ni, not identified; na, not available; un, uncharacterized. [file Table_2.docx]

|  | **CRH1** | **CRH2** | **UCN1** | **UCN2** | **UCN3** | **CRH/UCN1** | **UCN2/UCN3** |
| --- | --- | --- | --- | --- | --- | --- | --- |
| **TETRAPODS** |  |  |  |  |  |  |  |
| Human  (*Homo sapiens,* Hsa) | NP_000747  *Chr 8_66.176* | ni | NP_003344  *Chr 2_27.307* | NP_149976  *Chr3_48.5* | NP_444277  *Chr10_5.36* |  |  |
| Opossum  (*Monodelphis domestica*, Mdo) | ENSMODP00000036360  *Chr 3_171* | na  *Chr 1_472* | ENSMODP00000028973  Chr 1_508.59 | na  *Chr 6_194* | ENSMODP00000023062 *Chr8_121.44* |  |  |
| Platypus  *(Ornithorhynchus anatinus*, Ona) | ENSOANP00000003650  *Chr 7_1.6* | *Ultra337*_*3* | ni | na  *Ultra 32_0.7* | na  *Contig13_0.6* |  |  |
| Chicken  (*Gallus gallus*, Gga) | ENSGALG00000037148  *Chr 2_115* | ENSGALG00000051589  *Chr 20_9.51* | ENSGALG00000054781  *Chr 3_104.5* | na  *Chr 12_9* | ENSGALG00000032978  *Chr1_1.84* |  |  |
| Lizard  (*Anolis carolinensis*, Ano) | ENSACAP00000016393  *Chr 4_30* | na  *Chr 4_156* | ni | ni | ENSACAP00000002612 *Chr5_3.75* |  |  |
| Xenopus  (*Xenopus tropicalis*, Xtr) | ENSXETP00000058657 *GL173273.1_0.555* | ni | ni | ni | ENSXETP00000035550 *GL172647.1_1.43* |  |  |
| **LOBE-FINNED FISH** | | | | | | | |
| Coelacanth  (*Latimeria chalumnae*, Lch) | ENSLACP00000002936 *JH128518.1_0.54557* | na  *JH129487.1_0.06* | ENSLACP00000011213 *JH127070.1_0.45* | na  *JH126690.1_1* | na  *JH126940.1_1.33* |  |  |
| **RAY-FINNED FISH** | | | | | | | |
| Spotted gar  (*Lepisosteus oculatus*, Loc) | ENSLOCP00000021917  *LG 9_6* | na  *LG 18_11* | ENSLOCP00000022093 *LG1_17.175* | na  *LG 5_51* | ENSLOCP00000022005 *LG8_11.31* |  |  |
| Tilapia  (*Oreochromis niloticus*, Oni) | a-ENSONIP00000025716 *GL831416.1_0. 3789*  b-ENSONIP00000025725 *GL831157.1_0.8489* | ni | ENSONIP00000026599 *GL831530.1_0.242* | *GL831152.1 _4* | ENSONIP00000025826 *GL831167.1_2.25* |  |  |
| Fugu  (*Takifugu rubripes,* Tru) | b-ENSTRUP00000022019 *scaffold 95_0.421* | ni | ENSTRUP00000030463 *Scaffold 120_0.06* | na  *Scaffold 116_0.09* | ENSTRUP00000040554  *Scaffold 8_1.19* |  |  |
| Zebrafish  (*Danio rerio*, Dre) | a-ENSDARP00000118835  *Chr 2_42.21*  b-NP_001007380  *Chr 24_25.45* | ni | NP_001025351 *Chr_20_38.749* | ni | NP_001076423  *Chr4_15.05* |  |  |
| Stickleback  (*Gasterosteus aculeatus,* Gac) | a-ENSGACP00000003885  *groupXXI_7306566*  b-na  *scaffold_120_91380* | ni | ENSGACP00000008094  *groupXVIII_3.30* | na  *groupXVII_9.84* | ENSGACP00000026067  *groupIV_2.95* |  |  |
| Cod  (*Gadus morhua*, Gmo) | a-ENSGMOG00000020152 *GeneScaffold_378_17550*  b-ENSGMOT00000002317  *GeneScaffold_2511_7808* | ni | ENSGMOP00000017865  *GeneScaffold_2370_0.02* | ENSGMOP00000021625  *GeneScaffold_904_0.02* | ENSGMOT00000013071  *GeneScaffold_1619_0.11* |  |  |
| Medaka  (*Oryzias latipes*, Ola) | a-NP_001121990  *Chr20_15301114*  b-XP_011484530  *Chr17_10775330* | ni | NP_001295911.1 *Chr24_0.260* | NP_001121991.1  *Chr 5_3.18* | NP_001121992.1  *Chr23_1.53* |  |  |
| Salmon  (*Salmo salar*, Ssa) | a1-NP_001135062.1  a2-XP_014036838.1  b1-ACM09696.1  b2-XP_014045819.1 | ni | AGKD01034474.1 | a1-AGKD01002194.1  a2-AGKD01007057.1 | AGKD01004252.1 |  |  |
| Smelt  (*Osmerus mordax*, Omo) | a-ACO09991.1  b-ACO10011.1 | un | un | un | un |  |  |
| **JAWLESS FISH** |  |  |  |  |  |  |  |
| Elephant shark  (*Callorhinchus milii,* Cmi) | XP_007894939.1  *NW_006890101.1_2.53* | XP_007909389.1  *NW_006890059.1_0.56* | XP_007902667.1  *NW_006890177.1_1.27* | XP_007888748.1 *NW_006890068 _4.10* | XP_007889031.1  *NW_006890070.1_1.88* |  |  |
| **AGNATHA** |  |  |  |  |  |  |  |
| Arctic lamprey  (*Lethenteron camtschaticum,* Lca) |  |  |  |  |  | KE994103.1 *Scaffold00432_0.06* | KE993813.1 *Scaffold00142_0.52* |
|  |  |  |  |  |  | KE993984.1 *Scaffold00313_0.22* | KE993959.1  *Scaffold00288_0.628* |
|  |  |  |  |  |  | KE993827.1 *Scaffold00156_1.15* |  |
|  |  |  |  |  |  |  |  |
| Sea lamprey  (*Petromyzon marinus,* Pma) |  |  |  |  |  | na  *Scaffold00040_5.38* | na  *Scaffold00057_1.81* |
|  |  |  |  |  |  | na  *Scaffold00017_3.93* | na  *Scaffold00082_0.92* |
|  |  |  |  |  |  | na  *Scaffold00003_7.68* |  |
| Hagfish  (*Eptatretus burgeri,* Ebu) |  |  |  |  |  | ENSEBUG00000003988  *FYBX02010500.1_5.62* | ENSEBUG0000001565*6FYBX02009844.1_2.19* |
